# Non-relapse mortality with bispecific antibodies: A systematic review and meta-analysis in lymphoma and multiple myeloma

**DOI:** 10.1016/j.ymthe.2025.03.048

**Published:** 2025-03-31

**Authors:** Tobias Tix, Mohammad Alhomoud, Roni Shouval, Gloria Iacoboni, Edward R. Scheffer Cliff, Doris K. Hansen, Saad Z. Usmani, Gilles Salles, Miguel-Angel Perales, David M. Cordas dos Santos, Kai Rejeski

**Affiliations:** 1 Department of Medicine III – Hematology/Oncology, LMU University Hospital, LMU Munich, Munich, Germany; 2 Adult Bone Marrow Transplantation Service, Department of Medicine, Memorial Sloan Kettering Cancer Center, New York, NY, USA; 3 Department of Medicine, Weill Cornell Medical College, New York, NY, USA; 4 Department of Hematology, University Hospital Vall d’Hebron, Vall d’Hebron Institute of Oncology (VHIO), Barcelona, Spain; 5 Department of Medicine, Universitat Autònoma de Barcelona, Bellaterra, Spain; 6 Department of Medical Oncology, Dana-Farber Cancer Institute, Boston, MA, USA; 7 Harvard Medical School, Boston, MA, USA; 8 Department of Clinical Haematology, Peter MacCallum Cancer Centre and Royal Melbourne Hospital, Melbourne, Australia; 9 Department of Blood and Marrow Transplant and Cellular Immunotherapy, Moffitt Cancer Center, Tampa, FL, USA; 10 Myeloma Service, Department of Medicine, Memorial Sloan Kettering Cancer Center, New York, NY, USA; 11 Lymphoma Service, Department of Medicine, Memorial Sloan Kettering Cancer Center, New York, NY, USA; 12 Broad Institute of Massachusetts Institute of Technology (MIT) and Harvard University, Cambridge, MA, USA; 13 German Cancer Consortium (DKTK), Partner Site Munich, a partnership between the DKFZ Heidelberg and LMU University Hospital, Munich, Germany

**Keywords:** bispecific antibodies, non-relapse mortality, infections, CAR T-cell therapy, meta-analysis, lymphoma, multiple myeloma

## Abstract

Bispecific antibodies (BsAb) are associated with distinct immune-related toxicities that impact morbidity and mortality. This systematic review and meta-analysis examined non-relapse mortality (NRM) with BsAb therapy in B-cell non-Hodgkin lymphoma (NHL) and multiple myeloma (MM). A PubMed and Embase search up to October 2024 identified 29 studies (21 NHL, 8 MM) involving 2,535 patients. The overall NRM point estimate was 4.7% (95% confidence interval [CI] 3.4%–6.4%), with a median follow-up of 12.0 months. We noted no significant difference in NRM across disease entities (NHL: 4.2%, MM: 6.2%, *p* = 0.22). In NHL, prespecified subgroup analyses revealed increased NRM in real-world studies compared to clinical trials. For MM, an association between NRM and higher response rates and longer follow-up was noted. Meta-regression comparing BsAb and CAR-T therapies (*n* = 8,592) showed no significant NRM difference when accounting for key study-level confounders (*p* = 0.96). Overall, infections were the leading cause of NRM, accounting for 71.8% of non-relapse deaths. Of the infection-related deaths, 48% were attributed to COVID-19. In a pre-specified sensitivity analysis excluding COVID-19 fatalities, the overall NRM estimate was 3.5% (95% CI 2.6%–4.6%). Taken together, these results provide a benchmark for the estimated NRM with BsAb therapy and highlight the paramount importance of infection reporting, prevention, and mitigation.

## Graphical abstract

**Figure:**
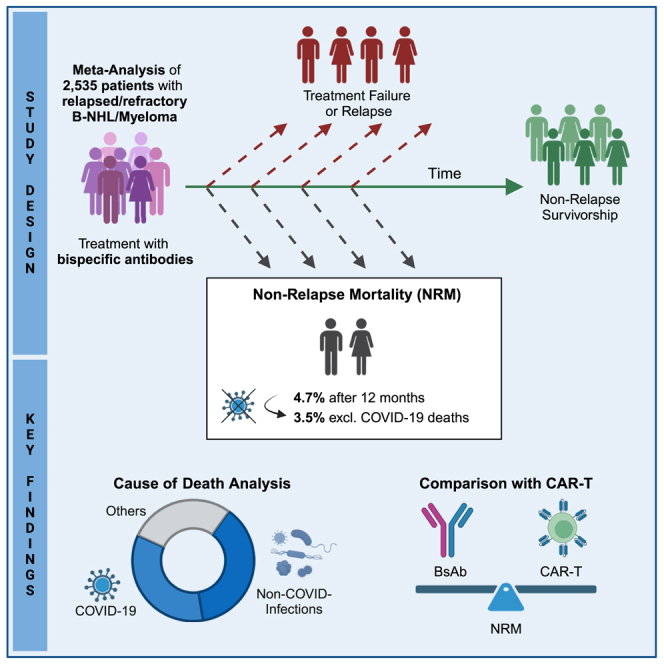

## Teaser

This systematic review and meta-analysis examined non-relapse mortality, referring to death unrelated to cancer relapse/progression, in patients with lymphoma and myeloma treated with bispecific antibodies. The overall NRM was 4.7%, with infections (especially COVID-19) as the leading cause. Rejeski and colleagues’ findings emphasize the need for improved infection reporting, prevention, and mitigation.

## Introduction

Bispecific antibodies (BsAb) represent a modern immunotherapeutic approach, linking CD3 on T-cells to tumor-associated antigens on cancer cells, thereby enabling direct targeting of malignant cells without the need for *ex vivo* cell manipulation.^1^^,^^2^ While the most extensively studied BsAb targeting CD19, CD20, GPRC5D, and BCMA have so far been used to treat patients with relapsed or refractory lymphoid and plasma cell malignancies,^3^^,^^4^ multiple studies are currently investigating their efficacy and safety in earlier lines of treatment^5^ and for non-malignant conditions like autoimmune diseases.^6^ BsAb have emerged as both an alternative and an adjunct to chimeric antigen receptor (CAR) T-cell therapies. They offer the advantages of being off the shelf, with easier administration and the potential for repeated dosing in settings where treatment can be paused upon response and resumed upon relapse.^7^^,^^8^^,^^9^

While BsAb and CART-cell therapies share T-cell activation and cytotoxicity mechanisms, they differ in their adverse event profiles. Both approaches are associated with immune-related toxicities such as cytokine release syndrome (CRS) and immune effector cell-associated neurotoxicity syndrome (ICANS). However, the incidence and severity of these toxicities tend to be lower with BsAb compared to CAR T-cell therapies, possibly due to the absence of the large-scale T-cell expansion characteristic of CAR T-cell therapy.^10^^,^^11^^,^^12^ However, infectious complications are a significant and concerning toxicity associated with BsAb therapy.^13^^,^^14^ These infections often arise from on-target/off-tumor effects, such as B-cell aplasia and hypogammaglobulinemia, leading to sustained immunosuppression and increased infection susceptibility.^15^^,^^16^ Additionally, factors such as neutropenia, T-cell exhaustion, prior chemotherapy, and the underlying malignancy further contribute to this vulnerability.^11^^,^^13^^,^^17^

Given the cumulative burden of these potentially fatal toxicities, understanding non-relapse mortality (NRM), defined as death not preceded by disease recurrence or progression, associated with BsAb therapy is essential. While NRM is a well-established metric in allogeneic hematopoietic cell transplantation (HCT) and has drawn growing attention in the context of CAR T-cell therapies,^18^^,^^19^^,^^20^ data on NRM associated with BsAb therapies remain limited.

This systematic review and meta-analysis addresses this gap by analyzing the incidence and causes of NRM associated with BsAb therapy across a spectrum of advanced B-cell malignancies. Additionally, we leverage the insights gained from our previous analysis of NRM with CAR T-cell therapy,^18^ to provide a comprehensive perspective on immunotherapy-related mortality risks across treatment modalities.

## Results

### Study cohort

**Figure 1:**
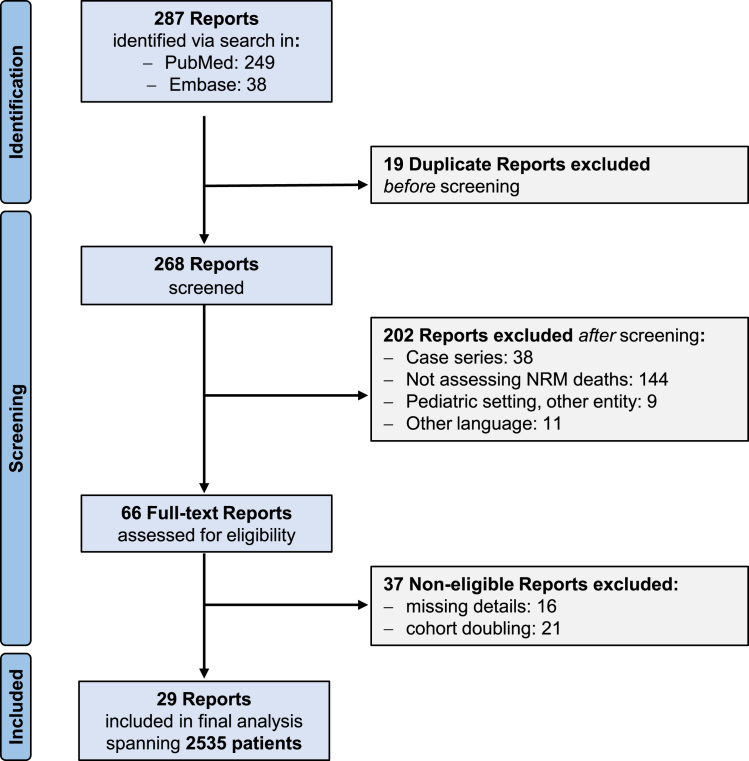
Study retrieval and identification for meta-analysis Flowchart illustrating the inclusion and exclusion process for the systematic review and meta-analysis of non-relapse mortality (NRM) associated with BsAb therapy, conducted in accordance with PRISMA guidelines.

We screened a total of 287 studies for reports of non-relapse deaths in patients treated with BsAb therapies. Of these, 66 full-text articles were assessed for NRM and causes of death, and 29 articles met the criteria for further analysis (Figure 1). These included 25 reports on clinical trials (CTs),^9^^,^^21^^,^^22^^,^^23^^,^^24^^,^^25^^,^^26^^,^^27^^,^^28^^,^^29^^,^^30^^,^^31^^,^^32^^,^^33^^,^^34^^,^^35^^,^^36^^,^^37^^,^^38^^,^^39^^,^^40^^,^^41^^,^^42^^,^^43^^,^^44^ encompassing a total of 2,340 patients (phase 1: 7, phase 1/2: 5, phase 2: 13), and 4 real-world studies (RW)^45^^,^^46^^,^^47^^,^^48^ involving 195 patients (Table 1). Two CTs were subdivided by dose-specific^27^^,^^38^ and one CT by disease-specific cohorts,^9^ resulting in 32 distinct study cohorts for final evaluation.

**Table 1:** Characteristics of included records

Entity	First author	Year	Cohort	Product	Setting	Cohort size	Non-relapse deaths	NRM point estimate, %	Follow- up, months	Age, y, median	Prior treatment lines, median	Prior CAR-Ts, %	ORR, %	Treatment mode
IL	Linton	2024	A	epcoritamab	2	128	12	9.38	17.4	65	3	5	82.0	exp
	Linton	2024	B	epcoritamab	2	86	0	0.00	5.7	64	2	7	86.0	exp
	Budde	2024	A1	mosunetuzumab	1/2	67	2	2.99	39.6	60	3	6	65.7	esc+exp
	Budde	2022		mosunetuzumab	2	90	1	1.11	18.3	60	3	3	80.0	NA
	Kim	2024		odronextamab	2	128	18	14.06	20.1	61	3	0	NR	NA
LBCL	Guieze	2024		blinatumomab	2	25	0	0.00	23.2	66	1	NR	36.0	cons
	Coyle	2020		blinatumomab	2	41	3	7.32	4.9	56	2	0	37.0	NA
	Viardot	2016		blinatumomab	2	23	1	4.35	11.7	66	3	NR	43.0	NA
	Katz	2022		blinatumomab	2	28	1	3.57	12	64	0	0	89.3	cons
	Izutsu	2023		epcoritamab	2	36	0	0.00	8.4	68.5	3	0	55.6	NA
	Thieblemont	2022		epcoritamab	1/2	157	9	5.73	10.7	64	3	38.9	63.1	exp
	Hsu	2024		glofitamab	RW	31	3	9.68	15.9	58	5	NR	56.0	NA
	Song	2024		glofitamab	1	27	0	0.00	15	57.5	2	20	66.7	exp
	Atesoglu	2023		glofitamab	RW	43	5	11.63	5.7	54	4	0	37.0	NA
	Dickinson	2022		glofitamab	2	154	8	5.19	12.6	66	3	33	52.0	NA
	Budde	2024	A2	mosunetuzumab	1/2	129	8	6.20	40.8	64	3	11.6	36.4	esc+exp
	Budde	2024	B	mosunetuzumab	2	98	3	3.06	23.9	68	2	35.7	59.2	plus pola
	Olszewski	2023		mosunetuzumab	2	40	1	2.50	32	65	0	0	87.5	plus CHOP
	Bartlett	2023		mosunetuzumab	2	88	3	3.41	10.1	66.5	3	29.5	42.0	NA
MCL	Philipps	2024		glofitamab	1/2	60	9	15.00	19.6	72	2	3.3	85.0	NA
NHL	Goebeler	2016		blinatumomab	1	34	1	2.94	NR	62	3	NR	NR	exp
	Hutchings	2021		glofitamab	1	171	2	1.17	13.5	64	3	1.8	53.8	esc+exp
	Bannerji	2022		odronextamab	1	145	7	4.83	4.2	67	3	29	51.0	esc+exp
MM	D’Souza	2022		ABBV-383 (etentamig)	1	51	1	1.96	8.2	68	4	0	59.0	exp
	Bahlis	2023		elranatamab	1	55	5	9.09	12	64	5	9.1	63.6	exp
	Lesokhin	2023		elranatamab	2	123	14	11.38	14.7	68	5	0	61.0	NA
	Bumma	2024		linvoseltamab	1/2	117	6	5.13	1^a^	70	5	NR	70.9	exp
	Chari	2022	A	talquetamab	1	30	0	0.00	11.7	62	6	NR	70.0	exp
	Chari	2022	B	talquetamab	1	44	3	6.82	4.2	64	5	NR	64.0	exp
	Lebreton	2024		teclistamab	RW	15	1	6.67	5.4	68	4	NR	NR	NA
	Dima	2024		teclistamab	RW	106	3	2.83	3.8	66.5	6	40	66.0	NA
	Moreau	2022		teclistamab	1/2	165	19	11.52	14.1	64	5	0	63.0	exp

1, phase 1 trial; 1/2, phase 1/2 trial; 2, phase 2 trial; IL, indolent lymphoma; LBCL, large B-cell lymphoma and other aggressive lymphomas; MCL, mantle cell lymphoma; MM, multiple myeloma; NA, not applicable; NHL, B-cell non-Hodgkin lymphoma (when subcohorting in other mentioned entities was not possible based on the reported data); NR, not reported; NRM, non-relapse mortality; ORR, overall response rate; RW, real-world analysis. In the column “treatment mode,” additional information on the treatment is given: cons, bispecific antibody (BsAb) therapy as a consolidation concept after another treatment regimen; exp, dose expansion cohort of early clinical trials; exp + esc, mixed cohort of dose escalation and dose expansion (further subcohorting and inclusion of only dose expansion cohort not possible based on reported data); plus CHOP, combination therapy of BsAb with CHOP chemotherapy; plus pola, combination therapy of BsAb with polatuzumab-vedotin.

a Follow-up was restricted to 1 month, given that NRM cases were only reported during this period.

The most common entity was large B-cell lymphoma (LBCL, 920 patients), followed by multiple myeloma (MM, 706 patients), indolent lymphoma (IL, 499 patients), and mantle cell lymphoma (MCL, 60 patients). Three studies did not specify lymphoma subtypes (350 patients).^22^^,^^31^^,^^33^ For non-Hodgkin lymphoma (NHL), the distribution of BsAb products was 6 glofitamab, 6 mosunetuzumab, 5 blinatumomab, 4 epcoritamab, and 2 odronextamab. Among the MM cohorts, 3 were treated with teclistamab, 2 with talquetamab, 2 with elranatamab, and 1 each with linvoseltamab and ABBV-383 (etentamig). Follow-up duration ranged from 1.0 to 40.8 months across studies.

### NRM estimates did not vary significantly across disease entities and BsAb products

**Figure 2:**
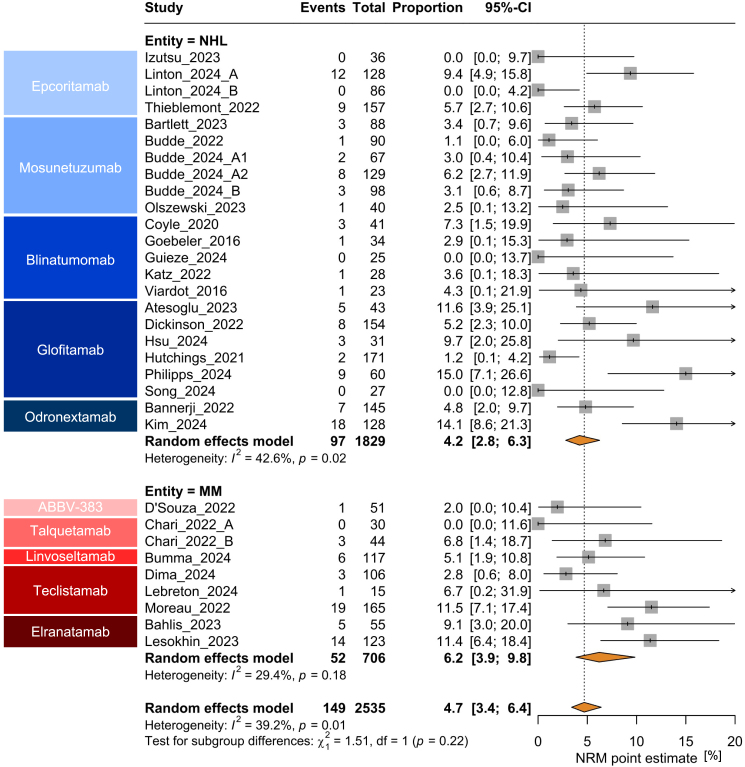
Forest plot of NRM point estimates across all study cohorts and stratified by entity Forest plot displaying NRM point estimates and 95% confidence intervals, calculated using a random effects model. Studies are arranged by disease type, with B-cell non-Hodgkin lymphoma (NHL, blue) presented at the top and multiple myeloma (MM, red) below. Different shades of blue and red represent therapeutic products within each disease category. The disease-specific and the overall NRM point estimates are emphasized in bold black. Measures of heterogeneity, including *I*^2^ values, are provided (with *I*^2^ values between 25% and 50% indicating low-to-moderate heterogeneity).

Across all patients, the overall NRM point estimate was 4.7% (95% confidence interval [CI] 3.4%–6.4%; Figure 2) after a median follow-up of 12.0 months. Incorporating the median follow-up per study, we observed 0.043 NRM events per patient-year (Figure S1). Study heterogeneity was moderate (*I*^2^ = 39.2%; Figure 2).^49^ While we identified a risk of publication bias by funnel plot analysis (Egger’s test *p* < 0.001; Figure S2), we did not detect a risk of study bias, except incomplete reporting of ethnicity (Table S1). Since underlying tumor biology and previous treatments may affect non-relapse deaths, we next investigated associations between tumor entities and NRM point estimates.

**Figure 3:**
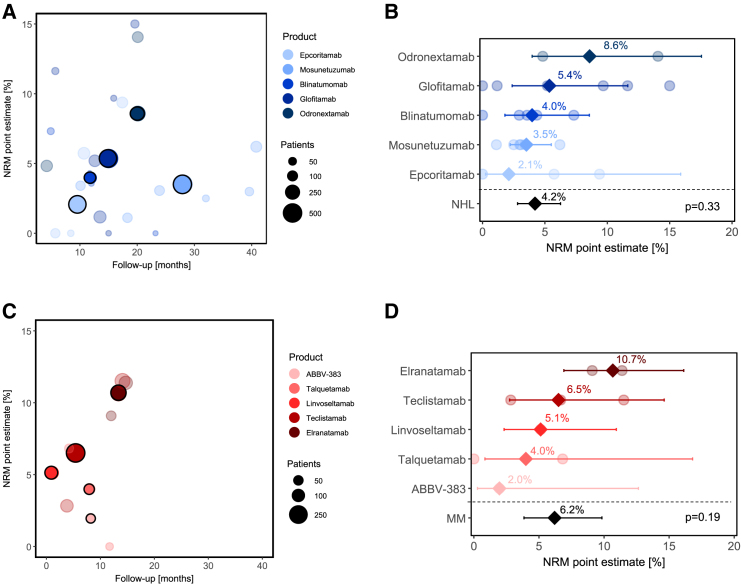
Disease-specific NRM point estimates for different BsAb products (A and C) Bubble plots showing NRM point estimates relative to follow-up duration for NHL (blue) and MM (red). Different shades of blue and red represent therapeutic products within each disease category. Each bubble represents a cohort, with bubble size proportional to the cohort’s total patient count. Aggregated NRM point estimates for all cohorts within one entity are encircled in black. (B and D) Comparison of aggregated NRM point estimates and 95% CIs across the different BsAb products for NHL (B) and MM (D). The *p* values for the comparisons of NRM point estimates by disease entity and BsAb product were calculated using the test for subgroup differences (random effects model).

While NRM was numerically lower with NHL (4.2%, 95% CI 2.8%–6.3%), we did not detect a significant difference compared to MM (6.2%, 95% CI 3.9%–9.8%, *p* = 0.22; Figure 2). Furthermore, the different products did not significantly impact the NRM estimates; there was neither a significant difference between NHL products (odronextamab 8.6%, glofitamab 5.4%, blinatumomab 4.0%, mosunetuzumab 3.5%, epcoritamab 2.1%, *p* = 0.33; Figures 3A and 3B) nor between MM products (elranatamab 10.7%, teclistamab 6.5%, linvoseltamab 5.1%, talquetamab 4.0%, ABBV-383 [etentamig] 2.0%, *p* = 0.19; Figures 3C and 3D). In NHL, the pre-specified subgroup analyses revealed significantly higher NRM point estimates in the RW studies compared to the cohorts treated within clinical trials (Figure S3A), but no significant impact of age, prior CAR-T exposure, number of previous therapy lines, overall response rate, or the duration of follow-up on the NRM point estimates (panel A of Figures S4–S8). In MM, significantly higher NRM point estimates were observed in cohorts with overall response rates above the median (9.9% vs. 4.0%, *p* = 0.005; Figure S7B) and follow-up time above the median (10.2% vs. 4.2%, *p* = 0.003; Figure S8B), but no significant impact of the treatment setting (CT vs. RW), age, or prior CAR-T exposure (panel B of Figures S3–S5). Between the different target antigens, no difference in NRM point estimates was observed either in NHL (CD19 vs. CD20) or in MM (BCMA vs. GPRC5D) (Figure S9). In the two NHL studies included in our analysis where BsAb were combined with other therapeutic regimens, NRM was not significantly higher with combination therapy (Figure S10).^24^^,^^40^ Additionally, there was no significant difference in NRM point estimates between products that are already approved and those not yet approved for the investigated entity (Figure S11).

### Meta-regression analysis reveals a similar NRM for BsAb and CAR-T therapy when accounting for key study-level confounders

**Figure 4:**
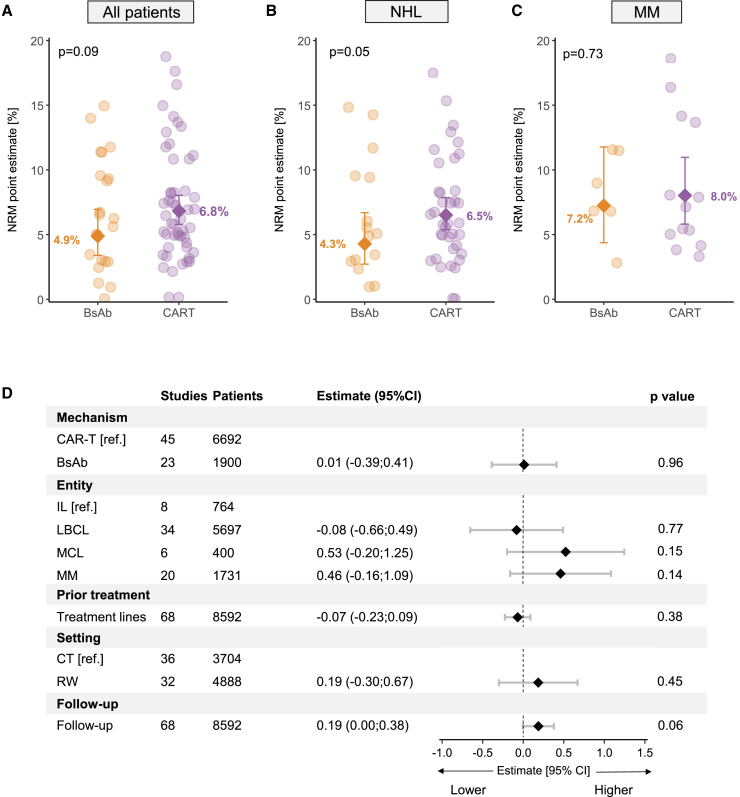
Comparative analysis of NRM point estimates between BsAb and CAR T-cell therapy (A–C) Aggregated NRM point estimates and 95% CIs are compared between BsAb- and CAR-T-treated patients across all disease entities (A) and specifically for NHL (B) and MM (C). (D) Multivariable meta-regression analysis using random effects models for study characteristics. The integrated forest plot displays model estimates, with 95% CI for each study variable, such as mechanism (CAR-T vs. BsAb), disease entity, treatment setting, median number of treatment lines, follow-up time (years). Reference levels for the calculation of model estimates and respective *p* values are provided for each study feature.

While both BsAb and CAR T-cell therapies hold significant promise, the lack of direct comparative studies poses a challenge to optimizing clinical decision-making and understanding their relative safety profiles. To address this gap, we integrated our previously published systematic review on NRM after CAR T-cell therapy.^18^ To align the inclusion criteria of both studies, for this meta-regression we excluded BsAb that were not yet approved by the US Food and Drug Administration (FDA) or the European Medicines Agency for the investigated disease entity at the time of submitting this study. On univariate analysis, the NRM point estimate was numerically lower with BsAb therapy (4.9% vs. 6.8%, *p* = 0.09; Figure 4A). Subgroup analysis indicated that this difference was primarily driven by NHL studies (4.3% vs. 6.5%, *p* = 0.05; Figure 4B), whereas NRM estimates were elevated for both treatment modalities in MM (BsAb: 7.2%, CAR-T: 8.0%, *p* = 0.73; Figure 4C). Next, we performed a meta-regression analysis incorporating 68 study cohorts and 8,592 patients adjusted for disease type, prior treatment lines, treatment setting, and follow-up duration, excluding 3 studies that did not differentiate between lymphoma subtypes. When accounting for these key study-level risk factors, the treatment modality had no statistically significant effect on NRM estimates (*p* > 0.9; Figure 4D). Notably, extended follow-up emerged as the only study-level covariate showing a trend toward influencing NRM (*p* = 0.06).

When excluding COVID-19 fatalities, we noted more pronounced differences between both treatment modalities (3.6% vs. 6.1%, *p* = 0.003; Figure S12). For example, significantly lower NRM estimates were seen for BsAb vs. CAR-T therapy in NHL (3.1% vs. 5.8%, *p* = 0.005), which also extended to numerically decreased NRM in MM (5.0% vs. 7.2%, *p* = 0.16). In addition, a trend toward lower NRM was observed with BsAb compared to CAR-T therapy in the meta-regression analysis, excluding COVID-19 deaths (adjusted *p* = 0.10; Table S2).

### Infections are the main driver of NRM following BsAb therapy

**Figure 5:**
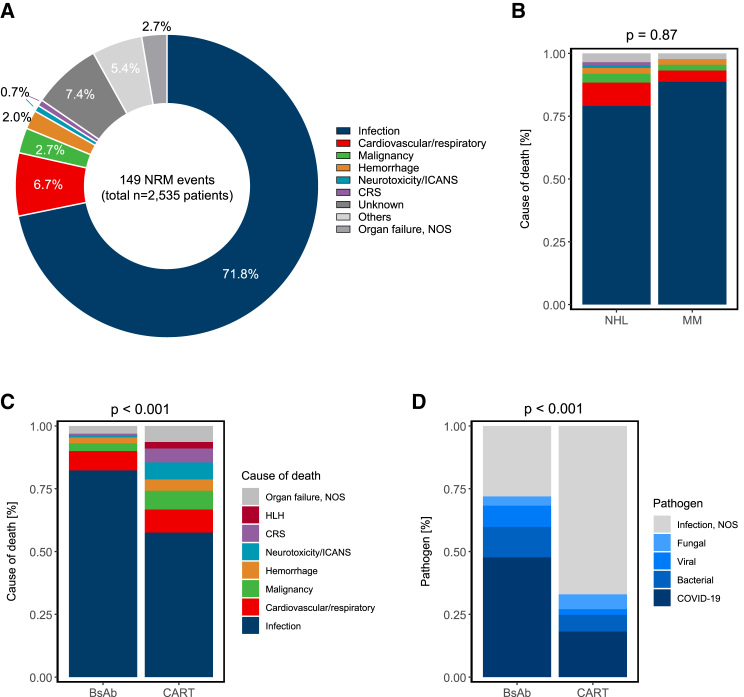
Distribution of causes of NRM (A) Donut plot illustrating the distribution of causes of death in the entire BsAb study cohort. Specific causes of death are represented in distinct colors, while undefined and unclassifiable causes are shown in shades of gray. (B) Comparison of NRM causes between NHL and MM patients. (C and D) Comparison of NRM causes (C) and the distribution of pathogens responsible for fatal infections (D) between BsAb- and CAR-T cell-treated patients. Statistical significance was evaluated using the χ^2^ distribution test.

To elucidate NRM etiology, we extracted the available data for all 149 reported non-relapse deaths among our total BsAb study cohort of 2,535 patients. For 138/149 cases (92.6%), the underlying cause of death was indicated, which we classified into one of 7 groups as outlined in the materials and methods (Figure 5A). If the cause of death did not match any of these groups, it was classified under “other” (8/149 cases; Table S3). In 11/149 cases (7.4%), the specific cause of death was reported as “unknown.”

Interestingly, more than 70% of all reported non-relapse deaths were attributed to infections (107/149, 71.8%). The cause of infection-related death was not further specified in 30 cases. Among the 77 non-relapse deaths where the pathogen was reported, the majority were attributed to COVID-19 infection (51/77, 66.2%). Of note, most of the included studies accrued during the COVID-19 pandemic (Table S4). In a pre-specified sensitivity analysis excluding COVID-19-related deaths, we noted an overall NRM point estimate of 3.5% (95% CI 2.6%–4.6%) for our study cohort (Table S5). Still, a significant proportion of NRM cases in patients receiving BsAb were due to bacterial (13/77, 16.9%), non-COVID viral (9/77, 11.7%), or fungal infections (4/77, 5.2%). A detailed breakdown of pathogens resulting in death due to infection is provided in Table S6, including some rarer opportunistic infections such as five cases of progressive multifocal leukoencephalopathy (PML), four cases of adenoviral infections, and three cases of *Pneumocystis jirovecii* pneumonia (PJP). We were not able to resolve whether these specific patients had previous CAR-T exposure.

Cardiovascular or respiratory (CVR) events were the second most common cause of NRM, resulting in 10/149 (6.7%) non-relapse deaths. Four non-relapse deaths were attributed to either a secondary malignancy unrelated to the underlying lymphoma/myeloma or organ failure, each accounting for 2.7% of cases. Three additional deaths (2.0%) resulted from hemorrhages. The prototypical immunotoxicities ICANS and CRS each led to only 1 of the 149 non-relapse deaths (0.7% each).

When comparing NHL and MM, we observed no significant difference in the distribution of causes of death (Figure 5B) or pathogens responsible for the infection-related deaths (Figure S13). However, we noted a significantly different cause-of-death pattern with BsAb compared to CAR-T treatment, with a relative skewing toward infection-related deaths in the BsAb group (Figure 5C). A closer look at the cause attribution for infection-related deaths revealed that there were fewer cases classified as not otherwise specified (NOS) in the BsAb cohort (Figure 5D), indicating improved infection reporting relative to CAR-T therapy.

## Discussion

In this systematic review and meta-analysis, we outline the comparative incidence and causes of NRM with bispecific antibodies across a spectrum of hematologic malignancies. Our findings highlight a consistent NRM point estimate of 4.7% across studies, with no significant differences observed across disease entities or BsAb products. While NRM point estimates were numerically lower with BsAb vs. CAR-T treatment, this observation did not extend to the meta-regression analysis accounting for key study-level risk factors, including follow-up. Infections were by far the main cause of NRM, responsible for over 70% of non-relapse deaths.

The introduction of BsAb into clinical practice has broadened the therapeutic landscape for patients with relapsed or refractory B-cell malignancies. Their off-the-shelf availability and broad applicability make them an attractive option, particularly in scenarios where CAR T-cell therapy or HCT may not be feasible due to logistical, medical, or socioeconomic factors.^3^^,^^8^ As these therapies gain traction, NRM as a measure of treatment-associated safety becomes a critical component in informing patient-centered decision-making.

Relative to CAR-T therapy, BsAb carry a generally favorable safety profile with mainly low-grade CRS and anecdotal ICANS.^12^^,^^50^ However, our analysis found that this did not translate into a significantly reduced NRM when controlling for key study-level risk factors, highlighting the critical importance of infections in heavily pre-treated patients. However, it should be noted that many of the BsAb studies accrued during the COVID-19 pandemic and that follow-up on many of these studies remains short. Indeed, the NRM differences between CAR-T and BsAb therapy were more pronounced upon exclusion of COVID-19 fatalities in our study (Figure S12). Generally, the observed NRM estimates with BsAb therapy were lower or at least comparable to autologous^51^^,^^52^^,^^53^^,^^54^^,^^55^ and lower compared to allogeneic HCT,^56^^,^^57^^,^^58^^,^^59^ with implications for treatment sequencing.^60^^,^^61^

The comparatively low NRM and favorable early immunotoxicity profile suggests that BsAb therapy not only offers at least comparable safety to other established treatments but also holds promise for integration into combination regimens, potentially enhancing therapeutic outcomes without significantly increasing NRM. In line with this, the two NHL studies where BsAb were utilized within combination regimens did not yield significantly higher NRM (Figure S10).^24^^,^^40^ One study evaluated BsAb in combination with polatuzumab, a well-tolerated antibody-drug conjugate,^24^ while the other focused on BsAb combined with the combination of cyclophosphamide, doxorubicin hydrochloride (hydroxydaunorubicin), vincristine sulfate (Oncovin), and prednisone (CHOP) as a first-line treatment.^40^ However, the randomized STARGLO trial (published following completion of our study) suggested that adding a BsAb to existing chemotherapies in the relapsed or refractory setting, while efficacious, may also increase the risk of fatal infections.^62^ Among the 15 deaths during treatment (representing 8.3% of the experimental arm), 10 were related to infection (7 due to COVID-19), illustrating the cumulative infection risk in these patients.

Another advantage of bispecific antibody therapy, in contrast to CAR-T and HCT, is the ability to discontinue the treatment if necessary or reduce the dose or frequency of administration, providing an additional layer of flexibility in managing treatment-related toxicities.^63^ However, the cumulative exposure to B cell-depleting therapy over long periods of time may potentiate infection risk^64^ and facilitate chronic T-cell exhaustion.^17^

In this meta-analysis, infections accounted for 71.8% of all non-relapse deaths with BsAb therapy. Notably, COVID-19 was a significant contributor, accounting for approximately two-thirds of infection-related fatalities with an identifiable pathogen. Much of the data analyzed was collected during the COVID-19 pandemic (2020–2023), which may have in part influenced the observed NRM rates. Indeed, the prespecified sensitivity analysis excluding COVID-19-related deaths yielded a lower NRM estimate of 3.5% (Table S5). These findings underscore the importance of preventing infections, particularly through comprehensive vaccination against SARS-CoV-2 and other preventable pathogens like *pneumococcus*. Additionally, rare opportunistic infections such as PML, PJP, and adenoviral infections contributed to fatalities, highlighting the heightened vulnerability of these patients. Due to the nature of our study, it was impossible to ascertain whether these specific patients had also received prior CAR T-cell therapy, which can confer long-lasting T-cell deficits.^65^^,^^66^^,^^67^ Examining lymphocyte subpopulations before BsAb treatment may be prescient and could guide the use and duration of antimicrobial prophylaxis, especially for *Pneumocystis*.^14^^,^^50^

Various factors heighten the susceptibility of patients receiving bispecific antibodies to infectious complications, including Tcell exhaustion, prior chemotherapy, and the underlying malignancy.^11^^,^^13^^,^^17^ Furthermore, neutropenia and on-target/off-tumor effects, such as B-cell aplasia and hypogammaglobulinemia, further elevate the risk of infections.^15^^,^^16^ Addressing these vulnerabilities is critical, and supportive strategies, such as granulocyte-colony-stimulating factor and immunoglobulin (Ig) substitution, may help mitigate these risks.^14^^,^^15^^,^^50^^,^^68^ The importance of prophylactic measures is underscored by findings from the RedirecTT-1 trial of teclistamab/talquetamab, which reported infection-related deaths in 11 of 94 patients, yet reveals inconsistent implementation of prophylaxis, with approximately 18% of patients not receiving antiviral prophylaxis, around 50% lacking protection against PJP, and 43% of those with non-IgG myeloma never receiving IgG replacement therapy.^69^ In particular, Ig substitution should be strongly considered as a key strategy to lower NRM.^15^ In multiple myeloma, for example, prophylactic intravenous Ig (IVIG) is recommended for patients experiencing recurrent bacterial infections or with IgG levels below 400 mg/dL.^64^^,^^70^ Consensus recommendations of an expert panel from the Academic Consortium to Overcome Multiple Myeloma through Innovative Trials advocate for IVIG as primary prophylaxis, independent of IgG levels, starting in the second month of treatment and continuing until either therapy completion or IgG levels exceed 400 mg/dL, whichever occurs later.^16^ However, the available data from the included studies lack the necessary detail to determine the impact of consistent IVIG use on patient outcomes (Table S4). This highlights the necessity of standardized documentation and adherence to prophylactic strategies to better understand their role in reducing NRM and improving patient outcomes.

Our analysis revealed key limitations in the current body of evidence, particularly the heterogeneous reporting of NRM endpoints. Cumulative NRM rates, critical for understanding the trajectory of mortality risk over time in relation to other competing events like relapse or progression,^19^^,^^71^^,^^72^^,^^73^ were not at all reported. Moreover, categorization inconsistencies across studies may have introduced variability. Standardized definitions and reporting guidelines for infections are essential to improve data reliability and comparability, as recently implemented for immune effector cell-associated hemophagocytic lymphohistiocytosis-like syndrome (IEC-HS), immune effector cell-associated enterocolitis, and immune effector cell–associated hematotoxicity (ICAHT).^74^^,^^75^^,^^76^^,^^77^^,^^78^ Another challenge is the limited availability of robust real-world data. For example, the existing and included RW data were confined to glofitamab for NHL and teclistamab for MM. Clinical trial cohorts often exclude patients with poor performance status or significant comorbidities, limiting the generalizability of findings. Additionally, our meta-analysis could account only for cohort-level differences and not individual patient factors, such as tumor burden, systemic inflammation, number of BsAb cycles, or prior therapies, which likely influence NRM risk.

Methodological limitations relate to the publication bias detected in the Egger’s test and the discrepancy in the inclusion periods for BsAb and CAR-T studies, potentially affecting comparisons. While a subgroup analysis comparing BsAb records published after March 31, 2024 with those published before found no significant differences regarding NRM point estimates (Figure S14), the different search window may still have impacted our results. Of note, we did not detect significant effects from factors such as the inclusion of unapproved BsAb or their use as part of combination therapies. These studies were retained to provide a more comprehensive and real-world perspective on treatment outcomes, although certain biases and limitations due to their inclusion cannot be entirely eliminated. Despite these limitations, we believe that the overall analysis remains robust, and the findings reflect a clinically relevant, broad view of BsAb therapy. To test the robustness of our main study findings, we performed not only several subgroup analyses (Figures S3–S11), but also several sensitivity analyses (Table S6), showing stable results. For example, NRM estimates were comparable when applying fixed-effect vs. random-effect models.

As BsAb are increasingly introduced earlier in treatment lines, NRM becomes a critical factor in guiding their sequencing and evaluating their safety profile relative to other treatment options. This is particularly important for patients ineligible for more intensive therapies, where minimizing treatment-related mortality is paramount. Future studies should prioritize robust and detailed reporting of NRM, particularly with respect to infection-related deaths, to better inform clinical decision-making. Such reports should encompass the infection type (viral, bacterial, fungal), confirmation through microbiological testing, identified pathogens, the timing of infection, infection source and severity, and whether the infection occurred early vs. late in the treatment process. Collecting this information can help further refine existing evidence-based guidelines focused on infection prevention and management for patients receiving BsAb therapy.^14^^,^^50^^,^^64^^,^^70^ Efforts should focus on identifying subgroups at heightened risk of NRM and tailoring supportive care interventions to their needs. For example, differentiating between time-limited therapies and those administered until severe toxicity is observed may further refine the balance between efficacy and safety.

In conclusion, our analysis highlights the importance of improving BsAb safety reporting and infection management. With infections being the leading cause of NRM across all disease types and products, further developing evidence-based guidelines for infection prevention, including vaccination, Ig substitution, and antimicrobial prophylaxis, is crucial. Enhanced reporting and long-term follow-up will enable a more nuanced understanding of NRM and its drivers, ultimately supporting safer use of BsAb in hematological malignancies.

## Materials and methods

### Study design and literature search

The methods used in this study were aligned with a previous meta-analysis conducted by our group.^18^ We screened all studies for the FDA-approved bispecific antibodies in MM (teclistamab, talquetamab, elranatamab) and NHL (glofitamab, epcoritamab, mosunetuzumab) and unapproved bispecific antibodies for these malignancies whose phase 1 and/or 2 results have been fully published (odronextamab, blinatumomab, ABBV-383 [etentamig], linvoseltamab). A systematic search was performed in the PubMed and Embase databases for articles published up to October 10, 2024, using combined key words for each of the bispecific antibodies along with “lymphoma” or “myeloma” (see Data S1). Case studies, reviews, conference abstracts, and meta-analyses were excluded. After screening titles and abstracts, eligible publications were assessed by two independent investigators (T.T. and M.A.) based on the following set of inclusion criteria that had to be fully satisfied.

(1) Adult cancer patients with either IL, LBCL, MM, or MCL
(2) Use of bispecific antibody products approved by the FDA, or a bispecific antibody with phase 1 and/or 2 results published as a full manuscript
(3) Cohort of at least 15 patients treated with the same dose of the bispecific antibody
(4) Reporting of the absolute number of non-relapse deaths in the treated cohort
(5) Full-text available in English

All included articles were examined for potential duplicate reporting. In cases where two studies covered the same patient population, the study with longer follow-up was selected.

For the comparison with NRM in CAR-T cell-treated patients, we incorporated records from our previous meta-analysis on CAR-T-treated myeloma and lymphoma patients, with screening and extraction methods detailed there.^18^ The inclusion criteria were similar, with publications included until March 31, 2024.

This study followed the PRISMA(-P) guidelines (see Data S1) and was prospectively registered to the PROSPERO database on December 27, 2023 (study no. CRD42023494258).^79^ The PRISMA checklist can be provided by the authors upon reasonable request. Institutional review board approval was not sought as this study did not represent human participant research.

### Data extraction

Data collection was performed by two independent investigators (T.T. and M.A.) and included date of publication, number of patients, disease entities, utilized bispecific antibody, time frame of patient inclusion, median follow-up time, median age of the cohort, treatment history (median number of previous lines, proportion of patients with prior HCT or CAR-T therapy), treatment setting (RW study vs. CT, including trial phase), and overall response rate (ORR). Furthermore, we determined whether BsAb were applied as monotherapy, combination therapy, or consolidation therapy. The primary outcome was the number and causes of death. If a patient’s remission status at the time of death was not clearly specified, then the death was considered an NRM case. If multiple dosing schemes were used or multiple entities were treated within a single study, then the reported data were assigned to separate cohorts and analyzed individually, provided that the quality of reporting allowed for such separation. Dose escalation cohorts were excluded from analysis where separation was possible.

### Quality assessment

The Joanna Briggs Institute appraisal tool was applied to assess study bias of included articles (Table S1). Visual inspection of funnel plot asymmetry and Egger regression tests were used to assess reporting bias (Figure S2).^80^^,^^81^

### Statistical analysis

Data analysis was conducted in R (version 4.4.1) using the metafor (version 4.6-0) and meta (version 8.0-1) packages. NRM point estimates and estimates for NRM per patient-year were derived by performing random-effects meta-analyses of single proportions through a generalized linear mixed model.^82^ Patient-years were calculated using the number of included patients and the median follow-up time in years of each respective cohort. The Clopper-Pearson interval provided 95% CIs for proportions.^83^ Forest plots were used to visually represent NRM outcome data. For each meta-analysis, heterogeneity of pooled effect sizes was assessed with the Q statistic and quantified using *I*^2^, where 25%, 50%, and 75% reflect low, moderate, and high between-study heterogeneity, respectively.^84^ Continuous variables were reported as medians and interquartile ranges. The chi-squared (χ^2^) test was used to examine the distribution of NRM-related deaths across sub-cohorts.

### Meta-analyses

Separate meta-analyses were conducted for predefined subgroups to compare NRM point estimates based on disease entity, BsAb product, treatment setting, age, the number of prior therapy lines, prior CAR-T exposure, overall response rate, and follow-up. Subgroup comparisons were evaluated using the test for subgroup differences within a random effects model, and corresponding *p* values were calculated.

### Meta-regression analysis

A meta-regression analysis was performed to examine the association between NRM point estimates and the following variables: treatment modality (BsAb vs. CAR-T), disease entity, treatment setting (e.g., CT vs. RW), treatment line, and follow-up time. The meta-regression model was calculated based on random effects models using the maximum likelihood estimator.^85^ Individual model coefficients and respective CIs were tested using the Knapp-Hartung method.^86^ The stability of model estimates was validated by performing permutation testing.^87^

### Sensitivity analysis

To validate the robustness of the main findings, meta-analyses were repeated after (1) testing fixed effects models, (2) excluding COVID-related deaths, and (3) excluding phase 1 studies where it was not feasible to extract a subcohort of patients treated with a single-dose scheme.^88^

### Cause of death analysis

To test for cause of death distributions between subgroups, causes of death were classified into one of the following groups: infection, secondary malignancy, CRS, ICANS, cardiovascular or respiratory, hemorrhage, organ failure NOS, other, or unknown. Infections were further classified into COVID-19, bacterial, viral, fungal, or infection NOS. Donut plots to visualize the cause of death distribution were generated using GraphPad Prism (version 10.4.0).

## Data availability

All data needed to evaluate the conclusions in the paper are present in the manuscript and/or the supplemental information. Data from primary studies are publicly available within the databases listed in the supplemental information. All codes were adapted using R software, version 4.4.1 (meta package 8.0–1, metafor package 4.6–0). If there are further questions, the corresponding author should be contacted.

## Acknowledgments

First and foremost, we acknowledge the numerous patients whose data contributed to this study, along with the dedicated research professionals driving progress in this rapidly advancing field. T.T. received a fellowship from the School of Oncology of the German Cancer Consortium (DKTK). D.M.C.d.S. received the Walter Benjamin Fellowship from the 10.13039/501100001659Deutsche Forschungsgemeinschaft (DFG, German Research Foundation, grant no. 525171148). E.R.S.C. receives research funding from 10.13039/100014848Arnold Ventures. D.K.H. is supported by the National Cancer Institute (grant no. R01CA281756-01A1) and the Pentecost Family Myeloma Research Center. K.R. acknowledges funding from the Else Kröner Forschungskolleg (EKFK) within the Munich Clinician Scientist Program (MCSP), the Bruno and Helene Jöster Foundation, and the “CAR-T Control” translational group within the Bavarian Center for Cancer Research (grant no. BZKF-#TLG-22). R.S., K.R., and M.-A.P. were supported by a 10.13039/100007052Memorial Sloan Kettering Cancer Center Core grant (P30 CA008748) from the 10.13039/100000002National Institutes of Health/10.13039/100000054National Cancer Institute. R.S. was supported by an 10.13039/100000002NIH-NCI K-award (grant no. K08CA282987). The content is solely the responsibility of the authors and does not necessarily represent the official views of the National Institutes of Health. The graphical abstract was created in BioRender (https://BioRender.com/dw8w1oc).

## Author contributions

Conceptualization: T.T. and K.R. Investigation: T.T., M.A., R.S., G.I., E.R.S.C., D.K.H., S.Z.U., G.S., M.-A.P., D.M.C.d.S., and K.R. Formal analysis and visualization: T.T. and D.M.C.d.S. Methodology: T.T., D.M.C.d.S., and K.R. Writing – original draft: T.T., D.M.C.d.S., and K.R. Writing – review & editing: T.T., M.A., R.S., G.I., E.R.S.C., D.K.H., S.Z.U., G.S., M.-A.P., D.M.C.d.S., and K.R. All authors read and approved the final manuscript.

## Declaration of interests

R.S. reports speaker honorarium from Incyte. G.I. reports honoraria and/or travel support from Abbvie, AstraZeneca, Autolus, Bristol Myers Squibb, Kite/Gilead, Miltenyi, and Novartis. D.K.H. reports research funding from Bristol Myers Squibb, Karyopharm, Kite Pharma, and Adaptive Biotech; her consulting or advisory roles include Bristol Myers Squibb, Janssen, Legend Biotech, Pfizer, Kite Pharma, AstraZeneca, and Karyopharm. S.Z.U. reports serving on an advisory committee for and receiving research funding from Janssen, GlaxoSmithKline, Gilead Sciences, Seattle Genetics, Sanofi, SkylineDX, Takeda, Celgene, Bristol Myers Squibb, Amgen, and AbbVie; serving on an advisory committee for EdoPharma, Genetech, K36 Therapeutics, Moderna, Oncopeptides, Novartis, SecuraBio, and TeneoBio; and receiving research funding from Merch, Array Biopharma, and Pharmacyclics. G.S. reports consultancy with AbbVie, BeiGene, Bristol Myers Squibb, Debiopharm, Epizyme, Genentech/Roche, Genmab, Innate Pharma, Incyte, Ipsen, Janssen, Kite/Gilead, Merck, Modex, Molecular Partners, Nurix, Orna Therapeutics, Pfizer, and Treeline; he has received research funding from AbbVie, Genentech, Genmab, Janssen, Ipsen, and Nurix. M.-A.P reports honoraria from Adicet, Allogene, Caribou Biosciences, Celgene, Bristol Myers Squibb, Equilium, Exevir, ImmPACT Bio, Incyte, Karyopharm, Kite/Gilead, Merck, Miltenyi Biotec, MorphoSys, Nektar Therapeutics, Novartis, Omeros, OrcaBio, Sanofi, Syncopation, Takeda, VectivBio AG, and Vor Biopharma; he serves on data safety monitoring boards for Cidara Therapeutics and Sellas Life Sciences and on the scientific advisory board of NexImmune; he has ownership interests in NexImmune, Omeros, and OrcaBio; and he has received institutional research support for clinical trials from Allogene, Incyte, Kite/Gilead, Miltenyi Biotec, Nektar Therapeutics, and Novartis. K.R. reports research funding, consultancy, honoraria, and travel support from Kite/Gilead; honoraria from Novartis; consultancy and honoraria from Bristol Myers Squibb/Celgene; and travel support from Pierre-Fabre.

## Supplemental information

Document S1. Figures S1–S14 and Tables S1–S6

Data S1. Systematic review and meta-analysis protocol according to PRISMA-P guidelines

Document S2. Article plus supplemental information
