# Supplementary material for: Non-relapse mortality with bispecific antibodies: A systematic review and meta-analysis in lymphoma and multiple myeloma
Source: Mol Ther. 2025 Mar 31;33(7):3163–76. doi: 10.1016/j.ymthe.2025.03.048 (PMC12266037; doi:10.1016/j.ymthe.2025.03.048)
Supplement: Document S1. Figures S1–S14 and Tables S1–S6 [file mmc1.pdf]

YMTHE, Volume *33*

Supplemental Information

Non-relapse mortality with bispecific antibodies: A systematic review and meta-analysis in lymphoma and multiple myeloma

Tobias Tix, Mohammad Alhomoud, Roni Shouval, Gloria Iacoboni, Edward R. Scheffer Cliff, Doris K. Hansen, Saad Z. Usmani, Gilles Salles, Miguel-Angel Perales, David M. Cordas dos Santos, and Kai Rejeski

Study	Events	Time	Proportion	95%-CI
Atesoglu_2023	5	20.425	0.245	[0.102; 0.588]
Bahlis_2023	5	55.000	0.091	[0.038; 0.218]
Bannerji_2022	7	50.750	0.138	[0.066; 0.289]
Bartlett_2023	3	74.067	0.041	[0.013; 0.126]
Budde_2022	1	137.250	0.007	[0.001; 0.052]
Budde_2024_A1	2	221.100	0.009	[0.002; 0.036]
Budde_2024_A2	8	438.600	0.018	[0.009; 0.036]
Budde_2024_B	3	195.183	0.015	[0.005; 0.048]
Bumma_2024	6	9.750	0.615	[0.276; 1.370]
Chari_2022_A	0	29.250	0.000	[0.001; 0.273]
Chari_2022_B	3	15.400	0.195	[0.063; 0.604]
Coyle_2020	3	16.742	0.179	[0.058; 0.556]
D'Souza_2022	1	34.850	0.029	[0.004; 0.204]
Dickinson_2022	8	161.700	0.049	[0.025; 0.099]
Dima_2024	3	33.567	0.089	[0.029; 0.277]
Guieze_2024	0	48.333	0.000	[0.001; 0.165]
Hsu_2024	3	41.075	0.073	[0.024; 0.226]
Hutchings_2021	2	192.375	0.010	[0.003; 0.042]
Izutsu_2023	0	25.200	0.000	[0.001; 0.317]
Katz_2022	1	28.000	0.036	[0.005; 0.254]
Kim_2024	18	214.400	0.084	[0.053; 0.133]
Lebreton_2024	1	6.750	0.148	[0.021; 1.052]
Lesokhin_2023	14	150.675	0.093	[0.055; 0.157]
Linton_2024_A	12	185.600	0.065	[0.037; 0.114]
Linton_2024_B	0	40.850	0.000	[0.001; 0.196]
Moreau_2022	19	193.875	0.098	[0.063; 0.154]
Olszewski_2023	1	106.667	0.009	[0.001; 0.067]
Philipps_2024	9	98.000	0.092	[0.048; 0.177]
Song_2024	0	33.750	0.000	[0.001; 0.237]
Thieblemont_2022	9	139.992	0.064	[0.033; 0.124]
Viardot_2016	1	22.425	0.045	[0.006; 0.317]
**Random effects model**	**148**		**0.043**	**[0.028; 0.067]**

Heterogeneity: $I^2 = 69.8\%$, $p < 0.001$

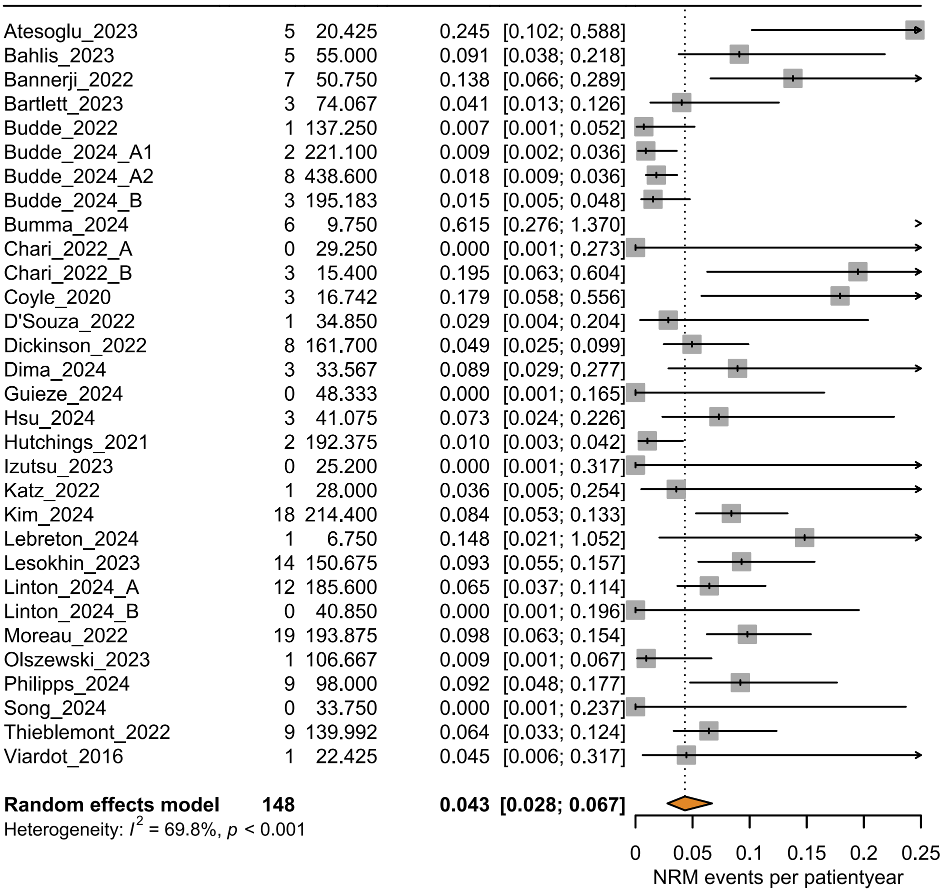

Figure S1. Forest plot of NRM per patient-year. Forest plot illustrating NRM per patient year and 95% confidence intervals (95% CI). The random effects model results and heterogeneity measures are depicted. One study was excluded from analysis since no median follow-up was reported (Goebeler et al, 2016, J Clin Oncol).

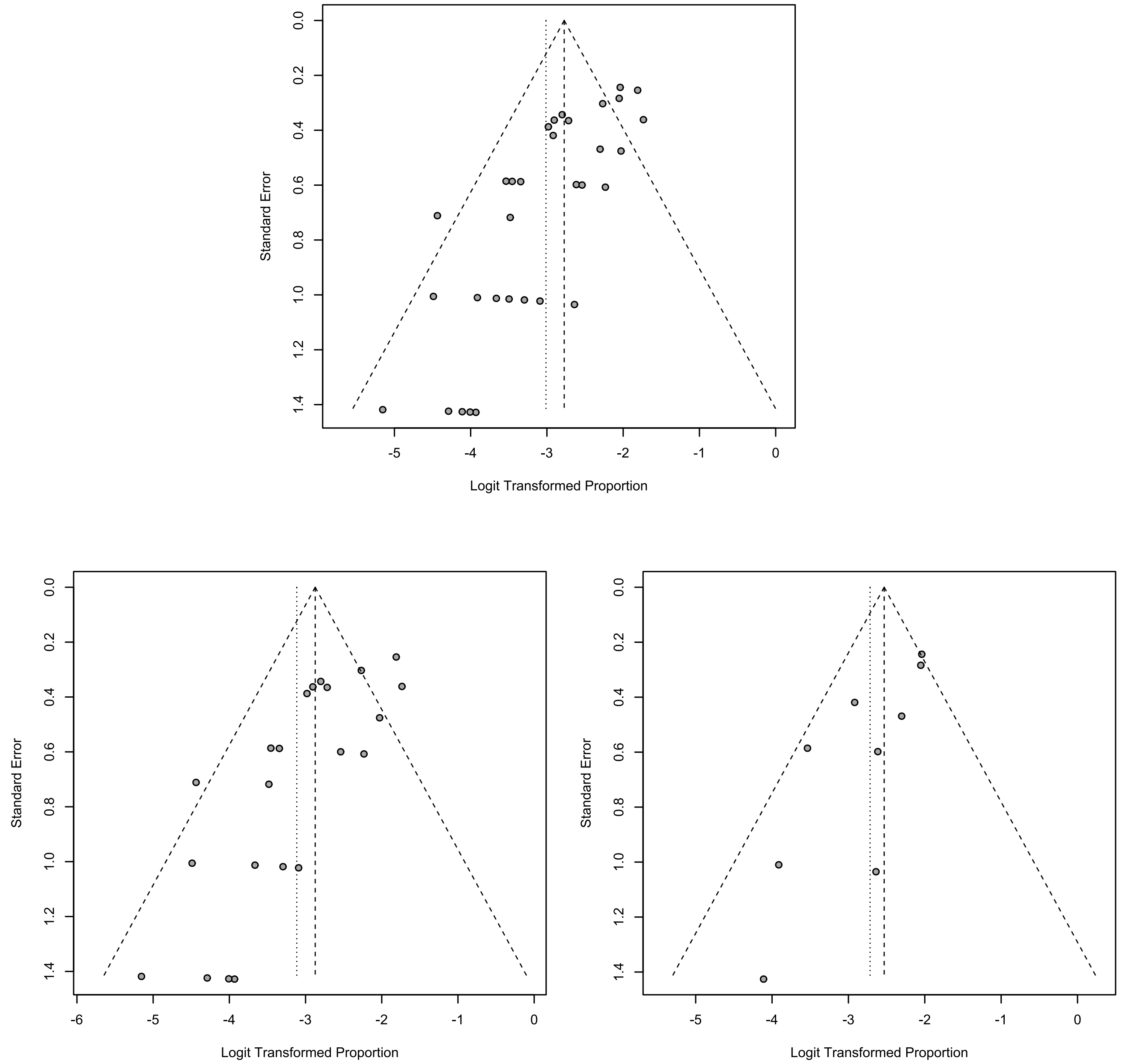

Figure S2. Funnel plots to assess reporting bias. Reporting bias was analyzed by funnel plots. P-values for funnel plot asymmetry were derived from Egger regression tests.

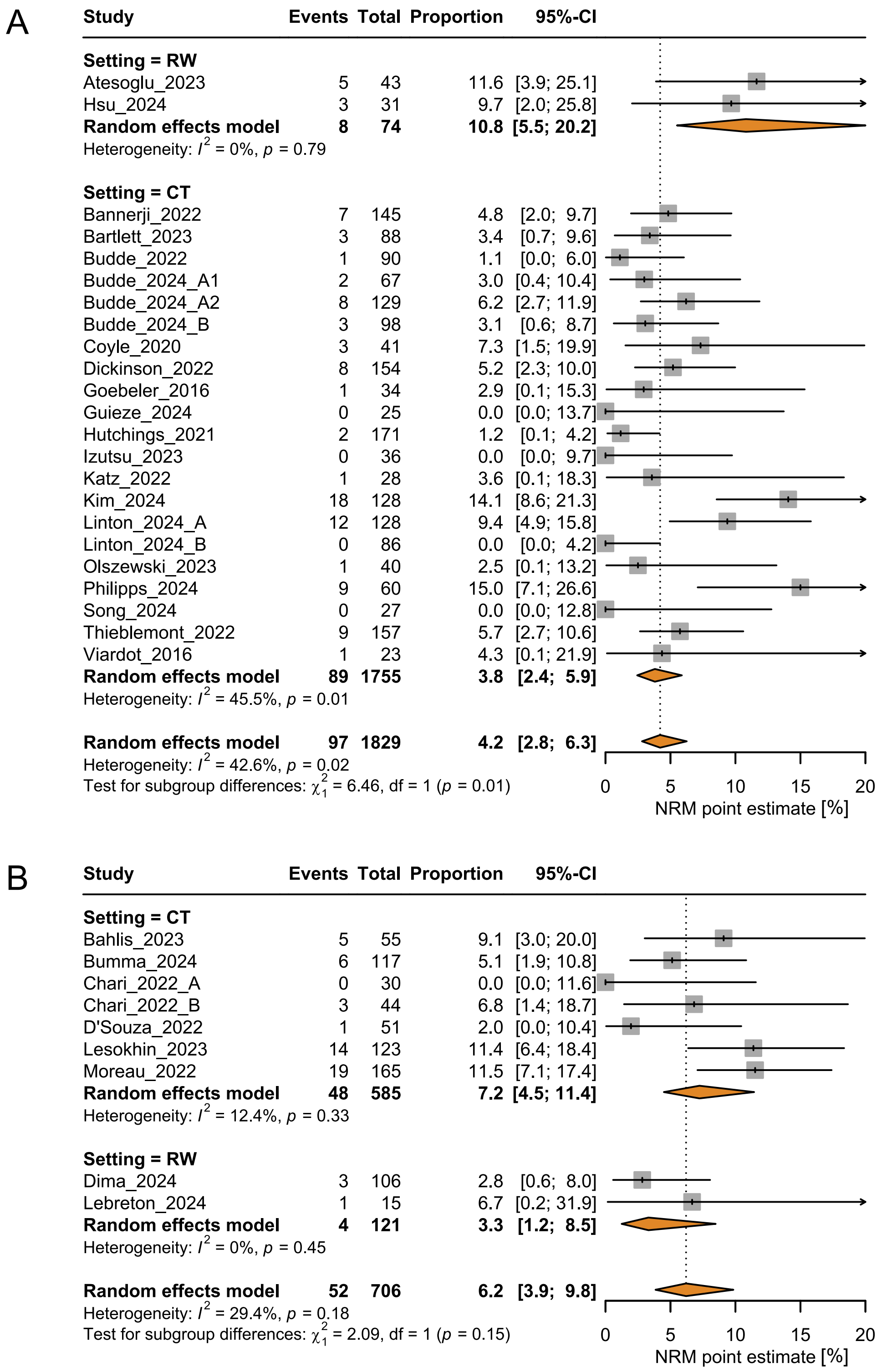

Figure S3. Subgroup analysis of NRM point estimates stratified by treatment setting. Forest plot illustrating NRM point estimates and 95% confidence intervals (95% CI) stratified by the treatment setting comparing real-world studies and clinical trials for B-Non-Hodgkin Lymphoma (A) and multiple myeloma (B). The random effects model results and heterogeneity measures are depicted for each treatment setting. CT = clinical trial, RW = real-world study.

A

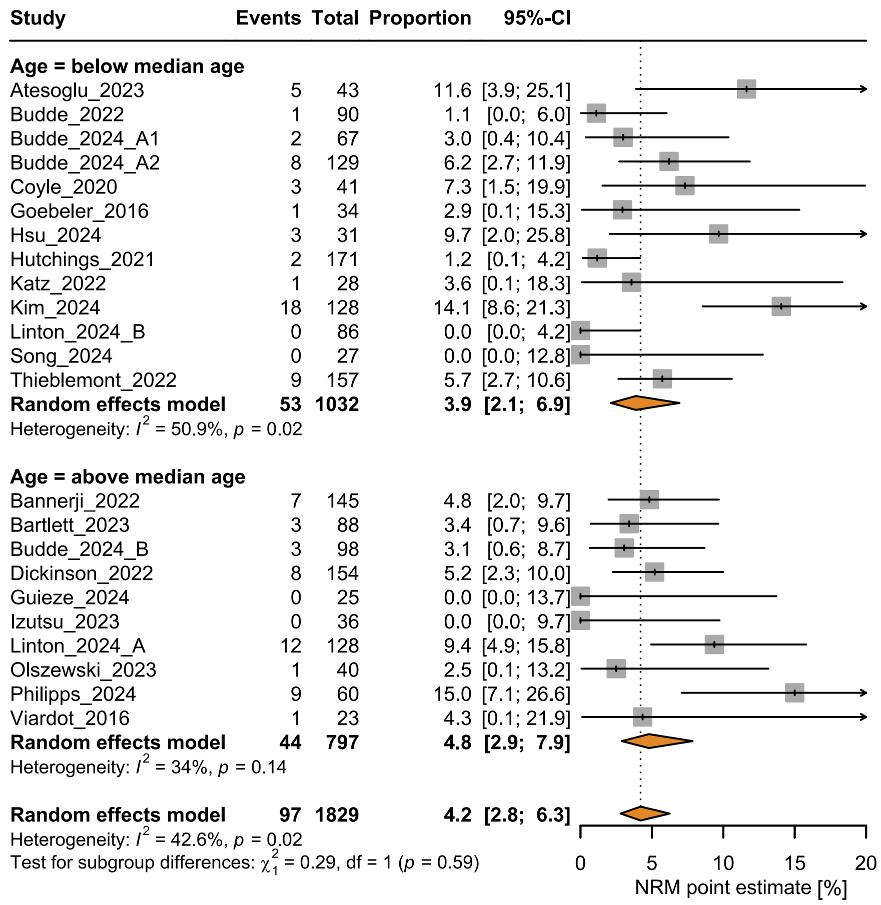

B

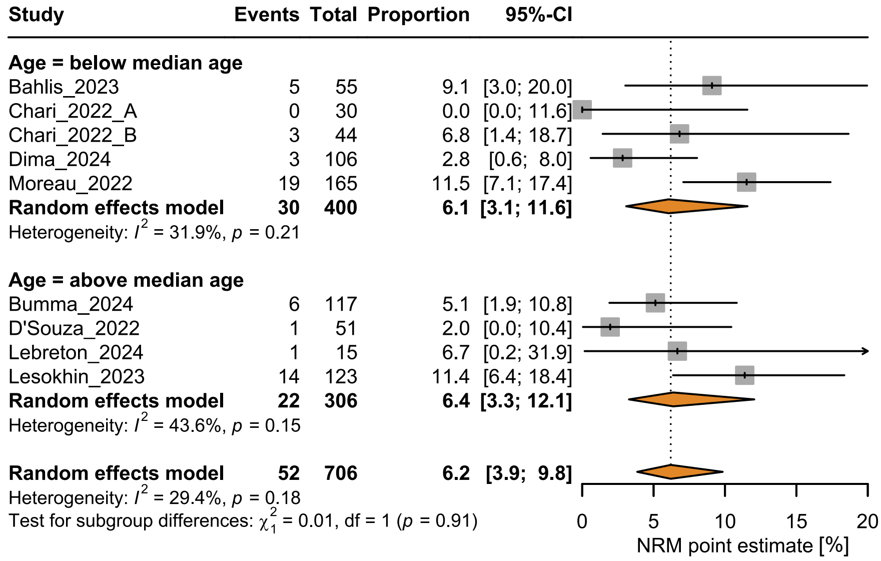

Figure S4. Subgroup analysis of NRM point estimates stratified by median age. Forest plot illustrating NRM point estimates and 95% confidence intervals (95% CI) stratified by the median age of each study cohort for B-Non-Hodgkin Lymphoma (A) and multiple myeloma (B). The random effects model results and heterogeneity measures are depicted for each disease entity. NRM = non-relapse mortality.

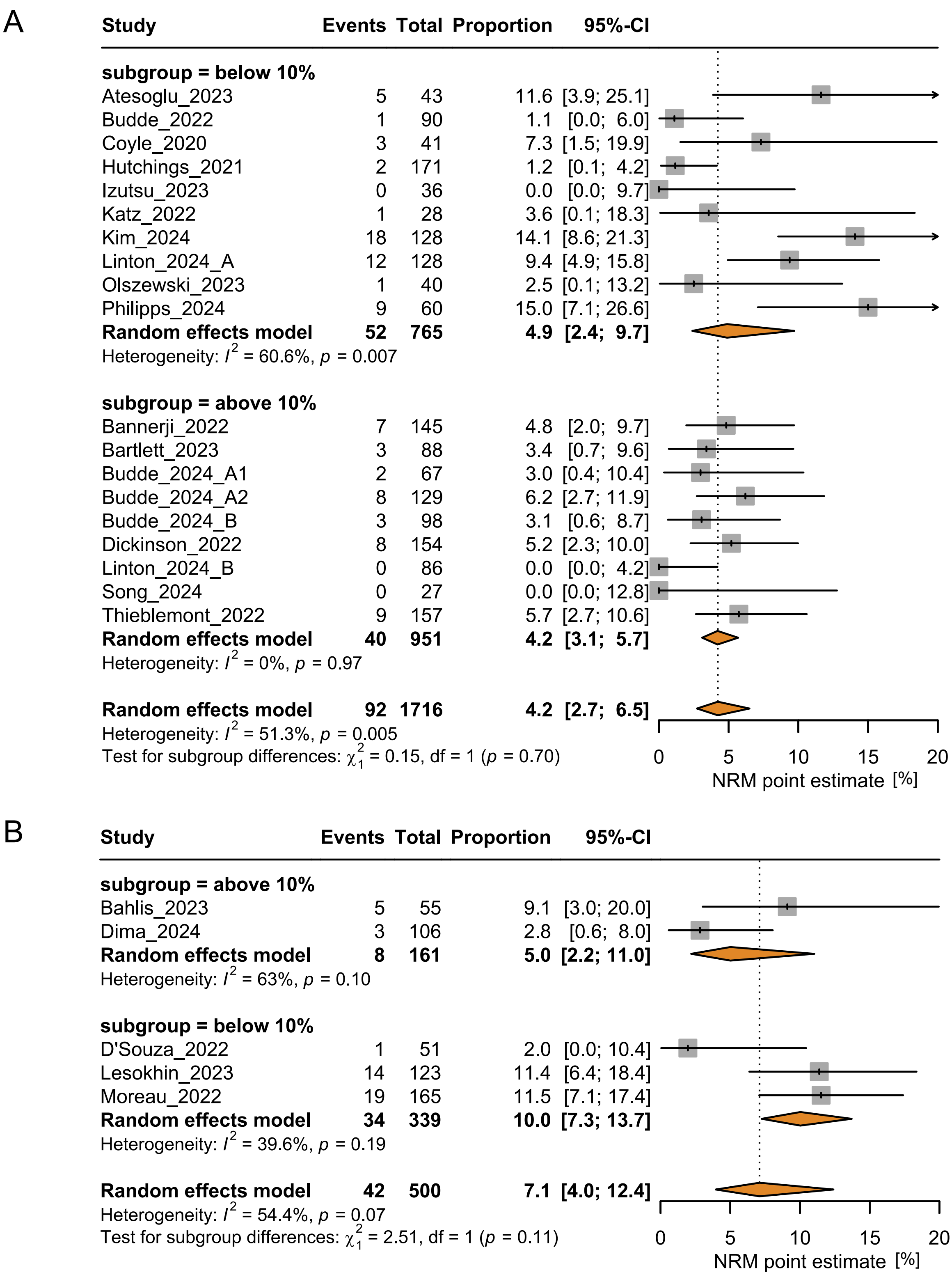

Figure S5. Subgroup analysis of NRM point estimates stratified by prior CAR-T exposure. Forest plot illustrating NRM point estimates and 95% confidence intervals (95% CI) stratified by prior CAR-T cell exposure of above or below 10% of the patients in each study cohort for B-Non-Hodgkin Lymphoma (A) and multiple myeloma (B). Only studies reporting prior CAR-T exposure were included in this analysis. The random effects model results and heterogeneity measures are depicted for cohorts with prior CAR-T exposure below and above 10%. Studies not reporting prior CAR-T exposure were excluded from analysis (Bumma et al, 2024, J Clin Oncol; Chari et al, 2022, N Engl J Med; Goebeler et al, 2016, J Clin Oncol; Guieze et al, 2024, Nat Commun; Viardot et al, 2016, Blood; Dima et al, 2024, Transplant Cell Ther; Hsu et al, 2024, Cancer). NRM = non-relapse mortality.

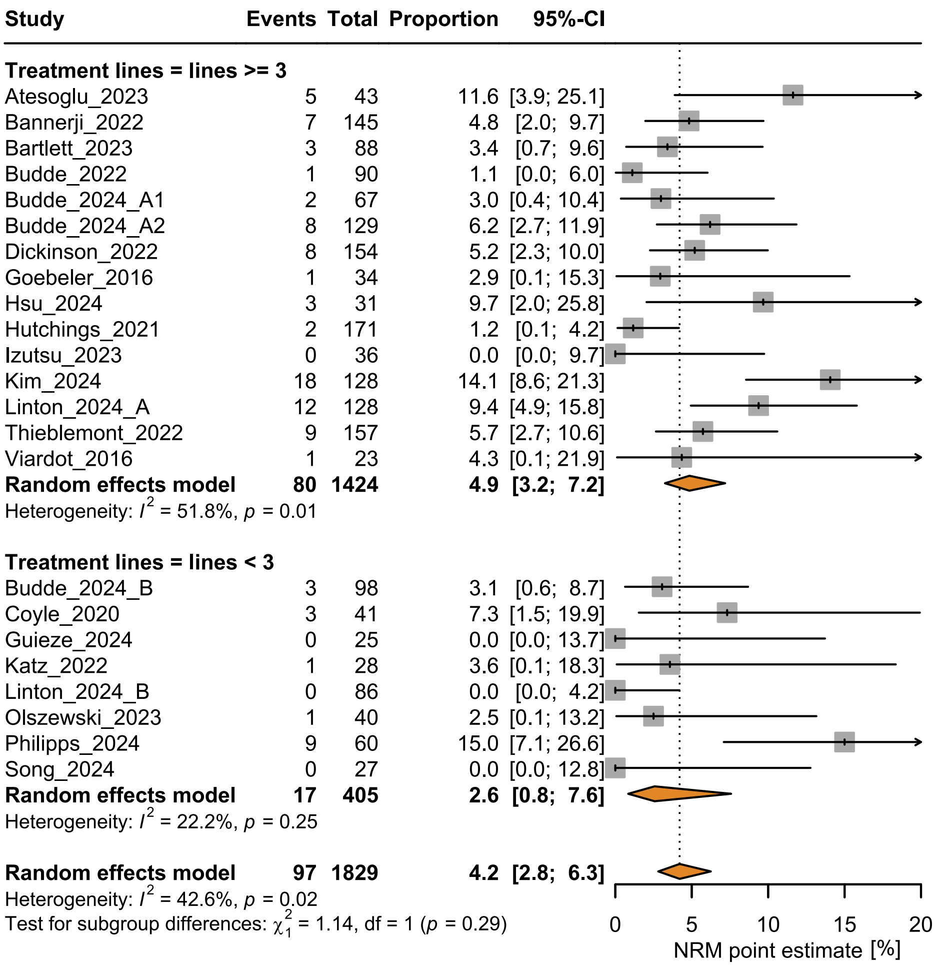

Figure S6. Subgroup analysis of NRM point estimates stratified by prior treatment lines within lymphoma cohorts. Forest plot illustrating NRM point estimates and 95% confidence intervals (95% CI) stratified by the median number of prior treatment lines among the lymphoma cohorts. The random effects model results and heterogeneity measures are depicted across cohorts with below and above three prior treatment lines. NRM = non-relapse mortality.

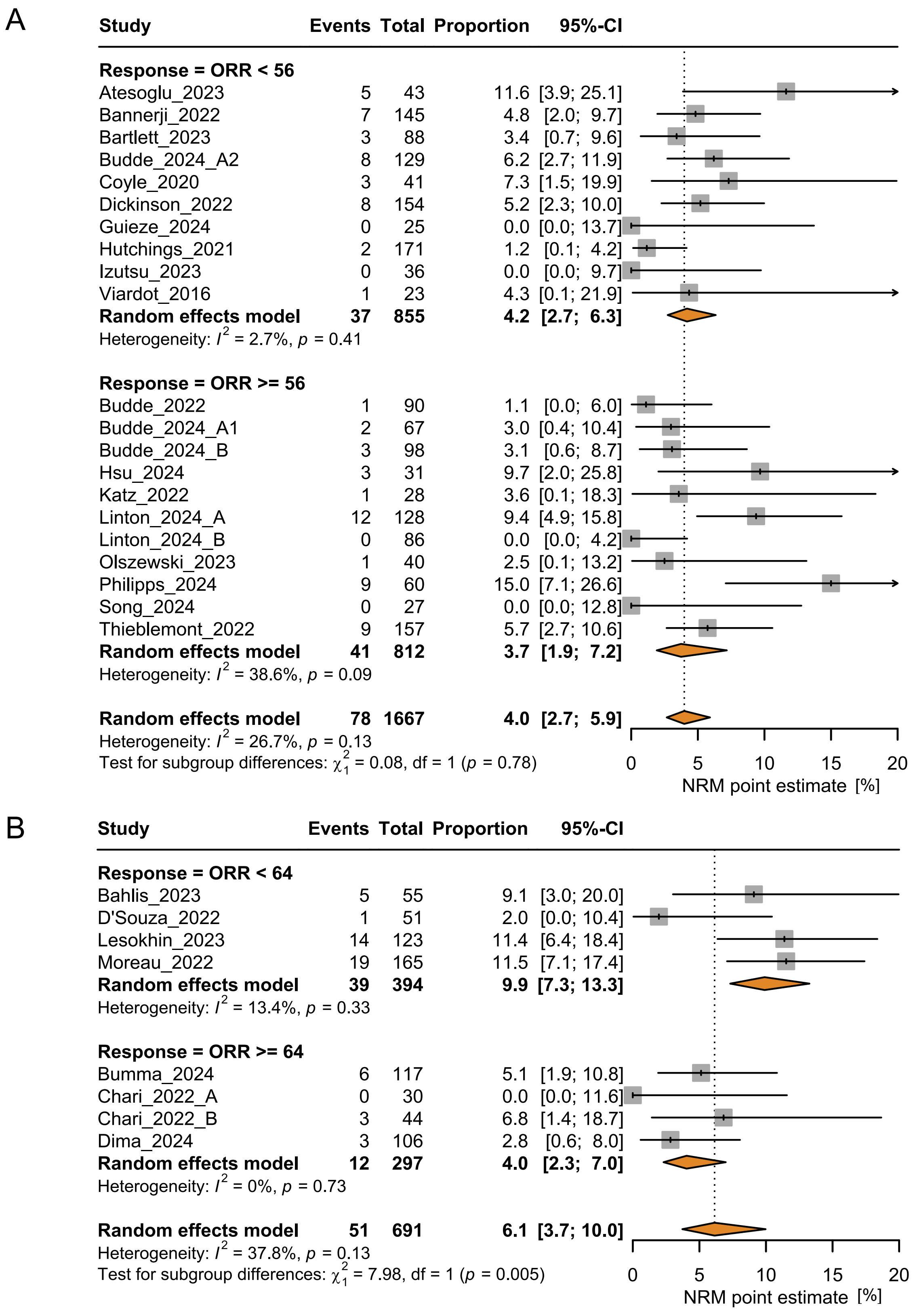

Figure S7. Subgroup analysis of NRM point estimates stratified by overall response rate. Forest plot illustrating non-relapse mortality (NRM) point estimates and 95% confidence intervals (95% CI) stratified by the median overall response rate (ORR) for B-Non-Hodgkin Lymphoma (A) and multiple myeloma (B). The random effects model results and heterogeneity measures are depicted for cohorts with an ORR below and above the median. Studies not reporting ORR were excluded from analysis (Goebeler et al, 2016, J Clin Oncol; Kim et al, 2024, Ann Oncol; Lebreton, 2024, Br J Haematol).

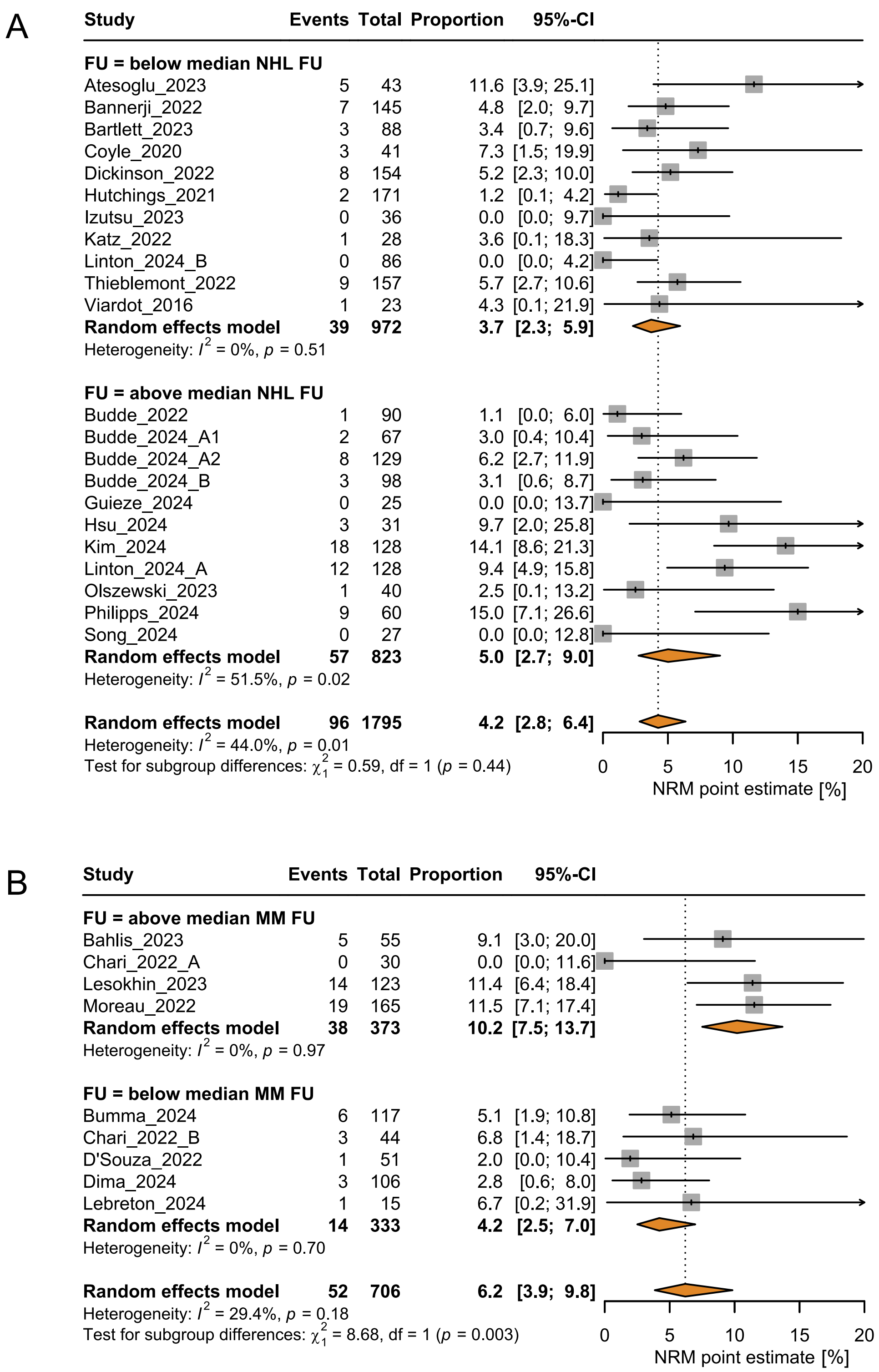

Figure S8. Subgroup analysis of NRM point estimates stratified by median follow-up time. Forest plot illustrating non-relapse mortality (NRM) point estimates and 95% confidence intervals (95% CI) stratified by the median follow-up (FU) time of each study cohort for B-Non-Hodgkin Lymphoma (A) and multiple myeloma (B). The random effects model results and heterogeneity measures are depicted for the cohorts below and above the median FU of 12 months. One study was excluded from analysis since no FU was reported (Goebeler et al, 2016, J Clin Oncol).

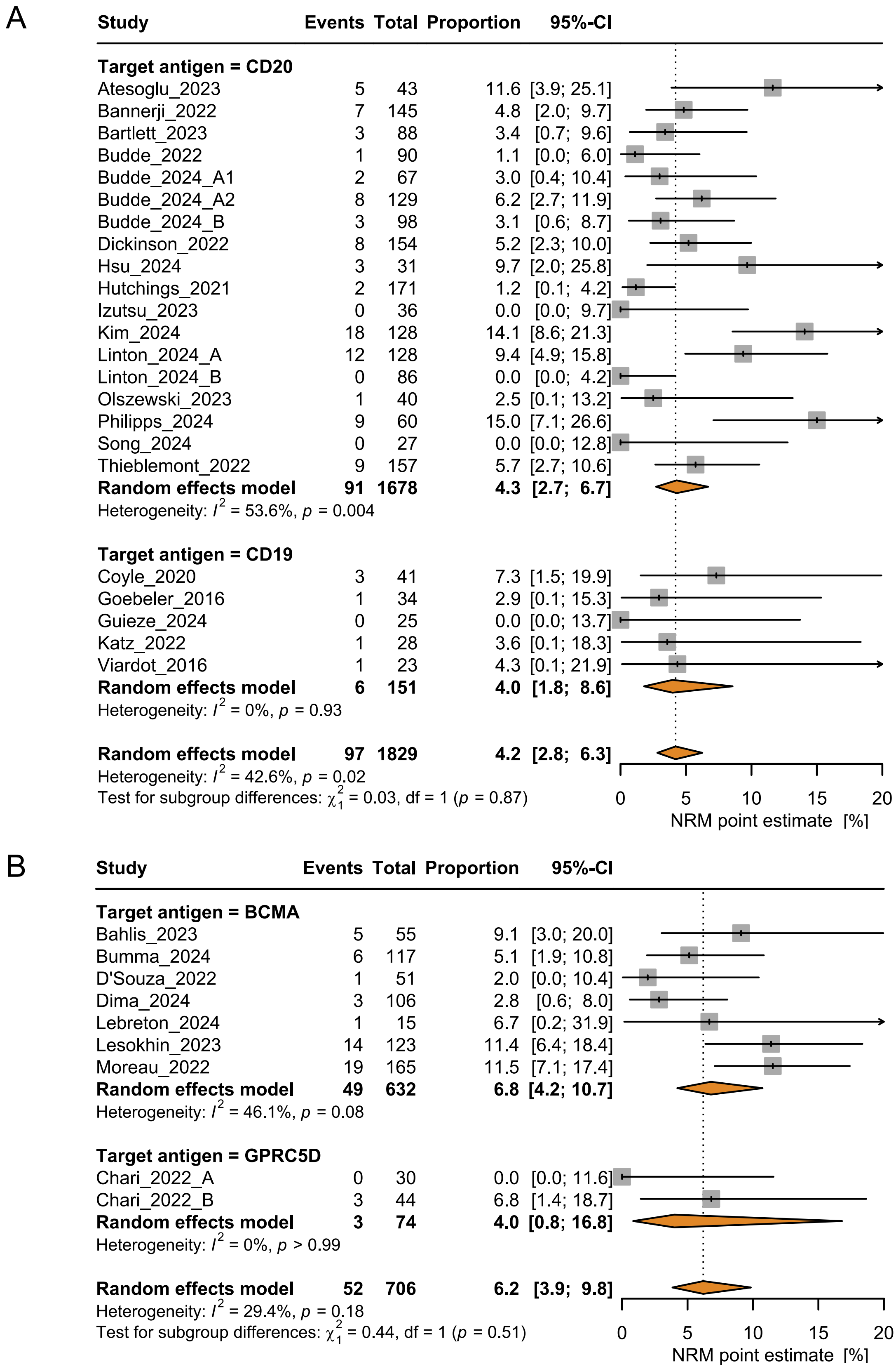

Figure S9. Subgroup analysis of NRM point estimates stratified by target antigen. Forest plot illustrating non-relapse mortality (NRM) point estimates and 95% confidence intervals (95% CI) stratified by target antigen for B-Non-Hodgkin Lymphoma (A) and multiple myeloma (B). The random effects model results and heterogeneity measures are depicted for the subcohorts.

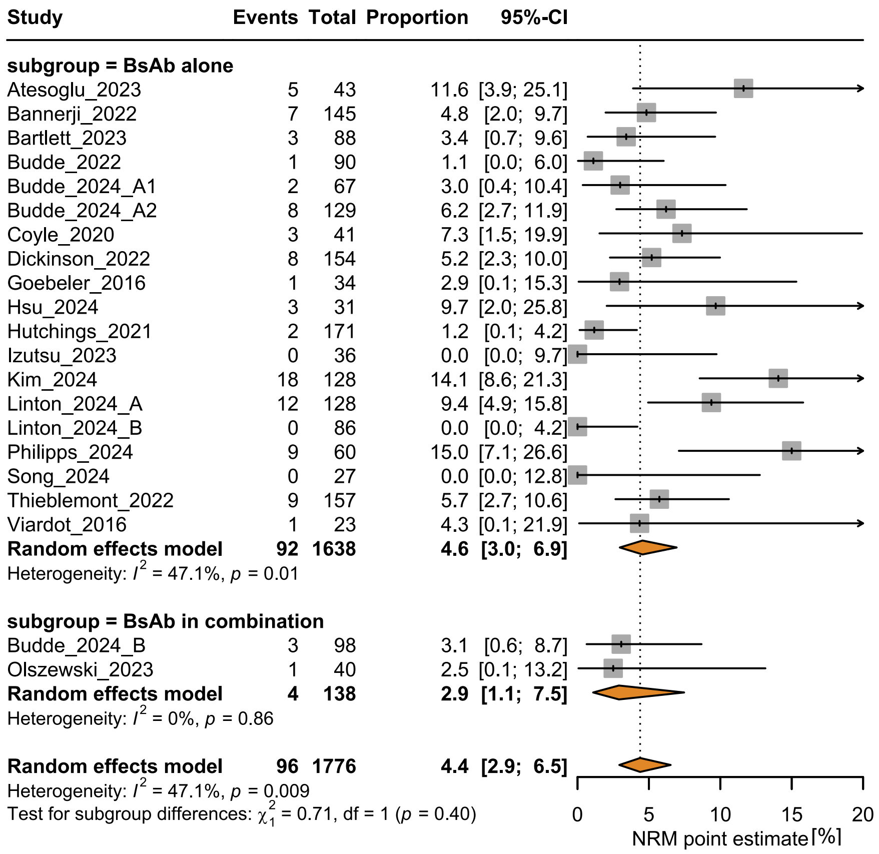

Figure S10. Subgroup analysis of NRM point estimates stratified by treatment mode in NHL cohorts. Forest plot illustrating non-relapse mortality (NRM) point estimates and 95% confidence intervals (95% CI) stratified by treatment mode of each study cohort for B-Non-Hodgkin Lymphoma (NHL). The random effects model results and heterogeneity measures are depicted for the cohorts treated with BsAb monotherapy vs. combination therapy. Two studies investigating BsAb therapy as consolidation therapy was excluded from analysis (Katz et al, 2022, Leuk Lymphoma and Guieze et al, 2024, Nat Commun).

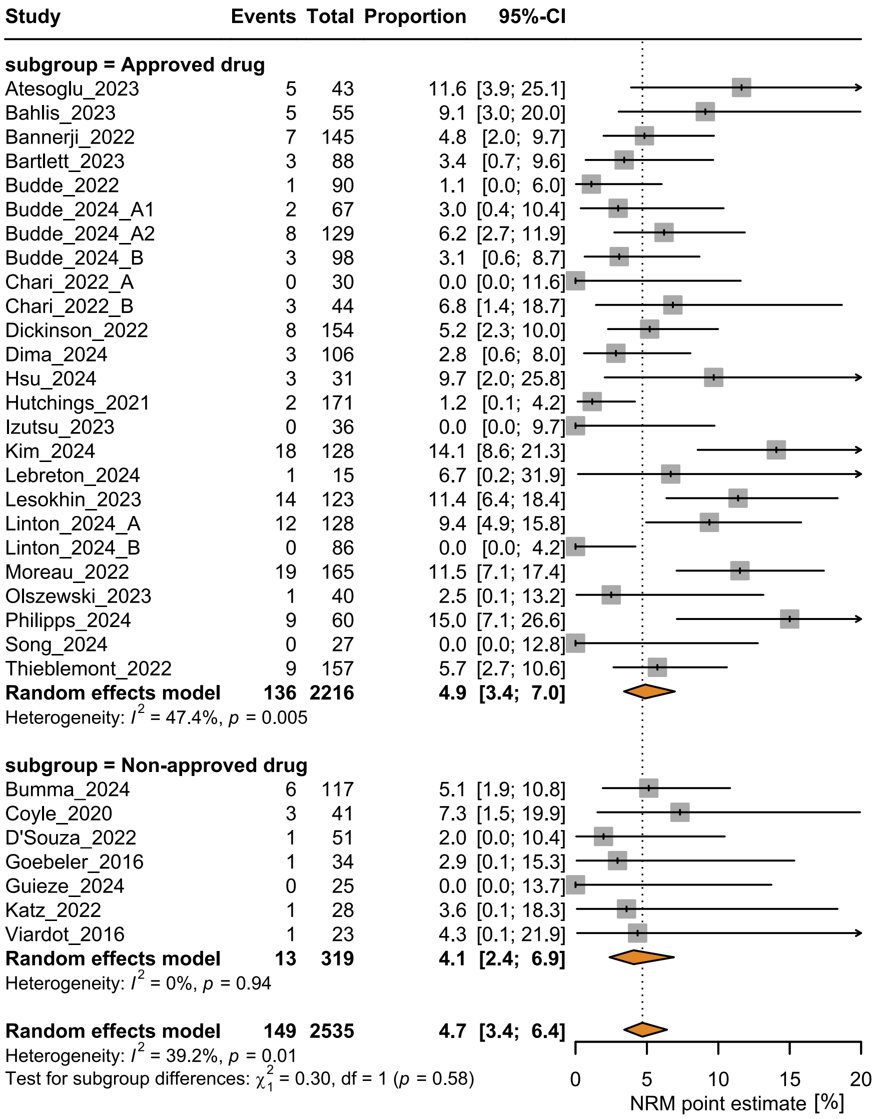

Figure S11. Subgroup analysis of NRM point estimates stratified by FDA and/or EMA approval. Forest plot illustrating non-relapse mortality (NRM) point estimates and 95% confidence intervals (95% CI) stratified by FDA/EMA approval for the investigated entity. The random effects model results and heterogeneity measures are depicted for the cohorts treated with products that are already approved by FDA or EMA and those not yet approved for the investigated entity by neither FDA nor EMA.

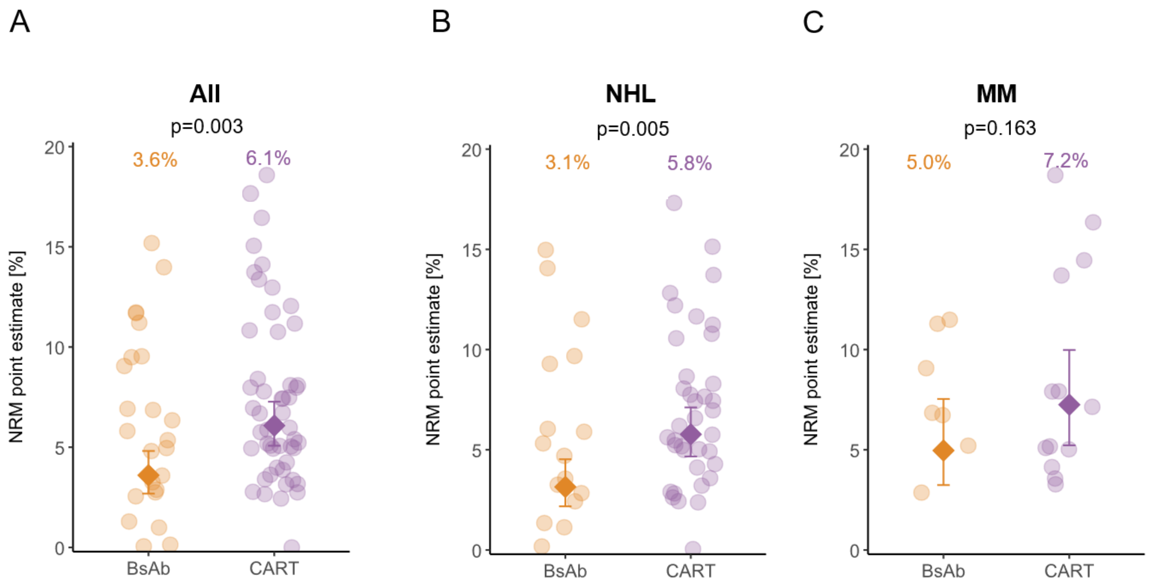

Figure S12. Comparative Analysis of NRM point estimates between BsAb and CAR T-cell therapy without COVID-19-related deaths. A-C Aggregated NRM point estimates and 95% confidence intervals are compared between BsAb- and CAR-T-treated patients across all disease entities (A) and specifically for NHL (B) and MM (C). NRM point estimates are calculated after exclusion of COVID-19-related deaths. P-value resembles test for subgroup heterogeneity. Abbreviations: NRM = non-relapse mortality; BsAb = bispecific antibody.

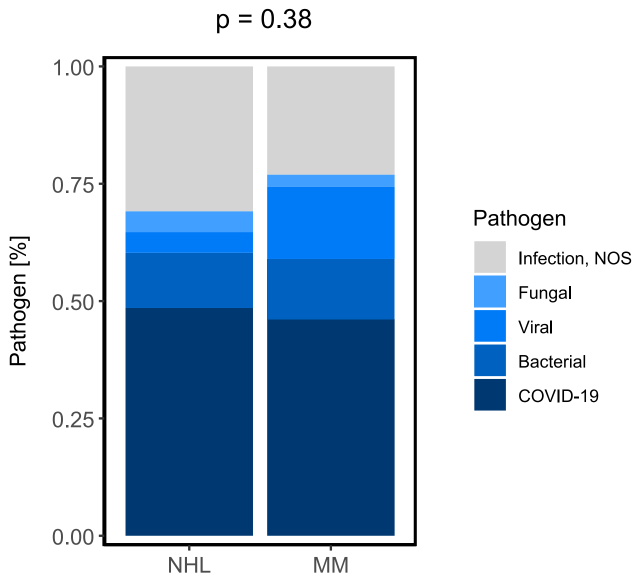

Figure S13. Distribution of pathogens responsible for fatal infections between patients with B-Non-Hodgkin-Lymphoma (NHL) and multiple myeloma (MM). Statistical significance was evaluated using the χ^2 distribution test. Abbreviations: NOS = not otherwise specified.

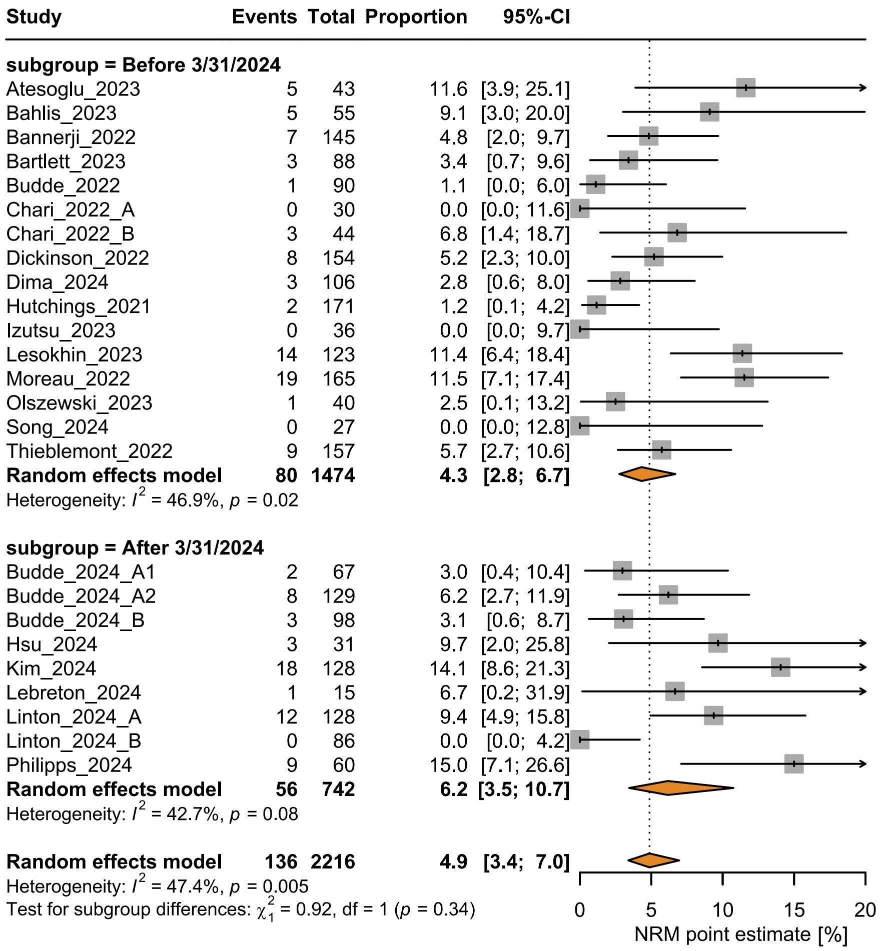

Figure S14. Subgroup analysis of NRM point estimates stratified by publication date. Forest plot illustrating non-relapse mortality (NRM) point estimates and 95% confidence intervals (95% CI) stratified by publication date. The random effects model results and heterogeneity measures are depicted for the records published before vs. after March 31st, 2024.

Table S1. Joanna-Brigg's Institute assessment of study bias.

First Author	Year	Criterion 1: Inclusion criteria	Criterion 2: measurement of mortality/ causes of death	Criterion 3: identification of mortality/ causes of death	Criterion 4: Consecutive Inclusion	Criterion 5: Complete Inclusion	Criterion 6: demographics (especially age, sex and ethnicity)	Criterion 7: Clinical information	Criterion 8: Follow-Up results
Linton	2024	yes	yes	yes	yes	yes	no	yes	yes
Budde	2024A	yes	yes	yes	yes	yes	no	yes	yes
Budde	2022	yes	yes	yes	yes	yes	yes	yes	yes
Kim	2024	yes	yes	yes	yes	yes	yes	yes	yes
Guieze	2024	yes	yes	yes	yes	yes	no	yes	yes
Coyle	2020	yes	yes	yes	yes	yes	yes	yes	yes
Viardot	2016	yes	yes	yes	yes	yes	no	yes	yes
Katz	2022	yes	yes	yes	yes	yes	no	yes	yes
Izutsu	2023	yes	yes	yes	yes	yes	yes	yes	yes
Thieblemont	2022	yes	yes	yes	yes	yes	yes	yes	yes
Hsu	2024	yes	yes	yes	yes	yes	no	yes	yes
Song	2024	yes	yes	yes	yes	yes	yes	yes	yes
Atsoglu	2023	yes	yes	yes	yes	yes	yes	yes	yes
Dickinson	2022	yes	yes	yes	yes	yes	yes	yes	yes
Budde	2024B	yes	yes	yes	yes	yes	no	yes	yes
Olszewski	2023	yes	yes	yes	yes	yes	no	yes	yes

Bartlett	2023	yes	yes	yes	yes	yes	no	yes	yes
Philipps	2024	yes	yes	yes	yes	yes	no	yes	yes
Goebeler	2016	yes	yes	yes	yes	yes	no	yes	no
Hutchings	2021	yes	yes	yes	yes	yes	no	yes	yes
Bannerji	2022	yes	yes	yes	yes	yes	yes	yes	yes
D'Souza	2022	yes	yes	yes	yes	yes	yes	yes	yes
Bahlis	2023	yes	yes	yes	yes	yes	yes	yes	yes
Lesokhin	2023	yes	yes	yes	yes	yes	yes	yes	no
Bumma	2024	yes	yes	yes	yes	yes	yes	yes	yes
Chari	2022	yes	yes	yes	yes	yes	yes	yes	yes
Lebreton	2024	no	yes	yes	no	no	no	yes	yes
Dima	2024	unclear	yes	yes	unclear	unclear	yes	yes	yes
Moreau	2022	yes	yes	yes	yes	yes	yes	yes	yes

Joanna-Brigg's Institute assessment of study bias (Munn et al. JBI Evid Synth. 2020 Oct;18(10):2127-2133). Criterion 9 (presenting sites'/clinics' demographics) was assessed within criterion 5 (demographics of the participants in the study). Criterion 10 (statistical analysis) was not applicable since the extracted data processed in this meta-analysis are not based on statistical tools in the original studies.

Table S2. Multivariable meta-regression analysis excluding COVID-related deaths.

	Studies	Patients	Estimate (95%CI)	p value
**Mechanism**				
- CAR-T [ref.]	45	6692		
- BsAb	23	1900	-0.35(-0.77;0.07)	0.10
**Entity**				
- IL [ref.]	8	764		
- LBCL	34	5697	-0.01 (-0.58;0.60)	0.98
- MCL	6	400	0.43 (-0.32;1.18)	0.25
- MM	20	1731	0.43 (-0.20;1.06)	0.18
**Prior treatment**				
- Treatment lines [median]	68	8592	-0.02(-0.18;0.14)	0.82
**Treatment setting**				
- CT [ref.]	36	3704		
- RW	32	4888	0.28 (-0.22;0.78)	0.27
**Follow-up**				
- Follow-up [years]	68	8592	0.24 (0.05;0.44)	**0.02**

Abbreviations: BsAb = Bispecific antibodies, IL = Indolent lymphoma, LBCL = Large B-cell lymphoma, MCL = Mantle cell lymphoma, MM = multiple myeloma, CT = clinical trial, RW = real-world study

Table S3. Causes of death classified as "others".

Case	Author	Year	Cause of Death
1	Bahlis	2023	Sudden death
2	Lesokhin	2023	Failure to thrive
3	Dickinson	2022	Delirium
4	Bannerji	2022	Gastric perforation
5	Bannerji	2022	Tumor lysis syndrome
6	Thieblemont	2022	Loss of consciousness
7	Thieblemont	2022	General health deterioration
8	Coyle	2020	Pancytopenia

Table S4. Extended characteristics of included BsAb records.

Entity	First Author	Year	Cohort	Product	Setting	Time Frame of Inclusion	Patients receiving immunoglobulins [%]
IL	Linton	2024	A	Epcoritamab	II	06/20-04/23	NR
	Linton	2024	B	Epcoritamab	II	10/22-01/24	NR
	Budde	2024	A1	Mosunetuzumab	I/II	09/15-05/19	NR
	Budde	2022		Mosunetuzumab	II	05/19-09/20	NR
	Kim	2024		Odronextamab	II	12/19-07/22	NR
LBCL	Guieze	2024		Blinatumomab	II	07/19-07/21	NR
	Coyle	2020		Blinatumomab	II	01/17-01/18	NR
	Viardot	2016		Blinatumomab	II	08/12-07/14	NR
	Katz	2022		Blinatumomab	II	03/17-05/18	NR
	Izutsu	2023		Epcoritamab	II	01/21-01/22	NR
	Thieblemont	2022		Epcoritamab	I/II	06/20-01/22	NR
	Hsu	2024		Glofitamab	RW	01/21-10/22	NR
	Song	2024		Glofitamab	I	NR/NR-12/22	NR
	Atesoglu	2023		Glofitamab	RW	03/21-09/22	NR
	Dickinson	2022		Glofitamab	II	01/20-09/21	NR
	Budde	2024	A2	Mosunetuzumab	I/II	09/15-05/19	NR
	Budde	2024	B	Mosunetuzumab	II	09/18-02/22	NR
	Olszewski	2023		Mosunetuzumab	II	11/19-07/20	NR
	Bartlett	2023		Mosunetuzumab	II	04/19-02/20	NR
MCL	Philipps	2024		Glofitamab	I/II	NR/NR-09/23	NR
NHL	Goebeler	2016		Blinatumomab	I	06/04-07/11	NR
	Hutchings	2021		Glofitamab	I	NR/NR-08/20	NR
	Bannerji	2022		Odronextamab	I	02/15-09/21	NR

	D'Souza	2022		ABBV-383 (Etentamig)	I	NR/NR-01/22	NR
	Bahlis	2023		Elranatamab	I	11/17-04/21	32.7
	Lesokhin	2023		Elranatamab	II	02/21-01/22	43.1
	Bumma	2024		Linvoseltamab	I/II	01/19-10/22	NR
MM	Chari	2022	A	Talquetamab	I	01/18-11/21	NR
	Chari	2022	B	Talquetamab	I	01/18-11/21	NR
	Lebreton	2024		Teclistamab	RW	11/22-10/23	53.3
	Dima	2024		Teclistamab	RW	08/22-08/23	41.5
	Moreau	2022		Teclistamab	I/II	03/20-08/21	39.4

Abbreviations: IL = Indolent lymphoma, LBCL = Large B-cell lymphoma, MCL = Mantle cell lymphoma, MM = multiple myeloma, CT = clinical trial, RW = real-world study, NR = not reported.

Table S5. Sensitivity analyses.

	**Reported results:**<br><br>Random effects model including all studies	**Sensitivity analysis 1:**<br><br>Fixed effects model including all studies	**Sensitivity analysis 2:**<br><br>Random effects model excluding COVID-related deaths	**Sensitivity analysis 3:**<br><br>Random effects model excluding studies with dose escalation cohorts
**Overall NRM BsAb**	4.7 (3.4-6.4)	5.9 (5.0-6.9)	3.5 (2.6-4.6)	5.0 (3.5-7.0)
**Entity**				
- NHL	4.2 (2.8-6.3)	5.3 (4.4-6.4)	3.2 (2.3-4.5)	4.4 (2.7-7.0)
- MM	6.2 (3.9-9.8)	7.4 (5.7-9.5)	4.4 (2.7-7.1)	NA
**Overall NRM CAR-T**	6.8 (5.8-8.0)	7.5 (7.0-8.1)	NA	NA

Abbreviations: NRM = Non-relapse mortality [%], BsAb = Bispecific antibodies.

Table S6. Causes of death classified as "infections".

Case	First Author	Year	Cohort	Entity	Cause of Death
1	Atesoglu	2023		NHL	COVID-19
2	Atesoglu	2023		NHL	COVID-19
3	Atesoglu	2023		NHL	Sepsis from gram-negative bacteria
4	Bahlis	2023		MM	COVID-19
5	Bahlis	2023		MM	COVID-19
6	Bahlis	2023		MM	Septic shock
7	Bahlis	2023		MM	Adenoviral infection
8	Bannerji	2022		NHL	Pneumonia
9	Bannerji	2022		NHL	Pneumocystis jirovecii pneumonia
10	Bannerji	2022		NHL	COVID-19
11	Bartlett	2023		NHL	Pneumonia
12	Bartlett	2023		NHL	Sepsis
13	Bartlett	2023		NHL	Cholangitis in neutropenic fever
14	Budde	2024	A1	NHL	Pneumonia
15	Budde	2024	A2	NHL	Sepsis
16	Budde	2024	A2	NHL	Bacteremia due to bowel perforation
17	Budde	2024	A2	NHL	Sepsis
18	Budde	2024	B	NHL	COVID-19
19	Budde	2024	B	NHL	COVID-19
20	Budde	2024	B	NHL	Pneumonia
21	Bumma	2024		MM	Pneumocystis jirovecii pneumonia
22	Bumma	2024		MM	Progressive multifocal leukoencephalopathy
23	Bumma	2024		MM	Sepsis from P. aeruginosa
24	Bumma	2024		MM	Infection
25	Bumma	2024		MM	Infection
26	Chari	2022	B	MM	Sepsis
27	Coyle	2020		NHL	Sepsis
28	Dickinson	2022		NHL	COVID-19
29	Dickinson	2022		NHL	COVID-19
30	Dickinson	2022		NHL	COVID-19
31	Dickinson	2022		NHL	COVID-19
32	Dickinson	2022		NHL	COVID-19
33	Dickinson	2022		NHL	Sepsis
34	Dickinson	2022		NHL	Sepsis
35	Dima	2024		MM	COVID-19
36	Dima	2024		MM	Pneumonia from rhino/adenovirus
37	Dima	2024		MM	Sepsis
38	D'Souza	2022		MM	COVID-19
39	Goebeler	2016		NHL	Pneumocystis jirovecii pneumonia
40	Hsu	2024		NHL	COVID-19
41	Hsu	2024		NHL	Sepsis from bacteria
42	Hutchings	2021		NHL	Septic shock
43	Katz	2022		NHL	infection
44	Kim	2024		NHL	Pneumonia
45	Kim	2024		NHL	Pneumonia

46	Kim	2024		NHL	Pneumonia
47	Kim	2024		NHL	Sepsis
48	Kim	2024		NHL	Systemic mycosis
49	Kim	2024		NHL	Progressive multifocal leukoencephalopathy
50	Kim	2024		NHL	Pneumonia from P. aeruginosa
51	Kim	2024		NHL	Sepsis from E. coli
52	Kim	2024		NHL	Progressive multifocal leukoencephalopathy
53	Kim	2024		NHL	COVID-19
54	Kim	2024		NHL	COVID-19
55	Kim	2024		NHL	COVID-19
56	Kim	2024		NHL	COVID-19
57	Kim	2024		NHL	COVID-19
58	Kim	2024		NHL	COVID-19
59	Kim	2024		NHL	COVID-19
60	Kim	2024		NHL	COVID-19
61	Lebreton	2024		MM	Bacterial infection
62	Lesokhin	2023		MM	Adenoviral hepatitis
63	Lesokhin	2023		MM	Pneumonia from adenovirus
64	Lesokhin	2023		MM	Pneumonia from P. aeruginosa
65	Lesokhin	2023		MM	COVID-19
66	Lesokhin	2023		MM	COVID-19
67	Lesokhin	2023		MM	Infection
68	Lesokhin	2023		MM	Infection
69	Lesokhin	2023		MM	Infection
70	Linton	2024	A	NHL	COVID-19
71	Linton	2024	A	NHL	COVID-19
72	Linton	2024	A	NHL	COVID-19
73	Linton	2024	A	NHL	COVID-19
74	Linton	2024	A	NHL	COVID-19
75	Linton	2024	A	NHL	COVID-19
76	Linton	2024	A	NHL	Sepsis from P. aeruginosa
77	Linton	2024	A	NHL	Organizing pneumonia
78	Linton	2024	A	NHL	Pneumonia
79	Moreau	2022		MM	COVID-19
80	Moreau	2022		MM	COVID-19
81	Moreau	2022		MM	COVID-19
82	Moreau	2022		MM	COVID-19
83	Moreau	2022		MM	COVID-19
84	Moreau	2022		MM	COVID-19
85	Moreau	2022		MM	COVID-19
86	Moreau	2022		MM	COVID-19
87	Moreau	2022		MM	COVID-19
88	Moreau	2022		MM	COVID-19
89	Moreau	2022		MM	Pneumonia
90	Moreau	2022		MM	Pneumonia from P. aeruginosa
91	Moreau	2022		MM	COVID-19
92	Moreau	2022		MM	COVID-19

93	Moreau	2022	MM	Pneumonia from streptococci
94	Moreau	2022	MM	Progressive multifocal leukoencephalopathy
95	Olszewski	2023	NHL	Pneumonia
96	Philipps	2024	NHL	COVID-19
97	Philipps	2024	NHL	COVID-19
98	Philipps	2024	NHL	COVID-19
99	Philipps	2024	NHL	COVID-19
100	Philipps	2024	NHL	COVID-19
101	Philipps	2024	NHL	COVID-19
102	Philipps	2024	NHL	Pneumonia
103	Philipps	2024	NHL	Septic shock
104	Thieblemont	2022	NHL	COVID-19
105	Thieblemont	2022	NHL	COVID-19
106	Thieblemont	2022	NHL	Progressive multifocal leukoencephalopathy
107	Viardot	2016	NHL	Pneumonia

Further Supplemental Material:

Study Protocol
