# Supplementary material for: Non-relapse mortality with bispecific antibodies: A systematic review and meta-analysis in lymphoma and multiple myeloma
Source: Mol Ther. 2025 Mar 31;33(7):3163–76. doi: 10.1016/j.ymthe.2025.03.048 (PMC12266037; doi:10.1016/j.ymthe.2025.03.048)
Supplement: Document S2. Article plus supplemental information [file mmc3.pdf]

Molecular Therapy
Original Article

Non-relapse mortality with bispecific antibodies: A systematic review and meta-analysis in lymphoma and multiple myeloma

Tobias Tix,[1] Mohammad Alhomoud,[2] Roni Shouval,[2,3] Gloria Iacoboni,[4,5] Edward R. Scheffer Cliff,[6,7,8] Doris K. Hansen,[9] Saad Z. Usmani,[3,10] Gilles Salles,[3,11] Miguel-Angel Perales,[2,3] David M. Cordas dos Santos,[1,6,7,12,14] and Kai Rejeski[1,2,13,14]

[1]Department of Medicine III – Hematology/Oncology, LMU University Hospital, LMU Munich, Munich, Germany; [2]Adult Bone Marrow Transplantation Service, Department of Medicine, Memorial Sloan Kettering Cancer Center, New York, NY, USA; [3]Department of Medicine, Weill Cornell Medical College, New York, NY, USA; [4]Department of Hematology, University Hospital Vall d'Hebron, Vall d'Hebron Institute of Oncology (VHIO), Barcelona, Spain; [5]Department of Medicine, Universitat Autònoma de Barcelona, Bellaterra, Spain; [6]Department of Medical Oncology, Dana-Farber Cancer Institute, Boston, MA, USA; [7]Harvard Medical School, Boston, MA, USA; [8]Department of Clinical Haematology, Peter MacCallum Cancer Centre and Royal Melbourne Hospital, Melbourne, Australia; [9]Department of Blood and Marrow Transplant and Cellular Immunotherapy, Moffitt Cancer Center, Tampa, FL, USA; [10]Myeloma Service, Department of Medicine, Memorial Sloan Kettering Cancer Center, New York, NY, USA; [11]Lymphoma Service, Department of Medicine, Memorial Sloan Kettering Cancer Center, New York, NY, USA; [12]Broad Institute of Massachusetts Institute of Technology (MIT) and Harvard University, Cambridge, MA, USA; [13]German Cancer Consortium (DKTK), Partner Site Munich, a partnership between the DKFZ Heidelberg and LMU University Hospital, Munich, Germany

Bispecific antibodies (BsAb) are associated with distinct immune-related toxicities that impact morbidity and mortality. This systematic review and meta-analysis examined non-relapse mortality (NRM) with BsAb therapy in B-cell non-Hodgkin lymphoma (NHL) and multiple myeloma (MM). A PubMed and Embase search up to October 2024 identified 29 studies (21 NHL, 8 MM) involving 2,535 patients. The overall NRM point estimate was 4.7% (95% confidence interval [CI] 3.4%–6.4%), with a median follow-up of 12.0 months. We noted no significant difference in NRM across disease entities (NHL: 4.2%, MM: 6.2%, $p = 0.22$). In NHL, prespecified subgroup analyses revealed increased NRM in real-world studies compared to clinical trials. For MM, an association between NRM and higher response rates and longer follow-up was noted. Meta-regression comparing BsAb and CAR-T therapies ($n = 8,592$) showed no significant NRM difference when accounting for key study-level confounders ($p = 0.96$). Overall, infections were the leading cause of NRM, accounting for 71.8% of non-relapse deaths. Of the infection-related deaths, 48% were attributed to COVID-19. In a pre-specified sensitivity analysis excluding COVID-19 fatalities, the overall NRM estimate was 3.5% (95% CI 2.6%–4.6%). Taken together, these results provide a benchmark for the estimated NRM with BsAb therapy and highlight the paramount importance of infection reporting, prevention, and mitigation.

INTRODUCTION

Bispecific antibodies (BsAb) represent a modern immunotherapeutic approach, linking CD3 on T-cells to tumor-associated antigens on cancer cells, thereby enabling direct targeting of malignant cells without the need for *ex vivo* cell manipulation.[1,2] While the most extensively studied BsAb targeting CD19, CD20, GPRC5D, and BCMA have so far been used to treat patients with relapsed or refractory lymphoid and plasma cell malignancies,[3,4] multiple studies are currently investigating their efficacy and safety in earlier lines of treatment[5] and for non-malignant conditions like autoimmune diseases.[6] BsAb have emerged as both an alternative and an adjunct to chimeric antigen receptor (CAR) T-cell therapies. They offer the advantages of being off the shelf, with easier administration and the potential for repeated dosing in settings where treatment can be paused upon response and resumed upon relapse.[7–9]

While BsAb and CART-cell therapies share T-cell activation and cytotoxicity mechanisms, they differ in their adverse event profiles. Both approaches are associated with immune-related toxicities such as cytokine release syndrome (CRS) and immune effector cell-associated neurotoxicity syndrome (ICANS). However, the incidence and severity of these toxicities tend to be lower with BsAb compared to CAR T-cell therapies, possibly due to the absence of the large-scale T-cell expansion characteristic of CAR T-cell therapy.[10–12] However, infectious complications are a significant and concerning toxicity associated

Received 28 December 2024; accepted 26 March 2025;
https://doi.org/10.1016/j.ymthe.2025.03.048.

[14]Senior author

Correspondence: Dr. Kai Rejeski, MD, MHBA, Principal Investigator, Laboratory of Precision Immunotherapy, Department of Medicine III, LMU University Hospital, LMU Munich, Munich, Germany.
E-mail: kai.rejeski@med.uni-muenchen.de

Published by Elsevier Inc. on behalf of The American Society of Gene and Cell Therapy.
This is an open access article under the CC BY license (http://creativecommons.org/licenses/by/4.0/).

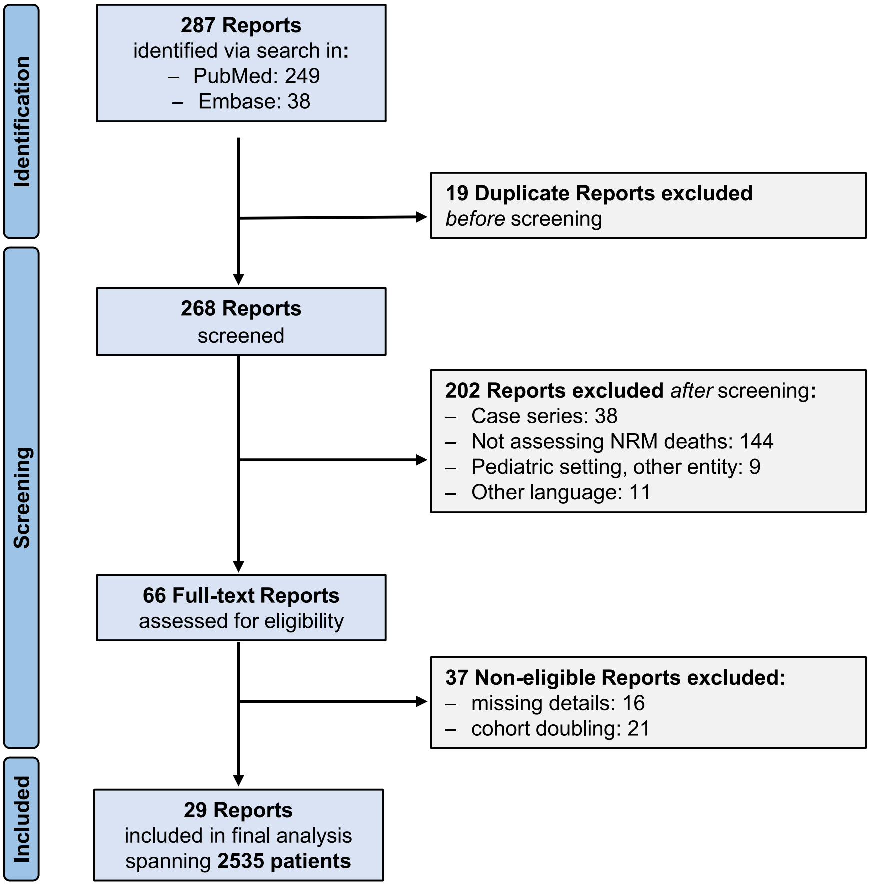

Figure 1. Study retrieval and identification for meta-analysis
Flowchart illustrating the inclusion and exclusion process for the systematic review and meta-analysis of non-relapse mortality (NRM) associated with BsAb therapy, conducted in accordance with PRISMA guidelines.

with BsAb therapy.[13,14] These infections often arise from on-target/off-tumor effects, such as B-cell aplasia and hypogammaglobulinemia, leading to sustained immunosuppression and increased infection susceptibility.[15,16] Additionally, factors such as neutropenia, T-cell exhaustion, prior chemotherapy, and the underlying malignancy further contribute to this vulnerability.[11,13,17]

Given the cumulative burden of these potentially fatal toxicities, understanding non-relapse mortality (NRM), defined as death not preceded by disease recurrence or progression, associated with BsAb therapy is essential. While NRM is a well-established metric in allogeneic hematopoietic cell transplantation (HCT) and has drawn growing attention in the context of CAR T-cell therapies,[18–20] data on NRM associated with BsAb therapies remain limited.

This systematic review and meta-analysis addresses this gap by analyzing the incidence and causes of NRM associated with BsAb therapy across a spectrum of advanced B-cell malignancies. Additionally, we leverage the insights gained from our previous analysis of NRM with CAR T-cell therapy,[18] to provide a comprehensive perspective on immunotherapy-related mortality risks across treatment modalities.

RESULTS

Study cohort

We screened a total of 287 studies for reports of non-relapse deaths in patients treated with BsAb therapies. Of these, 66 full-text articles were assessed for NRM and causes of death, and 29 articles met the criteria for further analysis (Figure 1). These included 25 reports on clinical trials (CTs),[9,21–44] encompassing a total of 2,340 patients (phase 1: 7, phase 1/2: 5, phase 2: 13), and 4 real-world studies (RW)[45–48] involving 195 patients (Table 1). Two CTs were subdivided by dose-specific[27,38] and one CT by disease-specific cohorts,[9] resulting in 32 distinct study cohorts for final evaluation.

The most common entity was large B-cell lymphoma (LBCL, 920 patients), followed by multiple myeloma (MM, 706 patients), indolent lymphoma (IL, 499 patients), and mantle cell lymphoma (MCL, 60 patients). Three studies did not specify lymphoma subtypes (350 patients).[22,31,33] For non-Hodgkin lymphoma (NHL), the distribution of BsAb products was 6 glofitamab, 6 mosunetuzumab, 5 blinatumomab, 4 epcoritamab, and 2 odronextamab. Among the MM cohorts, 3 were treated with teclistamab, 2 with talquetamab, 2 with elranatamab, and 1 each with linvoseltamab and ABBV-383 (etentamig). Follow-up duration ranged from 1.0 to 40.8 months across studies.

NRM estimates did not vary significantly across disease entities and BsAb products

Across all patients, the overall NRM point estimate was 4.7% (95% confidence interval [CI] 3.4%–6.4%; Figure 2) after a median follow-up of 12.0 months. Incorporating the median follow-up per study, we observed 0.043 NRM events per patient-year (Figure S1). Study heterogeneity was moderate (I^2 = 39.2%; Figure 2).[49] While we identified a risk of publication bias by funnel plot analysis (Egger's test $p < 0.001$; Figure S2), we did not detect a risk of study bias, except incomplete reporting of ethnicity (Table S1). Since underlying tumor biology and previous treatments may affect non-relapse deaths, we next investigated associations between tumor entities and NRM point estimates.

While NRM was numerically lower with NHL (4.2%, 95% CI 2.8%–6.3%), we did not detect a significant difference compared to MM (6.2%, 95% CI 3.9%–9.8%, p = 0.22; Figure 2). Furthermore, the

Table 1. Characteristics of included records

Entity	First author	Year	Cohort	Product	Setting	Cohort size	Non-relapse deaths	NRM point estimate, %	Follow- up, months	Age, y, median	Prior treatment lines, median	Prior CAR-Ts, %	ORR, %	Treatment mode
IL	Linton	2024	A	epcoritamab	2	128	12	9.38	17.4	65	3	5	82.0	exp
	Linton	2024	B	epcoritamab	2	86	0	0.00	5.7	64	2	7	86.0	exp
	Budde	2024	A1	mosunetuzumab	1/2	67	2	2.99	39.6	60	3	6	65.7	esc+exp
	Budde	2022		mosunetuzumab	2	90	1	1.11	18.3	60	3	3	80.0	NA
	Kim	2024		odronextamab	2	128	18	14.06	20.1	61	3	0	NR	NA
LBCL	Guieze	2024		blinatumomab	2	25	0	0.00	23.2	66	1	NR	36.0	cons
	Coyle	2020		blinatumomab	2	41	3	7.32	4.9	56	2	0	37.0	NA
	Viardot	2016		blinatumomab	2	23	1	4.35	11.7	66	3	NR	43.0	NA
	Katz	2022		blinatumomab	2	28	1	3.57	12	64	0	0	89.3	cons
	Izutsu	2023		epcoritamab	2	36	0	0.00	8.4	68.5	3	0	55.6	NA
	Thieblemont	2022		epcoritamab	1/2	157	9	5.73	10.7	64	3	38.9	63.1	exp
	Hsu	2024		glofitamab	RW	31	3	9.68	15.9	58	5	NR	56.0	NA
	Song	2024		glofitamab	1	27	0	0.00	15	57.5	2	20	66.7	exp
	Atesoglu	2023		glofitamab	RW	43	5	11.63	5.7	54	4	0	37.0	NA
	Dickinson	2022		glofitamab	2	154	8	5.19	12.6	66	3	33	52.0	NA
	Budde	2024	A2	mosunetuzumab	1/2	129	8	6.20	40.8	64	3	11.6	36.4	esc+exp
	Budde	2024	B	mosunetuzumab	2	98	3	3.06	23.9	68	2	35.7	59.2	plus pola
	Olszewski	2023		mosunetuzumab	2	40	1	2.50	32	65	0	0	87.5	plus CHOP
	Bartlett	2023		mosunetuzumab	2	88	3	3.41	10.1	66.5	3	29.5	42.0	NA
MCL	Philipps	2024		glofitamab	1/2	60	9	15.00	19.6	72	2	3.3	85.0	NA
NHL	Goebeler	2016		blinatumomab	1	34	1	2.94	NR	62	3	NR	NR	exp
	Hutchings	2021		glofitamab	1	171	2	1.17	13.5	64	3	1.8	53.8	esc+exp
	Bannerji	2022		odronextamab	1	145	7	4.83	4.2	67	3	29	51.0	esc+exp

(*Continued on next page*)

Table 1. Continued

Entity	First author	Year	Cohort	Product	Setting	Cohort size	Non-relapse deaths	NRM point estimate, %	Follow- up, months	Age, y, median	Prior treatment lines, median	Prior CAR-Ts, %	ORR, %	Treatment mode
	D'Souza	2022		ABBV-383 (etentamig)	1	51	1	1.96	8.2	68	4	0	59.0	exp
	Bahlis	2023		elranatamab	1	55	5	9.09	12	64	5	9.1	63.6	exp
	Lesokhin	2023		elranatamab	2	123	14	11.38	14.7	68	5	0	61.0	NA
	Bumma	2024		linvoseltamab	1/2	117	6	5.13	1[a]	70	5	NR	70.9	exp
MM	Chari	2022	A	talquetamab	1	30	0	0.00	11.7	62	6	NR	70.0	exp
	Chari	2022	B	talquetamab	1	44	3	6.82	4.2	64	5	NR	64.0	exp
	Lebreton	2024		teclistamab	RW	15	1	6.67	5.4	68	4	NR	NR	NA
	Dima	2024		teclistamab	RW	106	3	2.83	3.8	66.5	6	40	66.0	NA
	Moreau	2022		teclistamab	1/2	165	19	11.52	14.1	64	5	0	63.0	exp

1, phase 1 trial; 1/2, phase 1/2 trial; 2, phase 2 trial; IL, indolent lymphoma; LBCL, large B-cell lymphoma and other aggressive lymphomas; MCL, mantle cell lymphoma; MM, multiple myeloma; NA, not applicable; NHL, B-cell non-Hodgkin lymphoma (when subcohorting in other mentioned entities was not possible based on the reported data); NR, not reported; NRM, non-relapse mortality; ORR, overall response rate; RW, real-world analysis. In the column "treatment mode," additional information on the treatment is given: cons, bispecific antibody (BsAb) therapy as a consolidation concept after another treatment regimen; exp, dose expansion cohort of early clinical trials; exp + esc, mixed cohort of dose escalation and dose expansion (further subcohorting and inclusion of only dose expansion cohort not possible based on reported data); plus CHOP, combination therapy of BsAb with CHOP chemotherapy; plus pola, combination therapy of BsAb with polatuzumab-vedotin.

[a]Follow-up was restricted to 1 month, given that NRM cases were only reported during this period.

different products did not significantly impact the NRM estimates; there was neither a significant difference between NHL products (odronextamab 8.6%, glofitamab 5.4%, blinatumomab 4.0%, mosunetuzumab 3.5%, epcoritamab 2.1%, p = 0.33; Figures 3A and 3B) nor between MM products (elranatamab 10.7%, teclistamab 6.5%, linvoseltamab 5.1%, talquetamab 4.0%, ABBV-383 [etentamig] 2.0%, p = 0.19; Figures 3C and 3D). In NHL, the pre-specified subgroup analyses revealed significantly higher NRM point estimates in the RW studies compared to the cohorts treated within clinical trials (Figure S3A), but no significant impact of age, prior CAR-T exposure, number of previous therapy lines, overall response rate, or the duration of follow-up on the NRM point estimates (panel A of Figures S4–S8). In MM, significantly higher NRM point estimates were observed in cohorts with overall response rates above the median (9.9% vs. 4.0%, p = 0.005; Figure S7B) and follow-up time above the median (10.2% vs. 4.2%, p = 0.003; Figure S8B), but no significant impact of the treatment setting (CT vs. RW), age, or prior CAR-T exposure (panel B of Figures S3–S5). Between the different target antigens, no difference in NRM point estimates was observed either in NHL (CD19 vs. CD20) or in MM (BCMA vs. GPRC5D) (Figure S9). In the two NHL studies included in our analysis where BsAb were combined with other therapeutic regimens, NRM was not significantly higher with combination therapy (Figure S10).[24,40] Additionally, there was no significant difference in NRM point estimates between products that are already approved and those not yet approved for the investigated entity (Figure S11).

Meta-regression analysis reveals a similar NRM for BsAb and CAR-T therapy when accounting for key study-level confounders

While both BsAb and CAR T-cell therapies hold significant promise, the lack of direct comparative studies poses a challenge to optimizing clinical decision-making and understanding their relative safety profiles. To address this gap, we integrated our previously published systematic review on NRM after CAR T-cell therapy.[18] To align the inclusion criteria of both studies, for this meta-regression we excluded BsAb that were not yet approved by the US Food and Drug Administration (FDA) or the European Medicines Agency for the investigated disease entity at the time of submitting this study. On univariate analysis, the NRM point estimate was numerically lower with BsAb therapy (4.9% vs. 6.8%, p = 0.09; Figure 4A). Subgroup analysis indicated that this difference was primarily driven by NHL studies (4.3% vs. 6.5%, p = 0.05; Figure 4B), whereas NRM estimates were elevated for both treatment modalities in MM (BsAb: 7.2%, CAR-T: 8.0%, p = 0.73; Figure 4C). Next, we performed a meta-regression analysis incorporating 68 study cohorts and 8,592 patients adjusted for disease type, prior treatment lines, treatment setting, and follow-up duration, excluding 3 studies that did not differentiate between lymphoma subtypes. When accounting for these key study-level risk factors, the treatment modality had no statistically significant effect on NRM estimates (p > 0.9; Figure 4D). Notably, extended follow-up emerged as the only study-level covariate showing a trend toward influencing NRM (p = 0.06).

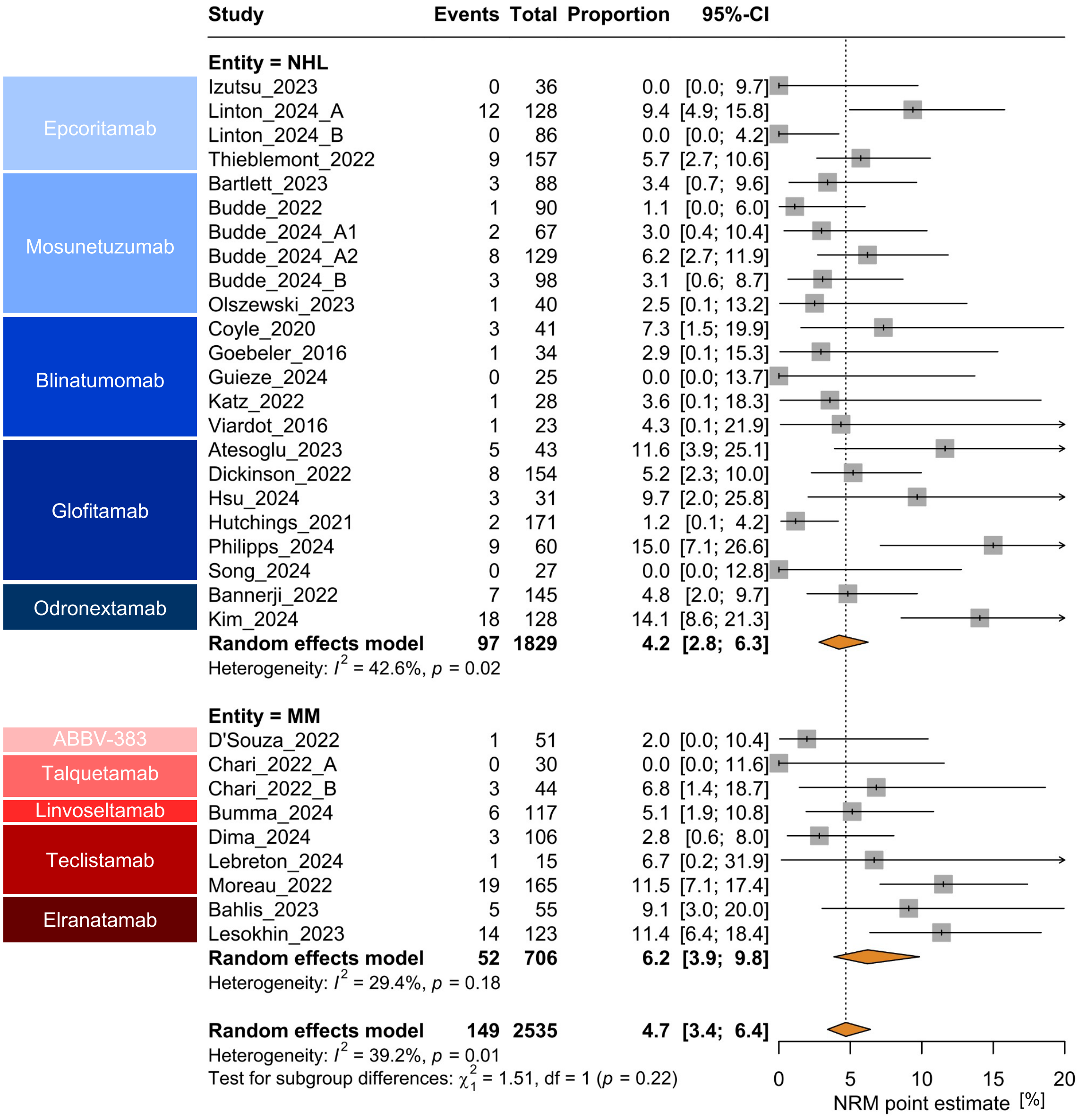

Figure 2. Forest plot of NRM point estimates across all study cohorts and stratified by entity

Forest plot displaying NRM point estimates and 95% confidence intervals, calculated using a random effects model. Studies are arranged by disease type, with B-cell non-Hodgkin lymphoma (NHL, blue) presented at the top and multiple myeloma (MM, red) below. Different shades of blue and red represent therapeutic products within each disease category. The disease-specific and the overall NRM point estimates are emphasized in bold black. Measures of heterogeneity, including I^2 values, are provided (with I^2 values between 25% and 50% indicating low-to-moderate heterogeneity).

When excluding COVID-19 fatalities, we noted more pronounced differences between both treatment modalities (3.6% vs. 6.1%, p = 0.003; Figure S12). For example, significantly lower NRM estimates were seen for BsAb vs. CAR-T therapy in NHL (3.1% vs. 5.8%, p = 0.005), which also extended to numerically decreased NRM in MM (5.0% vs. 7.2%, p = 0.16). In addition, a trend toward lower NRM was observed with BsAb compared to CAR-T therapy in the meta-regression analysis, excluding COVID-19 deaths (adjusted p = 0.10; Table S2).

Infections are the main driver of NRM following BsAb therapy

To elucidate NRM etiology, we extracted the available data for all 149 reported non-relapse deaths among our total BsAb study cohort of 2,535 patients. For 138/149 cases (92.6%), the underlying cause of death was indicated, which we classified into one of 7 groups as outlined in the materials and methods (Figure 5A). If the cause of death did not match any of these groups, it was classified under "other" (8/149 cases; Table S3). In 11/149 cases (7.4%), the specific cause of death was reported as "unknown."

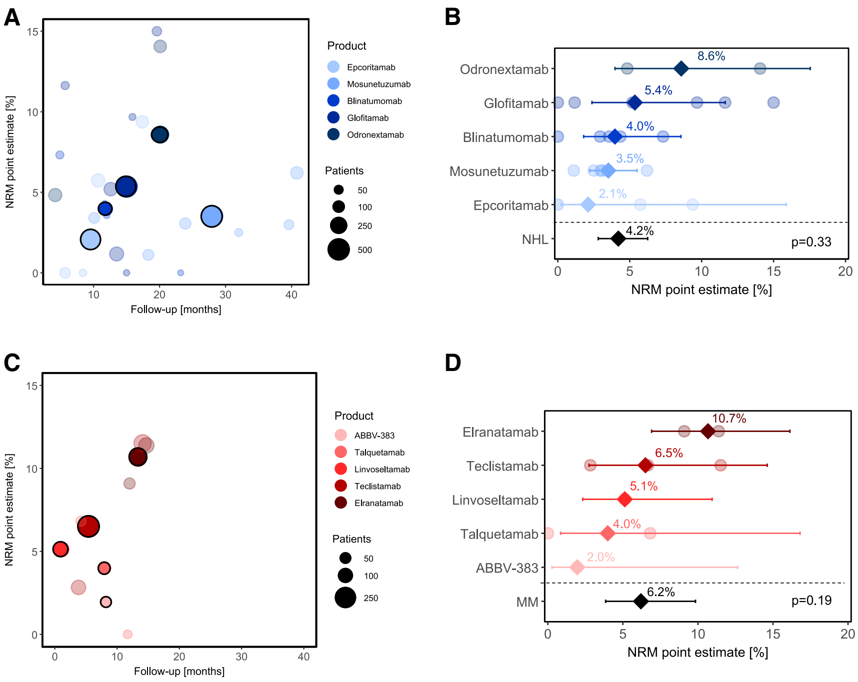

Figure 3. Disease-specific NRM point estimates for different BsAb products

(A and C) Bubble plots showing NRM point estimates relative to follow-up duration for NHL (blue) and MM (red). Different shades of blue and red represent therapeutic products within each disease category. Each bubble represents a cohort, with bubble size proportional to the cohort's total patient count. Aggregated NRM point estimates for all cohorts within one entity are encircled in black. (B and D) Comparison of aggregated NRM point estimates and 95% CIs across the different BsAb products for NHL (B) and MM (D). The *p* values for the comparisons of NRM point estimates by disease entity and BsAb product were calculated using the test for subgroup differences (random effects model).

Interestingly, more than 70% of all reported non-relapse deaths were attributed to infections (107/149, 71.8%). The cause of infection-related death was not further specified in 30 cases. Among the 77 non-relapse deaths where the pathogen was reported, the majority were attributed to COVID-19 infection (51/77, 66.2%). Of note, most of the included studies accrued during the COVID-19 pandemic (Table S4). In a pre-specified sensitivity analysis excluding COVID-19-related deaths, we noted an overall NRM point estimate of 3.5% (95% CI 2.6%–4.6%) for our study cohort (Table S5). Still, a significant proportion of NRM cases in patients receiving BsAb were due to bacterial (13/77, 16.9%), non-COVID viral (9/77, 11.7%), or fungal infections (4/77, 5.2%). A detailed breakdown of pathogens resulting in death due to infection is provided in Table S6, including some rarer opportunistic infections such as five cases of progressive multifocal leukoencephalopathy (PML), four cases of adenoviral infections, and three cases of *Pneumocystis jirovecii* pneumonia (PJP). We were not able to resolve whether these specific patients had previous CAR-T exposure.

Cardiovascular or respiratory (CVR) events were the second most common cause of NRM, resulting in 10/149 (6.7%) non-relapse deaths. Four non-relapse deaths were attributed to either a secondary malignancy unrelated to the underlying lymphoma/myeloma or organ failure, each accounting for 2.7% of cases. Three additional deaths (2.0%) resulted from hemorrhages. The prototypical immunotoxicities ICANS and CRS each led to only 1 of the 149 non-relapse deaths (0.7% each).

When comparing NHL and MM, we observed no significant difference in the distribution of causes of death (Figure 5B) or pathogens responsible for the infection-related deaths (Figure S13). However, we noted a significantly different cause-of-death pattern with BsAb compared to CAR-T treatment, with a relative skewing toward infection-related deaths in the BsAb group (Figure 5C). A closer look at the cause attribution for infection-related deaths revealed that there were fewer cases classified as not otherwise specified (NOS) in the BsAb cohort (Figure 5D), indicating improved infection reporting relative to CAR-T therapy.

DISCUSSION

In this systematic review and meta-analysis, we outline the comparative incidence and causes of NRM with bispecific antibodies across a spectrum of hematologic malignancies. Our findings highlight a consistent NRM point estimate of 4.7% across studies, with no significant differences observed across disease entities or BsAb products. While NRM point estimates were numerically lower with BsAb vs. CAR-T treatment, this observation did not extend to the meta-regression analysis accounting for key study-level risk factors, including follow-up. Infections were by far the main cause of NRM, responsible for over 70% of non-relapse deaths.

The introduction of BsAb into clinical practice has broadened the therapeutic landscape for patients with relapsed or refractory B-cell malignancies. Their off-the-shelf availability and broad applicability make them an attractive option, particularly in scenarios where CAR T-cell therapy or HCT may not be feasible due to logistical, medical, or socioeconomic factors.[3,8] As these therapies gain traction, NRM as a measure of treatment-associated safety becomes a critical component in informing patient-centered decision-making.

Relative to CAR-T therapy, BsAb carry a generally favorable safety profile with mainly low-grade CRS and anecdotal ICANS.[12,50] However, our analysis found that this did not translate into a significantly reduced NRM when controlling for key study-level risk factors, highlighting the critical importance of infections in heavily pre-treated patients. However, it should be noted that many of the BsAb studies

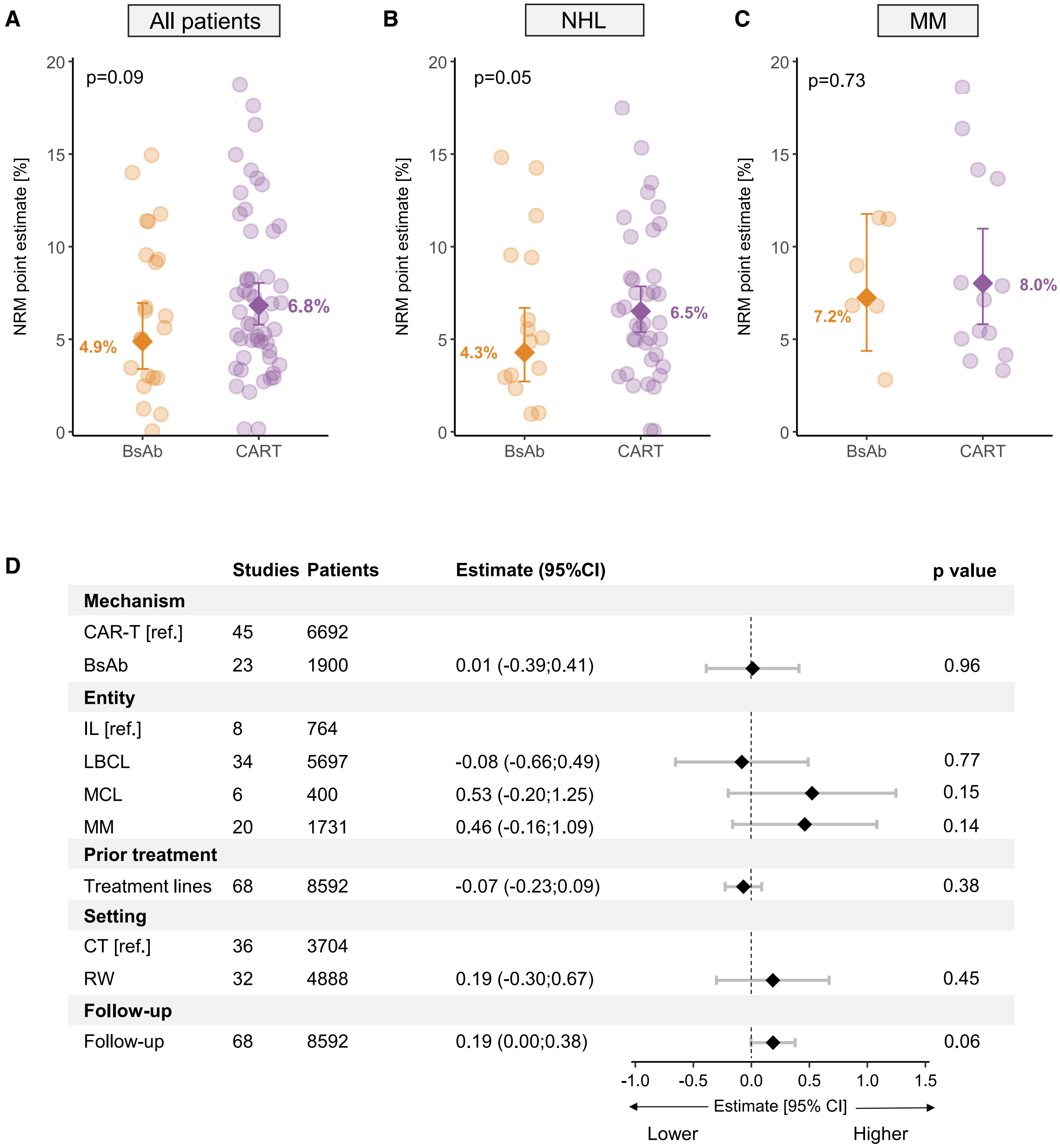

Figure 4. Comparative analysis of NRM point estimates between BsAb and CAR T-cell therapy

(A–C) Aggregated NRM point estimates and 95% CIs are compared between BsAb- and CAR-T-treated patients across all disease entities (A) and specifically for NHL (B) and MM (C). (D) Multivariable meta-regression analysis using random effects models for study characteristics. The integrated forest plot displays model estimates, with 95% CI for each study variable, such as mechanism (CAR-T vs. BsAb), disease entity, treatment setting, median number of treatment lines, follow-up time (years). Reference levels for the calculation of model estimates and respective *p* values are provided for each study feature.

accrued during the COVID-19 pandemic and that follow-up on many of these studies remains short. Indeed, the NRM differences between CAR-T and BsAb therapy were more pronounced upon exclusion of COVID-19 fatalities in our study (Figure S12). Generally, the observed NRM estimates with BsAb therapy were lower or at least comparable to autologous[51–55] and lower compared to allogeneic HCT,[56–59] with implications for treatment sequencing.[60,61]

The comparatively low NRM and favorable early immunotoxicity profile suggests that BsAb therapy not only offers at least comparable safety to other established treatments but also holds promise for integration into combination regimens, potentially enhancing therapeutic outcomes without significantly increasing NRM. In line with this, the two NHL studies where BsAb were utilized within combination regimens did not yield significantly higher NRM (Figure S10).[24,40]

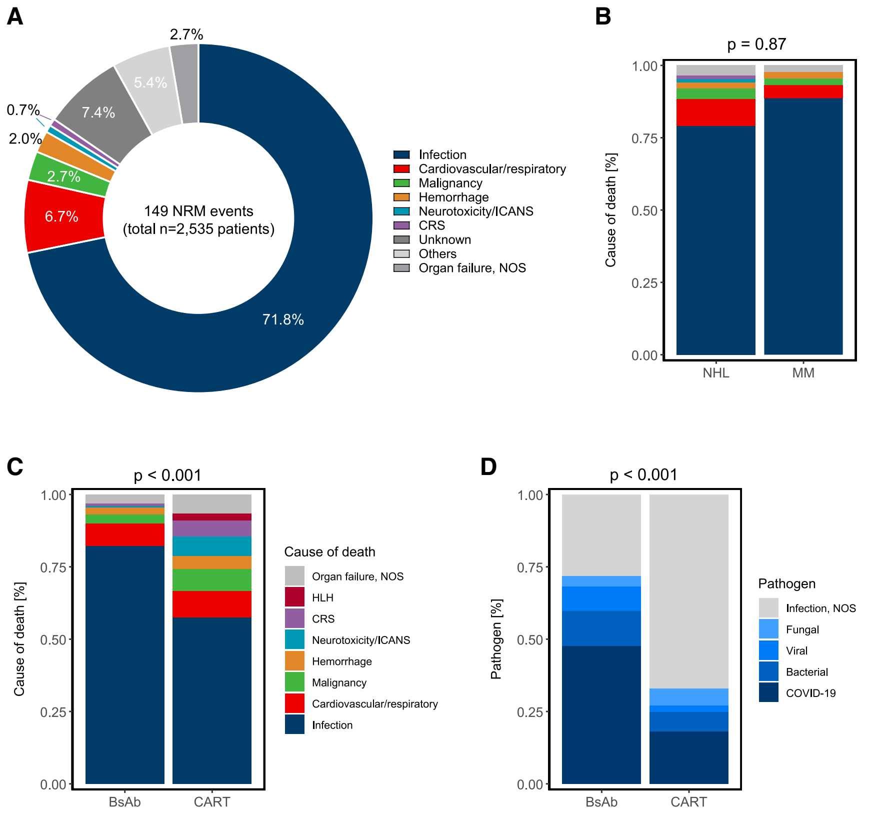

Figure 5. Distribution of causes of NRM

(A) Donut plot illustrating the distribution of causes of death in the entire BsAb study cohort. Specific causes of death are represented in distinct colors, while undefined and unclassifiable causes are shown in shades of gray. (B) Comparison of NRM causes between NHL and MM patients. (C and D) Comparison of NRM causes (C) and the distribution of pathogens responsible for fatal infections (D) between BsAb- and CAR-T cell-treated patients. Statistical significance was evaluated using the χ^2 distribution test.

One study evaluated BsAb in combination with polatuzumab, a well-tolerated antibody-drug conjugate,[24] while the other focused on BsAb combined with the combination of cyclophosphamide, doxorubicin hydrochloride (hydroxydaunorubicin), vincristine sulfate (Oncovin), and prednisone (CHOP) as a first-line treatment.[40] However, the randomized STARGLO trial (published following completion of our study) suggested that adding a BsAb to existing chemotherapies in the relapsed or refractory setting, while efficacious, may also increase the risk of fatal infections.[62] Among the 15 deaths during treatment (representing 8.3% of the experimental arm), 10 were related to infection (7 due to COVID-19), illustrating the cumulative infection risk in these patients.

Another advantage of bispecific antibody therapy, in contrast to CAR-T and HCT, is the ability to discontinue the treatment if necessary or reduce the dose or frequency of administration, providing an additional layer of flexibility in managing treatment-related toxicities.[63] However, the cumulative exposure to B cell-depleting therapy over long periods of time may potentiate infection risk[64] and facilitate chronic T-cell exhaustion.[17]

In this meta-analysis, infections accounted for 71.8% of all non-relapse deaths with BsAb therapy. Notably, COVID-19 was a significant contributor, accounting for approximately two-thirds of infection-related fatalities with an identifiable pathogen. Much of the data analyzed was collected during the COVID-19 pandemic (2020–2023), which may have in part influenced the observed NRM rates. Indeed, the prespecified sensitivity analysis excluding COVID-19-related deaths yielded a lower NRM estimate of 3.5% (Table S5). These findings underscore the importance of preventing infections, particularly through comprehensive vaccination against SARS-CoV-2 and other preventable pathogens like *pneumococcus*. Additionally, rare opportunistic infections such as PML, PJP, and adenoviral infections contributed to fatalities, highlighting the heightened vulnerability of these patients. Due to the nature of our study, it was impossible to ascertain whether these specific patients had also received prior CAR T-cell therapy, which can confer long-lasting T-cell deficits.[65–67] Examining lymphocyte subpopulations before BsAb treatment may be prescient and could guide the use and duration of antimicrobial prophylaxis, especially for *Pneumocystis*.[14,50]

Various factors heighten the susceptibility of patients receiving bispecific antibodies to infectious complications, including Tcell exhaustion, prior chemotherapy, and the underlying malignancy.[11,13,17] Furthermore, neutropenia and on-target/off-tumor effects, such as B-cell aplasia and hypogammaglobulinemia, further elevate the risk of infections.[15,16] Addressing these vulnerabilities is critical, and supportive strategies, such as granulocyte-colony-stimulating factor and immunoglobulin (Ig) substitution, may help mitigate these risks.[14,15,50,68] The importance of prophylactic measures is underscored by findings from the RedirecTT-1 trial of teclistamab/talquetamab, which reported infection-related deaths in 11 of 94 patients, yet reveals inconsistent implementation of prophylaxis, with approximately 18% of patients not receiving antiviral prophylaxis, around 50% lacking protection against PJP, and 43% of those with non-IgG myeloma never receiving IgG replacement therapy.[69] In particular, Ig substitution should be strongly considered as a key strategy to lower NRM.[15] In multiple myeloma, for example, prophylactic intravenous Ig (IVIG) is recommended for patients experiencing recurrent bacterial infections or with IgG levels below 400 mg/dL.[64,70] Consensus recommendations of an expert panel from the Academic Consortium to Overcome Multiple Myeloma through

Innovative Trials advocate for IVIG as primary prophylaxis, independent of IgG levels, starting in the second month of treatment and continuing until either therapy completion or IgG levels exceed 400 mg/dL, whichever occurs later.[16] However, the available data from the included studies lack the necessary detail to determine the impact of consistent IVIG use on patient outcomes (Table S4). This highlights the necessity of standardized documentation and adherence to prophylactic strategies to better understand their role in reducing NRM and improving patient outcomes.

Our analysis revealed key limitations in the current body of evidence, particularly the heterogeneous reporting of NRM endpoints. Cumulative NRM rates, critical for understanding the trajectory of mortality risk over time in relation to other competing events like relapse or progression,[19,71–73] were not at all reported. Moreover, categorization inconsistencies across studies may have introduced variability. Standardized definitions and reporting guidelines for infections are essential to improve data reliability and comparability, as recently implemented for immune effector cell-associated hemophagocytic lymphohistiocytosis-like syndrome (IEC-HS), immune effector cell-associated enterocolitis, and immune effector cell–associated hematotoxicity (ICAHT).[74–78] Another challenge is the limited availability of robust real-world data. For example, the existing and included RW data were confined to glofitamab for NHL and teclistamab for MM. Clinical trial cohorts often exclude patients with poor performance status or significant comorbidities, limiting the generalizability of findings. Additionally, our meta-analysis could account only for cohort-level differences and not individual patient factors, such as tumor burden, systemic inflammation, number of BsAb cycles, or prior therapies, which likely influence NRM risk.

Methodological limitations relate to the publication bias detected in the Egger's test and the discrepancy in the inclusion periods for BsAb and CAR-T studies, potentially affecting comparisons. While a subgroup analysis comparing BsAb records published after March 31, 2024 with those published before found no significant differences regarding NRM point estimates (Figure S14), the different search window may still have impacted our results. Of note, we did not detect significant effects from factors such as the inclusion of unapproved BsAb or their use as part of combination therapies. These studies were retained to provide a more comprehensive and real-world perspective on treatment outcomes, although certain biases and limitations due to their inclusion cannot be entirely eliminated. Despite these limitations, we believe that the overall analysis remains robust, and the findings reflect a clinically relevant, broad view of BsAb therapy. To test the robustness of our main study findings, we performed not only several subgroup analyses (Figures S3–S11), but also several sensitivity analyses (Table S6), showing stable results. For example, NRM estimates were comparable when applying fixed-effect vs. random-effect models.

As BsAb are increasingly introduced earlier in treatment lines, NRM becomes a critical factor in guiding their sequencing and evaluating their safety profile relative to other treatment options. This is particularly important for patients ineligible for more intensive therapies, where minimizing treatment-related mortality is paramount. Future studies should prioritize robust and detailed reporting of NRM, particularly with respect to infection-related deaths, to better inform clinical decision-making. Such reports should encompass the infection type (viral, bacterial, fungal), confirmation through microbiological testing, identified pathogens, the timing of infection, infection source and severity, and whether the infection occurred early vs. late in the treatment process. Collecting this information can help further refine existing evidence-based guidelines focused on infection prevention and management for patients receiving BsAb therapy.[14,50,64,70] Efforts should focus on identifying subgroups at heightened risk of NRM and tailoring supportive care interventions to their needs. For example, differentiating between time-limited therapies and those administered until severe toxicity is observed may further refine the balance between efficacy and safety.

In conclusion, our analysis highlights the importance of improving BsAb safety reporting and infection management. With infections being the leading cause of NRM across all disease types and products, further developing evidence-based guidelines for infection prevention, including vaccination, Ig substitution, and antimicrobial prophylaxis, is crucial. Enhanced reporting and long-term follow-up will enable a more nuanced understanding of NRM and its drivers, ultimately supporting safer use of BsAb in hematological malignancies.

MATERIALS AND METHODS

Study design and literature search

The methods used in this study were aligned with a previous meta-analysis conducted by our group.[18] We screened all studies for the FDA-approved bispecific antibodies in MM (teclistamab, talquetamab, elranatamab) and NHL (glofitamab, epcoritamab, mosunetuzumab) and unapproved bispecific antibodies for these malignancies whose phase 1 and/or 2 results have been fully published (odronextamab, blinatumomab, ABBV-383 [etentamig], linvoseltamab). A systematic search was performed in the PubMed and Embase databases for articles published up to October 10, 2024, using combined key words for each of the bispecific antibodies along with "lymphoma" or "myeloma" (see Data S1). Case studies, reviews, conference abstracts, and meta-analyses were excluded. After screening titles and abstracts, eligible publications were assessed by two independent investigators (T.T. and M.A.) based on the following set of inclusion criteria that had to be fully satisfied.

(1) Adult cancer patients with either IL, LBCL, MM, or MCL
(2) Use of bispecific antibody products approved by the FDA, or a bispecific antibody with phase 1 and/or 2 results published as a full manuscript
(3) Cohort of at least 15 patients treated with the same dose of the bispecific antibody
(4) Reporting of the absolute number of non-relapse deaths in the treated cohort
(5) Full-text available in English

All included articles were examined for potential duplicate reporting. In cases where two studies covered the same patient population, the study with longer follow-up was selected.

For the comparison with NRM in CAR-T cell-treated patients, we incorporated records from our previous meta-analysis on CAR-T-treated myeloma and lymphoma patients, with screening and extraction methods detailed there.[18] The inclusion criteria were similar, with publications included until March 31, 2024.

This study followed the PRISMA(-P) guidelines (see Data S1) and was prospectively registered to the PROSPERO database on December 27, 2023 (study no. CRD42023494258).[79] The PRISMA checklist can be provided by the authors upon reasonable request. Institutional review board approval was not sought as this study did not represent human participant research.

Data extraction

Data collection was performed by two independent investigators (T.T. and M.A.) and included date of publication, number of patients, disease entities, utilized bispecific antibody, time frame of patient inclusion, median follow-up time, median age of the cohort, treatment history (median number of previous lines, proportion of patients with prior HCT or CAR-T therapy), treatment setting (RW study vs. CT, including trial phase), and overall response rate (ORR). Furthermore, we determined whether BsAb were applied as monotherapy, combination therapy, or consolidation therapy. The primary outcome was the number and causes of death. If a patient's remission status at the time of death was not clearly specified, then the death was considered an NRM case. If multiple dosing schemes were used or multiple entities were treated within a single study, then the reported data were assigned to separate cohorts and analyzed individually, provided that the quality of reporting allowed for such separation. Dose escalation cohorts were excluded from analysis where separation was possible.

Quality assessment

The Joanna Briggs Institute appraisal tool was applied to assess study bias of included articles (Table S1). Visual inspection of funnel plot asymmetry and Egger regression tests were used to assess reporting bias (Figure S2).[80,81]

Statistical analysis

Data analysis was conducted in R (version 4.4.1) using the metafor (version 4.6-0) and meta (version 8.0-1) packages. NRM point estimates and estimates for NRM per patient-year were derived by performing random-effects meta-analyses of single proportions through a generalized linear mixed model.[82] Patient-years were calculated using the number of included patients and the median follow-up time in years of each respective cohort. The Clopper-Pearson interval provided 95% CIs for proportions.[83] Forest plots were used to visually represent NRM outcome data. For each meta-analysis, heterogeneity of pooled effect sizes was assessed with the Q statistic and quantified using I^2, where 25%, 50%, and 75% reflect low, moderate, and high between-study heterogeneity, respectively.[84] Continuous variables were reported as medians and interquartile ranges. The chi-squared (χ^2) test was used to examine the distribution of NRM-related deaths across sub-cohorts.

Meta-analyses

Separate meta-analyses were conducted for predefined subgroups to compare NRM point estimates based on disease entity, BsAb product, treatment setting, age, the number of prior therapy lines, prior CAR-T exposure, overall response rate, and follow-up. Subgroup comparisons were evaluated using the test for subgroup differences within a random effects model, and corresponding p values were calculated.

Meta-regression analysis

A meta-regression analysis was performed to examine the association between NRM point estimates and the following variables: treatment modality (BsAb vs. CAR-T), disease entity, treatment setting (e.g., CT vs. RW), treatment line, and follow-up time. The meta-regression model was calculated based on random effects models using the maximum likelihood estimator.[85] Individual model coefficients and respective CIs were tested using the Knapp-Hartung method.[86] The stability of model estimates was validated by performing permutation testing.[87]

Sensitivity analysis

To validate the robustness of the main findings, meta-analyses were repeated after (1) testing fixed effects models, (2) excluding COVID-related deaths, and (3) excluding phase 1 studies where it was not feasible to extract a subcohort of patients treated with a single-dose scheme.[88]

Cause of death analysis

To test for cause of death distributions between subgroups, causes of death were classified into one of the following groups: infection, secondary malignancy, CRS, ICANS, cardiovascular or respiratory, hemorrhage, organ failure NOS, other, or unknown. Infections were further classified into COVID-19, bacterial, viral, fungal, or infection NOS. Donut plots to visualize the cause of death distribution were generated using GraphPad Prism (version 10.4.0).

DATA AVAILABILITY

All data needed to evaluate the conclusions in the paper are present in the manuscript and/or the supplemental information. Data from primary studies are publicly available within the databases listed in the supplemental information. All codes were adapted using R software, version 4.4.1 (meta package 8.0–1, metafor package 4.6–0). If there are further questions, the corresponding author should be contacted.

ACKNOWLEDGMENTS

First and foremost, we acknowledge the numerous patients whose data contributed to this study, along with the dedicated research professionals driving progress in this rapidly advancing field. T.T. received a fellowship from the School of Oncology of the German Cancer Consortium (DKTK). D.M.C.d.S. received the Walter Benjamin Fellowship from the Deutsche Forschungsgemeinschaft (DFG, German Research Foundation, grant no. 525171148). E.R.S.C. receives research funding from Arnold Ventures. D.K.H. is supported by the National Cancer Institute (grant no. R01CA281756-01A1) and the

Pentecost Family Myeloma Research Center. K.R. acknowledges funding from the Else Kröner Forschungskolleg (EKFK) within the Munich Clinician Scientist Program (MCSP), the Bruno and Helene Jöster Foundation, and the "CAR-T Control" translational group within the Bavarian Center for Cancer Research (grant no. BZKF-#TLG-22). R.S., K.R., and M.-A.P. were supported by a Memorial Sloan Kettering Cancer Center Core grant (P30 CA008748) from the National Institutes of Health/National Cancer Institute. R.S. was supported by an NIH-NCI K-award (grant no. K08CA282987). The content is solely the responsibility of the authors and does not necessarily represent the official views of the National Institutes of Health. The graphical abstract was created in BioRender (https://BioRender.com/dw8w1oc).

AUTHOR CONTRIBUTIONS

Conceptualization: T.T. and K.R. Investigation: T.T., M.A., R.S., G.I., E.R.S.C., D.K.H., S.Z.U., G.S., M.-A.P., D.M.C.d.S., and K.R. Formal analysis and visualization: T.T. and D.M.C.d.S. Methodology: T.T., D.M.C.d.S., and K.R. Writing – original draft: T.T., D.M.C.d.S., and K.R. Writing – review & editing: T.T., M.A., R.S., G.I., E.R.S.C., D.K.H., S.Z.U., G.S., M.-A.P., D.M.C.d.S., and K.R. All authors read and approved the final manuscript.

DECLARATION OF INTERESTS

R.S. reports speaker honorarium from Incyte. G.I. reports honoraria and/or travel support from Abbvie, AstraZeneca, Autolus, Bristol Myers Squibb, Kite/Gilead, Miltenyi, and Novartis. D.K.H. reports research funding from Bristol Myers Squibb, Karyopharm, Kite Pharma, and Adaptive Biotech; her consulting or advisory roles include Bristol Myers Squibb, Janssen, Legend Biotech, Pfizer, Kite Pharma, AstraZeneca, and Karyopharm. S.Z.U. reports serving on an advisory committee for and receiving research funding from Janssen, GlaxoSmithKline, Gilead Sciences, Seattle Genetics, Sanofi, SkylineDX, Takeda, Celgene, Bristol Myers Squibb, Amgen, and AbbVie; serving on an advisory committee for EdoPharma, Genetech, K36 Therapeutics, Moderna, Oncopeptides, Novartis, SecuraBio, and TeneoBio; and receiving research funding from Merch, Array Biopharma, and Pharmacyclics. G.S. reports consultancy with AbbVie, BeiGene, Bristol Myers Squibb, Debiopharm, Epizyme, Genentech/Roche, Genmab, Innate Pharma, Incyte, Ipsen, Janssen, Kite/Gilead, Merck, Modex, Molecular Partners, Nurix, Orna Therapeutics, Pfizer, and Treeline; he has received research funding from AbbVie, Genentech, Genmab, Janssen, Ipsen, and Nurix. M.-A.P reports honoraria from Adicet, Allogene, Caribou Biosciences, Celgene, Bristol Myers Squibb, Equilium, Exevir, ImmPACT Bio, Incyte, Karyopharm, Kite/Gilead, Merck, Miltenyi Biotec, MorphoSys, Nektar Therapeutics, Novartis, Omeros, OrcaBio, Sanofi, Syncopation, Takeda, VectivBio AG, and Vor Biopharma; he serves on data safety monitoring boards for Cidara Therapeutics and Sellas Life Sciences and on the scientific advisory board of NexImmune; he has ownership interests in NexImmune, Omeros, and OrcaBio; and he has received institutional research support for clinical trials from Allogene, Incyte, Kite/Gilead, Miltenyi Biotec, Nektar Therapeutics, and Novartis. K.R. reports research funding, consultancy, honoraria, and travel support from Kite/Gilead; honoraria from Novartis; consultancy and honoraria from Bristol Myers Squibb/Celgene; and travel support from Pierre-Fabre.

SUPPLEMENTAL INFORMATION

Supplemental information can be found online at https://doi.org/10.1016/j.ymthe.2025.03.048.

REFERENCES

1. Klein, C., Brinkmann, U., Reichert, J.M., and Kontermann, R.E. (2024). The present and future of bispecific antibodies for cancer therapy. Nat. Rev. Drug Discov. *23*, 301–319. https://doi.org/10.1038/s41573-024-00896-6.

2. Wang, Z., Yin, C., Lum, L.G., Simons, A., and Weiner, G.J. (2021). Bispecific antibody-activated T cells enhance NK cell-mediated antibody-dependent cellular cytotoxicity. J. Hematol. Oncol. *14*, 204. https://doi.org/10.1186/s13045-021-01216-w.

3. Devasia, A.J., Chari, A., and Lancman, G. (2024). Bispecific antibodies in the treatment of multiple myeloma. Blood Cancer J. *14*, 158. https://doi.org/10.1038/s41408-024-01139-y.

4. Falchi, L., Vardhana, S.A., and Salles, G.A. (2023). Bispecific antibodies for the treatment of B-cell lymphoma: promises, unknowns, and opportunities. Blood *141*, 467–480. https://doi.org/10.1182/blood.2021011994.

5. Cordas dos Santos, D.M., Toenges, R., Bertamini, L., Alberge, J.-B., and Ghobrial, I.M. (2024). New horizons in our understanding of precursor multiple myeloma and early interception. Nat. Rev. Cancer *24*, 867–886. https://doi.org/10.1038/s41568-024-00755-x.

6. Subklewe, M., Magno, G., Gebhardt, C., Bücklein, V., Szelinski, F., Arévalo, H.J.R., Hänel, G., Dörner, T., Zugmaier, G., von Bergwelt-Baildon, M., et al. (2024). Application of blinatumomab, a bispecific anti-CD3/CD19 T-cell engager, in treating severe systemic sclerosis: A case study. Eur. J. Cancer *204*, 114071. https://doi.org/10.1016/j.ejca.2024.114071.

7. Subklewe, M. (2021). BiTEs better than CAR T cells. Blood Adv. *5*, 607–612. https://doi.org/10.1182/bloodadvances.2020001792.

8. Trabolsi, A., Arumov, A., and Schatz, J.H. (2024). Bispecific antibodies and CAR-T cells: dueling immunotherapies for large B-cell lymphomas. Blood Cancer J. *14*, 27. https://doi.org/10.1038/s41408-024-00997-w.

9. Budde, L.E., Assouline, S., Sehn, L.H., Schuster, S.J., Yoon, S.S., Yoon, D.H., Matasar, M.J., Bosch, F., Kim, W.S., Nastoupil, L.J., et al. (2024). Durable Responses With Mosunetuzumab in Relapsed/Refractory Indolent and Aggressive B-Cell Non-Hodgkin Lymphomas: Extended Follow-Up of a Phase I/II Study. J. Clin. Oncol. *42*, 2250–2256. https://doi.org/10.1200/JCO.23.02329.

10. Sharma, S., Wang, D., Lon, H.-K., Soltantabar, P., Viqueira, A., Czibere, A., Hickman, A., White, J.L., and Elmeliegy, M. (2022). A Systematic Meta-Analysis of Cytokine Release Syndrome Incidence in B-Cell Maturation Antigen-Targeting Chimeric Antigen Receptor T-Cell Therapy and Bispecific Antibodies for Patients with Relapsed and/or Refractory Multiple Myeloma. Blood *140*, 10036–10038. https://doi.org/10.1182/blood-2022-169836.

11. Markouli, M., Ullah, F., Unlu, S., Omar, N., Lopetegui-Lia, N., Duco, M., Anwer, F., Raza, S., and Dima, D. (2023). Toxicity Profile of Chimeric Antigen Receptor T-Cell and Bispecific Antibody Therapies in Multiple Myeloma: Pathogenesis, Prevention and Management. Curr. Oncol. *30*, 6330–6352. https://doi.org/10.3390/curroncol30070467.

12. Kim, J., Cho, J., Lee, M.H., Yoon, S.E., Kim, W.S., and Kim, S.J. (2024). CAR T cells vs bispecific antibody as third- or later-line large B-cell lymphoma therapy: a meta-analysis. Blood *144*, 629–638. https://doi.org/10.1182/blood.2023023419.

13. Reynolds, G.K., Maclean, M., Cliff, E.R.S., Teh, B.W., Thursky, K.A., Slavin, M.A., Anderson, M.A., and Hawkes, E.A. (2024). Infections in patients with lymphoma treated with bispecific antibodies: a systematic review and meta-analysis. Blood Adv. *8*, 3555–3559. https://doi.org/10.1182/bloodadvances.2024012916.

14. Rodriguez-Otero, P., Usmani, S., Cohen, A.D., van de Donk, N.W.C.J., Leleu, X., Gállego Pérez-Larraya, J., Manier, S., Nooka, A.K., Mateos, M.V., Einsele, H., et al. (2024). International Myeloma Working Group immunotherapy committee consensus guidelines and recommendations for optimal use of T-cell-engaging bispecific antibodies in multiple myeloma. Lancet Oncol. *25*, e205–e216. https://doi.org/10.1016/S1470-2045(24)00043-3.

15. Lancman, G., Parsa, K., Rodriguez, C., Richter, J., Cho, H.J., Parekh, S., Richard, S., Rossi, A., Sanchez, L., Thibaud, S., et al. (2022). Infections and Severe Hypogammaglobulinemia in Multiple Myeloma Patients Treated with Anti-BCMA Bispecific Antibodies. Blood *140*, 10073–10074. https://doi.org/10.1182/blood-2022-163733.

16. Mohan, M., Nagavally, S., Dhakal, B., Radhakrishnan, S.V., Chhabra, S., D'Souza, A., and Hari, P. (2022). Risk of infections with B-cell maturation antigen-directed immunotherapy in multiple myeloma. Blood Adv. *6*, 2466–2470. https://doi.org/10.1182/bloodadvances.2021006178.

17. Philipp, N., Kazerani, M., Nicholls, A., Vick, B., Wulf, J., Straub, T., Scheurer, M., Muth, A., Hänel, G., Nixdorf, D., et al. (2022). T-cell exhaustion induced by continuous bispecific molecule exposure is ameliorated by treatment-free intervals. Blood *140*, 1104–1118. https://doi.org/10.1182/blood.2022015956.

18. Cordas Dos Santos, D.M., Tix, T., Shouval, R., Gafter-Gvili, A., Alberge, J.B., Cliff, E.R.S., Theurich, S., von Bergwelt-Baildon, M., Ghobrial, I.M., Subklewe, M., et al. (2024). A systematic review and meta-analysis of nonrelapse mortality after CAR T cell therapy. Nat. Med. *30*, 2667–2678. https://doi.org/10.1038/s41591-024-03084-6.

19. Lemoine, J., Bachy, E., Cartron, G., Beauvais, D., Gastinne, T., Di Blasi, R., Rubio, M.T., Guidez, S., Mohty, M., Casasnovas, R.O., et al. (2023). Non-Relapse Mortality after CAR T-Cell therapy for Large B-Cell Lymphoma: A LYSA Study from the DESCAR-T Registry. Blood Adv. *7*, 6589–6598. https://doi.org/10.1182/bloodadvances.2023010624.

20. Jain, M.D., Spiegel, J.Y., Nastoupil, L.J., Tamaresis, J., Ghobadi, A., Lin, Y., Lekakis, L., Reagan, P., Oluwole, O., McGuirk, J., et al. (2024). Five-Year Follow-Up of Standard-of-Care Axicabtagene Ciloleucel for Large B-Cell Lymphoma: Results From the US Lymphoma CAR T Consortium. J. Clin. Oncol. *42*, 3581–3592. https://doi.org/10.1200/JCO.23.02786.

21. Bahlis, N.J., Costello, C.L., Raje, N.S., Levy, M.Y., Dholaria, B., Solh, M., Tomasson, M.H., Damore, M.A., Jiang, S., Basu, C., et al. (2023). Elranatamab in relapsed or refractory multiple myeloma: the MagnetisMM-1 phase 1 trial. Nat. Med. *29*, 2570–2576. https://doi.org/10.1038/s41591-023-02589-w.

22. Bannerji, R., Arnason, J.E., Advani, R.H., Brown, J.R., Allan, J.N., Ansell, S.M., Barnes, J.A., O'Brien, S.M., Chávez, J.C., Duell, J., et al. (2022). Odronextamab, a human CD20xCD3 bispecific antibody in patients with CD20-positive B-cell malignancies (ELM-1): results from the relapsed or refractory non-Hodgkin lymphoma cohort in a single-arm, multicentre, phase 1 trial. Lancet Haematol. *9*, e327–e339. https://doi.org/10.1016/S2352-3026(22)00072-2.

23. Bartlett, N.L., Assouline, S., Giri, P., Schuster, S.J., Cheah, C.Y., Matasar, M., Gregory, G.P., Yoon, D.H., Shadman, M., Fay, K., et al. (2023). Mosunetuzumab monotherapy is active and tolerable in patients with relapsed/refractory diffuse large B-cell lymphoma. Blood Adv. *7*, 4926–4935. https://doi.org/10.1182/bloodadvances.2022009260.

24. Budde, L.E., Olszewski, A.J., Assouline, S., Lossos, I.S., Diefenbach, C., Kamdar, M., Ghosh, N., Modi, D., Sabry, W., Naik, S., et al. (2024). Mosunetuzumab with polatuzumab vedotin in relapsed or refractory aggressive large B cell lymphoma: a phase 1b/2 trial. Nat. Med. *30*, 229–239. https://doi.org/10.1038/s41591-023-02726-5.

25. Budde, L.E., Sehn, L.H., Matasar, M., Schuster, S.J., Assouline, S., Giri, P., Kuruvilla, J., Canales, M., Dietrich, S., Fay, K., et al. (2022). Safety and efficacy of mosunetuzumab, a bispecific antibody, in patients with relapsed or refractory follicular lymphoma: a single-arm, multicentre, phase 2 study. Lancet Oncol. *23*, 1055–1065. https://doi.org/10.1016/S1470-2045(22)00335-7.

26. Bumma, N., Richter, J., Jagannath, S., Lee, H.C., Hoffman, J.E., Suvannasankha, A., Zonder, J.A., Shah, M.R., Lentzsch, S., Baz, R., et al. (2024). Linvoseltamab for Treatment of Relapsed/Refractory Multiple Myeloma. J. Clin. Oncol. *42*, 2702–2712. https://doi.org/10.1200/JCO.24.01008.

27. Chari, A., Minnema, M.C., Berdeja, J.G., Oriol, A., van de Donk, N.W.C.J., Rodríguez-Otero, P., Askari, E., Mateos, M.V., Costa, L.J., Caers, J., et al. (2022). Talquetamab, a T-Cell-Redirecting GPRC5D Bispecific Antibody for Multiple Myeloma. N. Engl. J. Med. *387*, 2232–2244. https://doi.org/10.1056/NEJMoa2204591.

28. Coyle, L., Morley, N.J., Rambaldi, A., Mason, K.D., Verhoef, G., Furness, C.L., Zhang, A., Jung, A.S., Cohan, D., and Franklin, J.L. (2020). Open-Label, phase 2 study of blinatumomab as second salvage therapy in adults with relapsed/refractory aggressive B-cell non-Hodgkin lymphoma. Leuk. Lymphoma *61*, 2103–2112. https://doi.org/10.1080/10428194.2020.1759055.

29. D'Souza, A., Shah, N., Rodriguez, C., Voorhees, P.M., Weisel, K., Bueno, O.F., Pothacamury, R.K., Freise, K.J., Yue, S., Ross, J.A., et al. (2022). A Phase I First-in-Human Study of ABBV-383, a B-Cell Maturation Antigen x CD3 Bispecific T-Cell Redirecting Antibody, in Patients With Relapsed/Refractory Multiple Myeloma. J. Clin. Oncol. *40*, 3576–3586. https://doi.org/10.1200/JCO.22.01504.

30. Dickinson, M.J., Carlo-Stella, C., Morschhauser, F., Bachy, E., Corradini, P., Iacoboni, G., Khan, C., Wróbel, T., Offner, F., Trněný, M., et al. (2022). Glofitamab for Relapsed or Refractory Diffuse Large B-Cell Lymphoma. N. Engl. J. Med. *387*, 2220–2231. https://doi.org/10.1056/NEJMoa2206913.

31. Goebeler, M.E., Knop, S., Viardot, A., Kufer, P., Topp, M.S., Einsele, H., Noppeney, R., Hess, G., Kallert, S., Mackensen, A., et al. (2016). Bispecific T-Cell Engager (BiTE) Antibody Construct Blinatumomab for the Treatment of Patients With Relapsed/Refractory Non-Hodgkin Lymphoma: Final Results From a Phase I Study. J. Clin. Oncol. *34*, 1104–1111. https://doi.org/10.1200/JCO.2014.59.1586.

32. Guieze, R., Ysebaert, L., Roos-Weil, D., Fornecker, L.M., Ferrant, E., Molina, L., Aurran, T., Clavert, A., de Guibert, S., Michallet, A.S., et al. (2024). Blinatumomab after R-CHOP bridging therapy for patients with Richter transformation: a phase 2 multicentre trial. Nat. Commun. *15*, 6822. https://doi.org/10.1038/s41467-024-51264-2.

33. Hutchings, M., Morschhauser, F., Iacoboni, G., Carlo-Stella, C., Offner, F.C., Sureda, A., Salles, G., Martínez-Lopez, J., Crump, M., Thomas, D.N., et al. (2021). Glofitamab, a Novel, Bivalent CD20-Targeting T-Cell-Engaging Bispecific Antibody, Induces Durable Complete Remissions in Relapsed or Refractory B-Cell Lymphoma: A Phase I Trial. J. Clin. Oncol. *39*, 1959–1970. https://doi.org/10.1200/JCO.20.03175.

34. Izutsu, K., Kumode, T., Yuda, J., Nagai, H., Mishima, Y., Suehiro, Y., Yamamoto, K., Fujisaki, T., Ishitsuka, K., Ishizawa, K., et al. (2023). Subcutaneous epcoritamab monotherapy in Japanese adults with relapsed/refractory diffuse large B-cell lymphoma. Cancer Sci. *114*, 4643–4653. https://doi.org/10.1111/cas.15996.

35. Katz, D.A., Morris, J.D., Chu, M.P., David, K.A., Thieblemont, C., Morley, N.J., Khan, S.S., Viardot, A., Martín García-Sancho, A., Rodríguez-García, G., et al. (2022). Open-label, phase 2 study of blinatumomab after frontline R-chemotherapy in adults with newly diagnosed, high-risk DLBCL. Leuk. Lymphoma *63*, 2063–2073. https://doi.org/10.1080/10428194.2022.2064981.

36. Kim, T.M., Taszner, M., Novelli, S., Cho, S.G., Villasboas, J.C., Merli, M., Jiménez-Ubieto, A., Tessoulin, B., Poon, L.M., Tucker, D., et al. (2024). Safety and efficacy of odronextamab in patients with relapsed or refractory follicular lymphoma. Ann. Oncol. *35*, 1039–1047. https://doi.org/10.1016/j.annonc.2024.08.2239.

37. Lesokhin, A.M., Tomasson, M.H., Arnulf, B., Bahlis, N.J., Miles Prince, H., Niesvizky, R., Rodríguez-Otero, P., Martinez-Lopez, J., Koehne, G., Touzeau, C., et al. (2023). Elranatamab in relapsed or refractory multiple myeloma: phase 2 MagnetisMM-3 trial results. Nat. Med. *29*, 2259–2267. https://doi.org/10.1038/s41591-023-02528-9.

38. Linton, K.M., Vitolo, U., Jurczak, W., Lugtenburg, P.J., Gyan, E., Sureda, A., Christensen, J.H., Hess, B., Tilly, H., Cordoba, R., et al. (2024). Epcoritamab monotherapy in patients with relapsed or refractory follicular lymphoma (EPCORE NHL-1): a phase 2 cohort of a single-arm, multicentre study. Lancet Haematol. *11*, e593–e605. https://doi.org/10.1016/S2352-3026(24)00166-2.

39. Moreau, P., Garfall, A.L., van de Donk, N.W.C.J., Nahi, H., San-Miguel, J.F., Oriol, A., Nooka, A.K., Martin, T., Rosinol, L., Chari, A., et al. (2022). Teclistamab in Relapsed or Refractory Multiple Myeloma. N. Engl. J. Med. *387*, 495–505. https://doi.org/10.1056/NEJMoa2203478.

40. Olszewski, A.J., Phillips, T.J., Hoffmann, M.S., Armand, P., Kim, T.M., Yoon, D.H., Mehta, A., Greil, R., Westin, J., Lossos, I.S., et al. (2023). Mosunetuzumab in combination with CHOP in previously untreated DLBCL: safety and efficacy results from a phase 2 study. Blood Adv. *7*, 6055–6065. https://doi.org/10.1182/bloodadvances.2023010840.

41. Phillips, T.J., Carlo-Stella, C., Morschhauser, F., Bachy, E., Crump, M., Trněný, M., Bartlett, N.L., Zaucha, J., Wrobel, T., Offner, F., et al. (2025). Glofitamab in Relapsed/Refractory Mantle Cell Lymphoma: Results From a Phase I/II Study. J. Clin. Oncol. *43*, 318–328. https://doi.org/10.1200/JCO.23.02470.

42. Song, Y.Q., Zhang, H.L., Huang, H.Q., Zhang, Q.Y., Jing, H.M., Wang, C., Wu, C., Li, D.H., Dai, Y., Humphrey, K., and Zhu, J. (2024). Glofitamab monotherapy induces high complete response rates and manageable safety in Chinese patients with heavily pretreated relapsed or refractory diffuse large B-cell lymphoma. Haematologica *109*, 1269–1273. https://doi.org/10.3324/haematol.2023.283802.

43. Thieblemont, C., Phillips, T., Ghesquieres, H., Cheah, C.Y., Clausen, M.R., Cunningham, D., Do, Y.R., Feldman, T., Gasiorowski, R., Jurczak, W., et al. (2023). Epcoritamab, a Novel, Subcutaneous CD3xCD20 Bispecific T-Cell-Engaging Antibody, in Relapsed or Refractory Large B-Cell Lymphoma: Dose Expansion in a Phase I/II Trial. J. Clin. Oncol. *41*, 2238–2247. https://doi.org/10.1200/JCO.22.01725.

44. Viardot, A., Goebeler, M.E., Hess, G., Neumann, S., Pfreundschuh, M., Adrian, N., Zettl, F., Libicher, M., Sayehli, C., Stieglmaier, J., et al. (2016). Phase 2 study of the bispecific T-cell engager (BiTE) antibody blinatumomab in relapsed/refractory diffuse large B-cell lymphoma. Blood *127*, 1410–1416. https://doi.org/10.1182/blood-2015-06-651380.

45. Birtas Atesoglu, E., Gulbas, Z., Uzay, A., Ozcan, M., Ozkalemkas, F., Dal, M.S., Kalyon, H., Akay, O.M., Deveci, B., Bekoz, H., et al. (2023). Glofitamab in relapsed/refractory diffuse large B-cell lymphoma: Real-world data. Hematol. Oncol. *41*, 663–673. https://doi.org/10.1002/hon.3174.

46. Dima, D., Davis, J.A., Ahmed, N., Jia, X., Sannareddy, A., Shaikh, H., Shune, L., Kaur, G., Khouri, J., Afrough, A., et al. (2024). Safety and Efficacy of Teclistamab in Patients with Relapsed/Refractory Multiple Myeloma: A Real-World Experience. Transpl. Cell. Ther. *30*, 308.e1–308.e13. https://doi.org/10.1016/j.jtct.2023.12.016.

47. Hsu, Y.T., Wu, S.J., Kao, H.W., Hsiao, S.Y., Liao, C.K., Chen, T.Y., and Wang, M.C. (2024). Glofitamab as a salvage treatment for B-cell lymphomas in the real world: A multicenter study in Taiwan. Cancer *130*, 1972–1981. https://doi.org/10.1002/cncr.35217.

48. Lebreton, P., Lachenal, F., Bouillie, S., Pica, G.M., Aftisse, H., Pascal, L., Montes, L., Macro, M., Johnson, N., Harel, S., et al. (2024). Teclistamab for relapsed refractory multiple myeloma patients on dialysis. Br. J. Haematol. *205*, 2077–2079. https://doi.org/10.1111/bjh.19772.

49. Crowther, M., Lim, W., and Crowther, M.A. (2010). Systematic review and meta-analysis methodology. Blood *116*, 3140–3146. https://doi.org/10.1182/blood-2010-05-280883.

50. Crombie, J.L., Graff, T., Falchi, L., Karimi, Y.H., Bannerji, R., Nastoupil, L., Thieblemont, C., Ursu, R., Bartlett, N., Nachar, V., et al. (2024). Consensus recommendations on the management of toxicity associated with CD3×CD20 bispecific antibody therapy. Blood *143*, 1565–1575. https://doi.org/10.1182/blood.2023022432.

51. Hill, B.T., Rybicki, L., Bolwell, B.J., Smith, S., Dean, R., Kalaycio, M., Pohlman, B., Tench, S., Sobecks, R., Andresen, S., et al. (2011). The non-relapse mortality rate for patients with diffuse large B-cell lymphoma is greater than relapse mortality 8 years after autologous stem cell transplantation and is significantly higher than mortality rates of population controls. Br. J. Haematol. *152*, 561–569. https://doi.org/10.1111/j.1365-2141.2010.08549.x.

52. Bishop, M.R., Dickinson, M., Purtill, D., Barba, P., Santoro, A., Hamad, N., Kato, K., Sureda, A., Greil, R., Thieblemont, C., et al. (2022). Second-Line Tisagenlecleucel or Standard Care in Aggressive B-Cell Lymphoma. N. Engl. J. Med. *386*, 629–639. https://doi.org/10.1056/NEJMoa2116596.

53. Abramson, J.S., Solomon, S.R., Arnason, J., Johnston, P.B., Glass, B., Bachanova, V., Ibrahimi, S., Mielke, S., Mutsaers, P., Hernandez-Ilizaliturri, F., et al. (2023). Lisocabtagene maraleucel as second-line therapy for large B-cell lymphoma: primary analysis of the phase 3 TRANSFORM study. Blood *141*, 1675–1684. https://doi.org/10.1182/blood.2022018730.

54. Locke, F.L., Miklos, D.B., Jacobson, C.A., Perales, M.A., Kersten, M.J., Oluwole, O. O., Ghobadi, A., Rapoport, A.P., McGuirk, J., Pagel, J.M., et al. (2022). Axicabtagene Ciloleucel as Second-Line Therapy for Large B-Cell Lymphoma. N. Engl. J. Med. *386*, 640–654. https://doi.org/10.1056/NEJMoa2116133.

55. Rodriguez-Otero, P., Ailawadhi, S., Arnulf, B., Patel, K., Cavo, M., Nooka, A.K., Manier, S., Callander, N., Costa, L.J., Vij, R., et al. (2023). Ide-cel or Standard Regimens in Relapsed and Refractory Multiple Myeloma. N. Engl. J. Med. *388*, 1002–1014. https://doi.org/10.1056/NEJMoa2213614.

56. Dreger, P., Sureda, A., Ahn, K.W., Eapen, M., Litovich, C., Finel, H., Boumendil, A., Gopal, A., Herrera, A.F., Schmid, C., et al. (2019). PTCy-based haploidentical vs matched related or unrelated donor reduced-intensity conditioning transplant for DLBCL. Blood Adv. *3*, 360–369. https://doi.org/10.1182/bloodadvances.2018027748.

57. Hamadani, M., Gopal, A.K., Pasquini, M., Kim, S., Qiu, X., Ahmed, S., Lazaryan, A., Bhatt, V.R., Daly, A., Lulla, P., et al. (2022). Allogeneic transplant and CAR-T therapy after autologous transplant failure in DLBCL: a noncomparative cohort analysis. Blood Adv. *6*, 486–494. https://doi.org/10.1182/bloodadvances.2021005788.

58. Robinson, S.P., Goldstone, A.H., Mackinnon, S., Carella, A., Russell, N., de Elvira, C. R., Taghipour, G., Schmitz, N.; Lymphoma Working Party of the European Group for Blood and Bone Marrow Transplantation, and Bone Marrow, T. (2002). Chemoresistant or aggressive lymphoma predicts for a poor outcome following reduced-intensity allogeneic progenitor cell transplantation: an analysis from the Lymphoma Working Party of the European Group for Blood and Bone Marrow Transplantation. Blood *100*, 4310–4316. https://doi.org/10.1182/blood-2001-11-0107.

59. Lin, S.Y., Lu, K.J., Zheng, X.N., Hou, J., and Liu, T.T. (2024). Efficacy and survival outcome of allogeneic stem-cell transplantation in multiple myeloma: meta-analysis in the recent 10 years. Front. Oncol. *14*, 1341631. https://doi.org/10.3389/fonc.2024.1341631.

60. Crochet, G., Iacoboni, G., Couturier, A., Bachy, E., Iraola-Truchuelo, J., Gastinne, T., Cartron, G., Fradon, T., Lesne, B., Kwon, M., et al. (2024). Efficacy of CAR T-cell therapy is not impaired by previous bispecific antibody treatment in large B-cell lymphoma. Blood *144*, 334–338. https://doi.org/10.1182/blood.2024024526.

61. Haydu, J.E., and Abramson, J.S. (2024). The rules of T-cell engagement: current state of CAR T cells and bispecific antibodies in B-cell lymphomas. Blood Adv. *8*, 4700–4710. https://doi.org/10.1182/bloodadvances.2021004535.

62. Abramson, J.S., Ku, M., Hertzberg, M., Huang, H.Q., Fox, C.P., Zhang, H., Yoon, D. H., Kim, W.S., Abdulhaq, H., Townsend, W., et al. (2024). Glofitamab plus gemcitabine and oxaliplatin (GemOx) versus rituximab-GemOx for relapsed or refractory diffuse large B-cell lymphoma (STARGLO): a global phase 3, randomised, open-label trial. Lancet *404*, 1940–1954. https://doi.org/10.1016/S0140-6736(24)01774-4.

63. Garfall, A.L., Nooka, A.K., van de Donk, N.W.C.J., Moreau, P., Bhutani, M., Oriol, A., Martin, T.G., Rosiñol, L., Mateos, M.-V., Bahlis, N.J., et al. (2024). Long-term follow-up from the phase 1/2 MajesTEC-1 trial of teclistamab in patients with relapsed/refractory multiple myeloma. J. Clin. Oncol. *42*, 7540. https://doi.org/10.1200/JCO.2024.42.16_suppl.7540.

64. Raje, N., Anderson, K., Einsele, H., Efebera, Y., Gay, F., Hammond, S.P., Lesokhin, A.M., Lonial, S., Ludwig, H., Moreau, P., et al. (2023). Monitoring, prophylaxis, and treatment of infections in patients with MM receiving bispecific antibody therapy: consensus recommendations from an expert panel. Blood Cancer J. *13*, 116. https://doi.org/10.1038/s41408-023-00879-7.

65. Kampouri, E., Little, J.S., Rejeski, K., Manuel, O., Hammond, S.P., and Hill, J.A. (2023). Infections after chimeric antigen receptor (CAR)-T-cell therapy for hematologic malignancies. Transpl. Infect. Dis. *25*, e14157. https://doi.org/10.1111/tid.14157.

66. Logue, J.M., Zucchetti, E., Bachmeier, C.A., Krivenko, G.S., Larson, V., Ninh, D., Grillo, G., Cao, B., Kim, J., Chavez, J.C., et al. (2021). Immune reconstitution and associated infections following axicabtagene ciloleucel in relapsed or refractory large B-cell lymphoma. Haematologica *106*, 978–986. https://doi.org/10.3324/haematol.2019.238634.

67. Logue, J.M., Peres, L.C., Hashmi, H., Colin-Leitzinger, C.M., Shrewsbury, A.M., Hosoya, H., Gonzalez, R.M., Copponex, C., Kottra, K.H., Hovanky, V., et al. (2022). Early cytopenias and infections after standard of care idecabtagene vicleucel in relapsed or refractory multiple myeloma. Blood Adv. *6*, 6109–6119. https://doi.org/10.1182/bloodadvances.2022008320.

68. Lancman, G., Parsa, K., Kotlarz, K., Avery, L., Lurie, A., Lieberman-Cribbin, A., Cho, H.J., Parekh, S.S., Richard, S., Richter, J., et al. (2023). IVIg Use Associated with Ten-Fold Reduction of Serious Infections in Multiple Myeloma Patients Treated with Anti-BCMA Bispecific Antibodies. Blood Cancer Discov. *4*, 440–451. https://doi.org/10.1158/2643-3230.Bcd-23-0049.

69. Cohen, Y.C., Magen, H., Gatt, M., Sebag, M., Kim, K., Min, C.K., Ocio, E.M., Yoon, S.S., Chu, M.P., Rodríguez-Otero, P., et al. (2025). Talquetamab plus Teclistamab in Relapsed or Refractory Multiple Myeloma. N. Engl. J. Med. *392*, 138–149. https://doi.org/10.1056/NEJMoa2406536.

70. Ludwig, H., Terpos, E., van de Donk, N., Mateos, M.-V., Moreau, P., Dimopoulos, M.-A., Delforge, M., Rodriguez-Otero, P., San-Miguel, J., Yong, K., et al. (2023). Prevention and management of adverse events during treatment with bispecific antibodies and CAR T cells in multiple myeloma: a consensus report of the European Myeloma Network. Lancet Oncol. *24*, e255–e269. https://doi.org/10.1016/S1470-2045(23)00159-6.

71. Fuerst, D., Frank, S., Mueller, C., Beelen, D.W., Schetelig, J., Niederwieser, D., Finke, J., Bunjes, D., Kröger, N., Neuchel, C., et al. (2018). Competing-risk outcomes after hematopoietic stem cell transplantation from the perspective of time-dependent effects. Haematologica *103*, 1527–1534. https://doi.org/10.3324/haematol.2017.183012.

72. DeFilipp, Z., Alousi, A.M., Pidala, J.A., Carpenter, P.A., Onstad, L.E., Arai, S., Arora, M., Cutler, C.S., Flowers, M.E.D., Kitko, C.L., et al. (2021). Nonrelapse mortality among patients diagnosed with chronic GVHD: an updated analysis from the Chronic GVHD Consortium. Blood Adv. *5*, 4278–4284. https://doi.org/10.1182/bloodadvances.2021004941.

73. Andersen, P.K., Geskus, R.B., de Witte, T., and Putter, H. (2012). Competing risks in epidemiology: possibilities and pitfalls. Int. J. Epidemiol. *41*, 861–870. https://doi.org/10.1093/ije/dyr213.

74. Hines, M.R., Knight, T.E., McNerney, K.O., Leick, M.B., Jain, T., Ahmed, S., Frigault, M.J., Hill, J.A., Jain, M.D., Johnson, W.T., et al. (2023). Immune Effector Cell associated Hemophagocytic Lymphohistiocytosis-like Syndrome (IEC-HS). Transpl. Cell. Ther. *29*, 438.e1–438.e16. https://doi.org/10.1016/j.jtct.2023.03.006.

75. Rejeski, K., Subklewe, M., Aljurf, M., Bachy, E., Balduzzi, A., Barba, P., Bruno, B., Benjamin, R., Carrabba, M.G., Chabannon, C., et al. (2023). Immune effector cell-associated hematotoxicity: EHA/EBMT consensus grading and best practice recommendations. Blood *142*, 865–877. https://doi.org/10.1182/blood.2023020578.

76. Rejeski, K., Greco, R., Onida, F., Sánchez-Ortega, I., Bonini, C., Sureda, A., Gribben, J.G., Yakoub-Agha, I., and Subklewe, M. (2023). An International Survey on Grading, Diagnosis, and Management of Immune Effector Cell-Associated Hematotoxicity (ICAHT) Following CAR T-cell Therapy on Behalf of the EBMT and EHA. Hemasphere *7*, e889. https://doi.org/10.1097/HS9.0000000000000889.

77. Rejeski, K., Wang, Y., Hansen, D.K., Iacoboni, G., Bachy, E., Bansal, R., Penack, O., Müller, F., Bethge, W., Munoz, J., et al. (2024). Applying the EHA/EBMT grading for ICAHT after CAR-T: comparative incidence and association with infections and mortality. Blood Adv. *8*, 1857–1868. https://doi.org/10.1182/bloodadvances.2023011767.

78. Fortuna, G.G., Banerjee, R., Savid-Frontera, C., Song, J., Morán-Segura, C.M., Nguyen, J.V., Lekakis, L., Fernandez-Pol, S., Samraj, A.N., Naresh, K.N., et al. (2024/10/16 2024). Immune effector cell-associated enterocolitis following chimeric antigen receptor T-cell therapy in multiple myeloma. Blood Cancer J. *14*, 180. https://doi.org/10.1038/s41408-024-01167-8.

79. Page, M.J., McKenzie, J.E., Bossuyt, P.M., Boutron, I., Hoffmann, T.C., Mulrow, C. D., Shamseer, L., Tetzlaff, J.M., Akl, E.A., and Brennan, S.E. (2021). PRISMA 2020 Statement: Updated Guideline Reporting Systematic Reviews. Syst. Rev. *10*, 1–11.

80. Munn, Z., Barker, T.H., Moola, S., Tufanaru, C., Stern, C., McArthur, A., Stephenson, M., and Aromataris, E. (2020). Methodological quality of case series studies: an introduction to the JBI critical appraisal tool. JBI Evid. Synth. *18*, 2127–2133.

81. Easterbrook, P.J., Berlin, J.A., Gopalan, R., and Matthews, D.R. (1991). Publication bias in clinical research. The Lancet *337*, 867–872.

82. Schwarzer, G. (2007). meta: An R package for meta-analysis. R. News *7*, 40–45.

83. Newcombe, R.G. (1998). Two-sided confidence intervals for the single proportion: comparison of seven methods. Stat. Med. *17*, 857–872.

84. Higgins, J.P.T., Thompson, S.G., Deeks, J.J., and Altman, D.G. (2003). Measuring inconsistency in meta-analyses. BMJ *327*, 557–560. https://doi.org/10.1136/bmj.327.7414.557.

85. Hardy, R.J., and Thompson, S.G. (1996). A likelihood approach to meta-analysis with random effects. Stat. Med. *15*, 619–629.

86. Knapp, G., and Hartung, J. (2003). Improved tests for a random effects meta-regression with a single covariate. Stat. Med. *22*, 2693–2710. https://doi.org/10.1002/sim.1482.

87. Higgins, J.P.T., and Thompson, S.G. (2004). Controlling the risk of spurious findings from meta-regression. Stat. Med. *23*, 1663–1682. https://doi.org/10.1002/sim.1752.

88. Higgins, J., Thomas, J., Chandler, J., Cumpston, M., Li, T., Page, M., and Welch, V. (2023). Cochrane Handbook for Systematic Reviews of Interventions Version 6.4 (Updated August 2023) (Cochrane). www.training.cochrane.org/handbook.

YMTHE, Volume *33*

Supplemental Information

Non-relapse mortality with bispecific antibodies: A systematic review and meta-analysis in lymphoma and multiple myeloma

Tobias Tix, Mohammad Alhomoud, Roni Shouval, Gloria Iacoboni, Edward R. Scheffer Cliff, Doris K. Hansen, Saad Z. Usmani, Gilles Salles, Miguel-Angel Perales, David M. Cordas dos Santos, and Kai Rejeski

Study	Events	Time	Proportion	95%-CI
Atesoglu_2023	5	20.425	0.245	[0.102; 0.588]
Bahlis_2023	5	55.000	0.091	[0.038; 0.218]
Bannerji_2022	7	50.750	0.138	[0.066; 0.289]
Bartlett_2023	3	74.067	0.041	[0.013; 0.126]
Budde_2022	1	137.250	0.007	[0.001; 0.052]
Budde_2024_A1	2	221.100	0.009	[0.002; 0.036]
Budde_2024_A2	8	438.600	0.018	[0.009; 0.036]
Budde_2024_B	3	195.183	0.015	[0.005; 0.048]
Bumma_2024	6	9.750	0.615	[0.276; 1.370]
Chari_2022_A	0	29.250	0.000	[0.001; 0.273]
Chari_2022_B	3	15.400	0.195	[0.063; 0.604]
Coyle_2020	3	16.742	0.179	[0.058; 0.556]
D'Souza_2022	1	34.850	0.029	[0.004; 0.204]
Dickinson_2022	8	161.700	0.049	[0.025; 0.099]
Dima_2024	3	33.567	0.089	[0.029; 0.277]
Guieze_2024	0	48.333	0.000	[0.001; 0.165]
Hsu_2024	3	41.075	0.073	[0.024; 0.226]
Hutchings_2021	2	192.375	0.010	[0.003; 0.042]
Izutsu_2023	0	25.200	0.000	[0.001; 0.317]
Katz_2022	1	28.000	0.036	[0.005; 0.254]
Kim_2024	18	214.400	0.084	[0.053; 0.133]
Lebreton_2024	1	6.750	0.148	[0.021; 1.052]
Lesokhin_2023	14	150.675	0.093	[0.055; 0.157]
Linton_2024_A	12	185.600	0.065	[0.037; 0.114]
Linton_2024_B	0	40.850	0.000	[0.001; 0.196]
Moreau_2022	19	193.875	0.098	[0.063; 0.154]
Olszewski_2023	1	106.667	0.009	[0.001; 0.067]
Philipps_2024	9	98.000	0.092	[0.048; 0.177]
Song_2024	0	33.750	0.000	[0.001; 0.237]
Thieblemont_2022	9	139.992	0.064	[0.033; 0.124]
Viardot_2016	1	22.425	0.045	[0.006; 0.317]
**Random effects model**	**148**		**0.043**	**[0.028; 0.067]**

Heterogeneity: $I^2 = 69.8\%$, $p < 0.001$

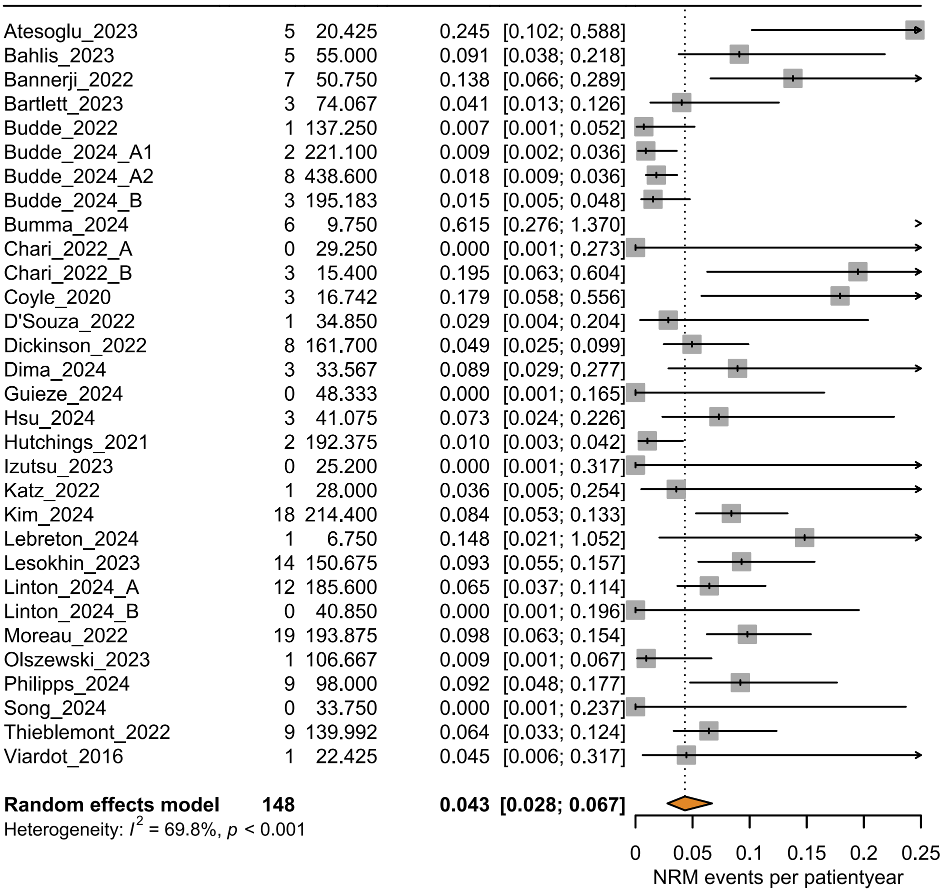

Figure S1. Forest plot of NRM per patient-year. Forest plot illustrating NRM per patient year and 95% confidence intervals (95% CI). The random effects model results and heterogeneity measures are depicted. One study was excluded from analysis since no median follow-up was reported (Goebeler et al, 2016, J Clin Oncol).

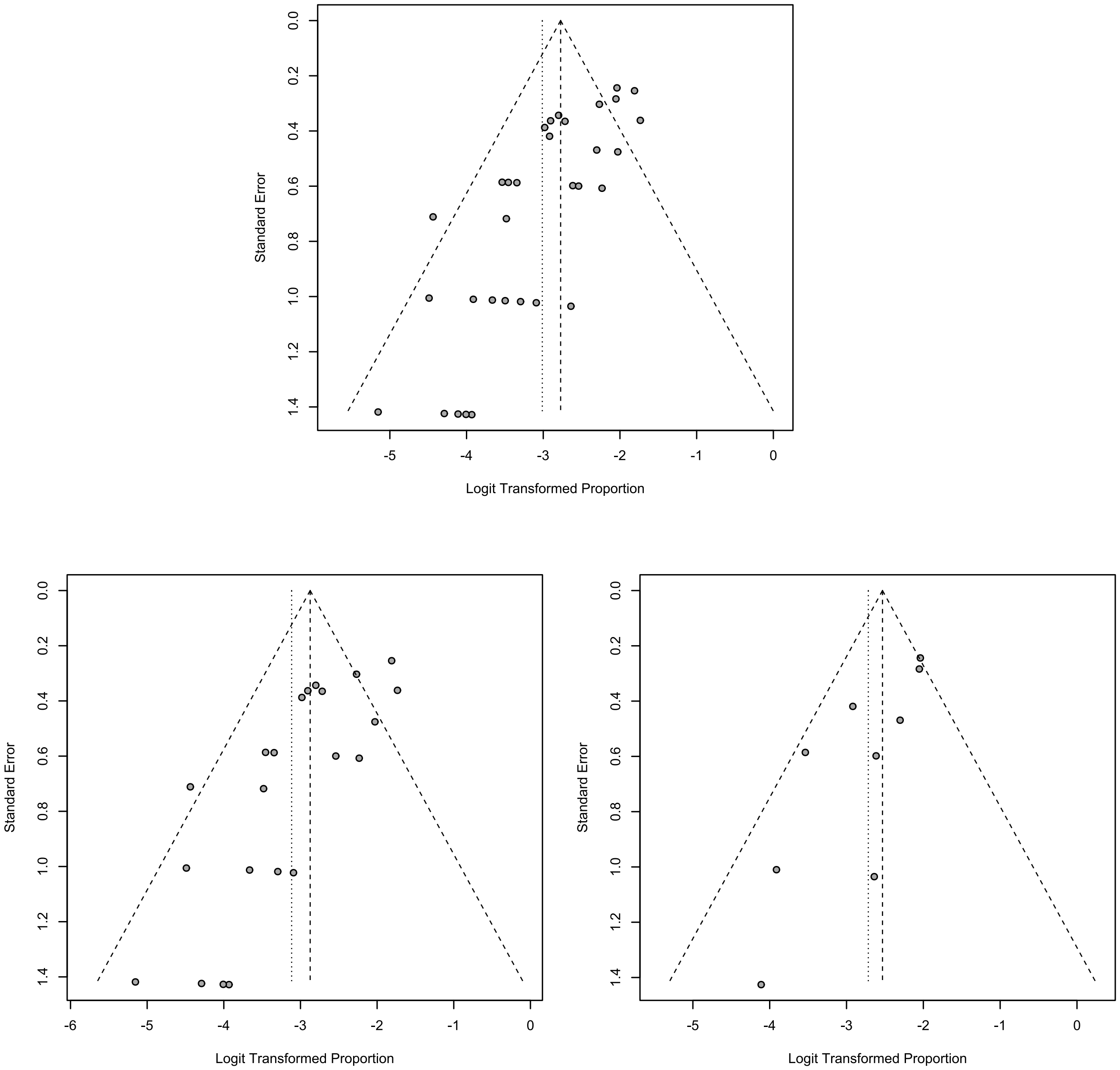

Figure S2. Funnel plots to assess reporting bias. Reporting bias was analyzed by funnel plots. P-values for funnel plot asymmetry were derived from Egger regression tests.

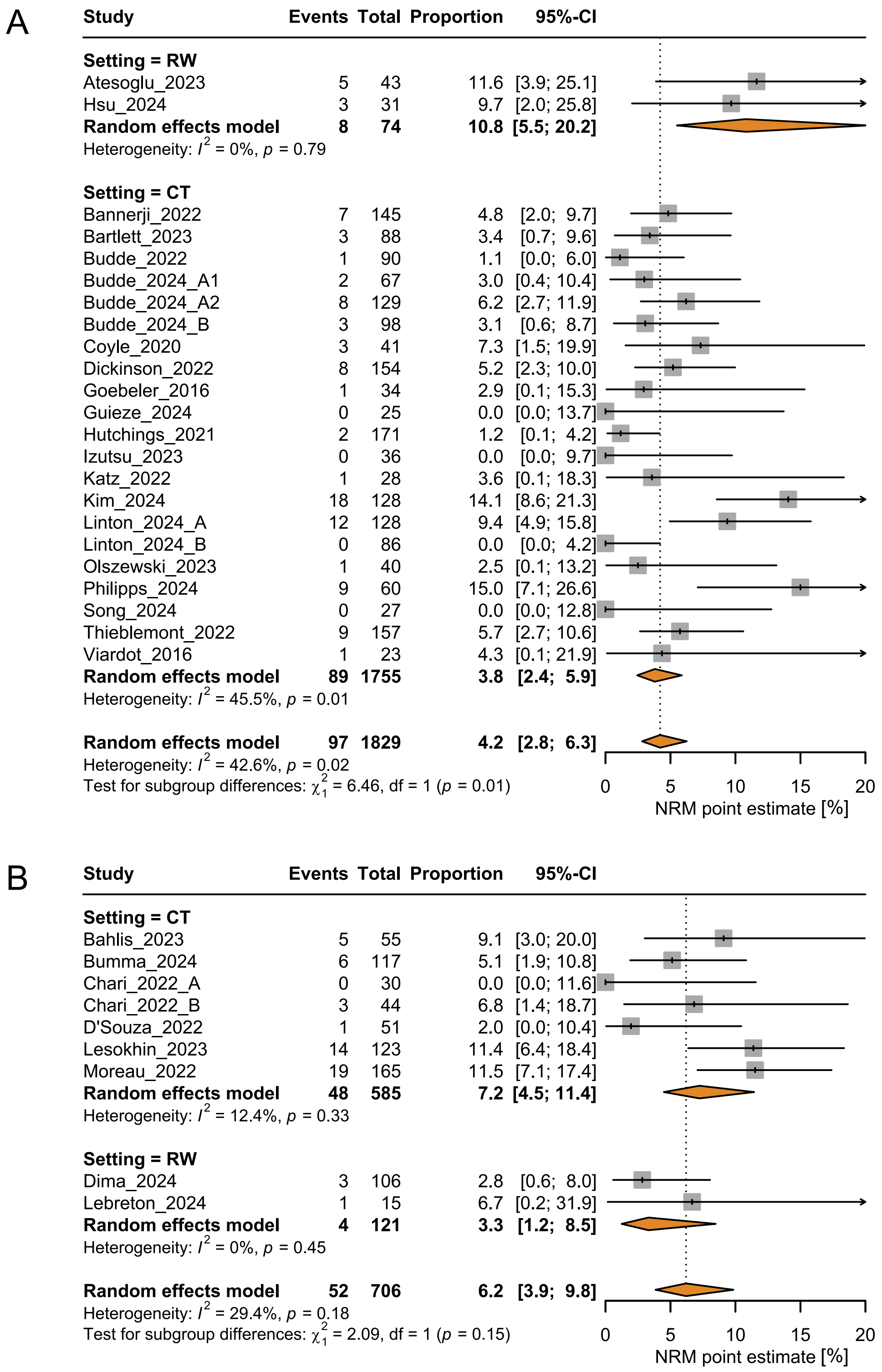

Figure S3. Subgroup analysis of NRM point estimates stratified by treatment setting. Forest plot illustrating NRM point estimates and 95% confidence intervals (95% CI) stratified by the treatment setting comparing real-world studies and clinical trials for B-Non-Hodgkin Lymphoma (A) and multiple myeloma (B). The random effects model results and heterogeneity measures are depicted for each treatment setting. CT = clinical trial, RW = real-world study.

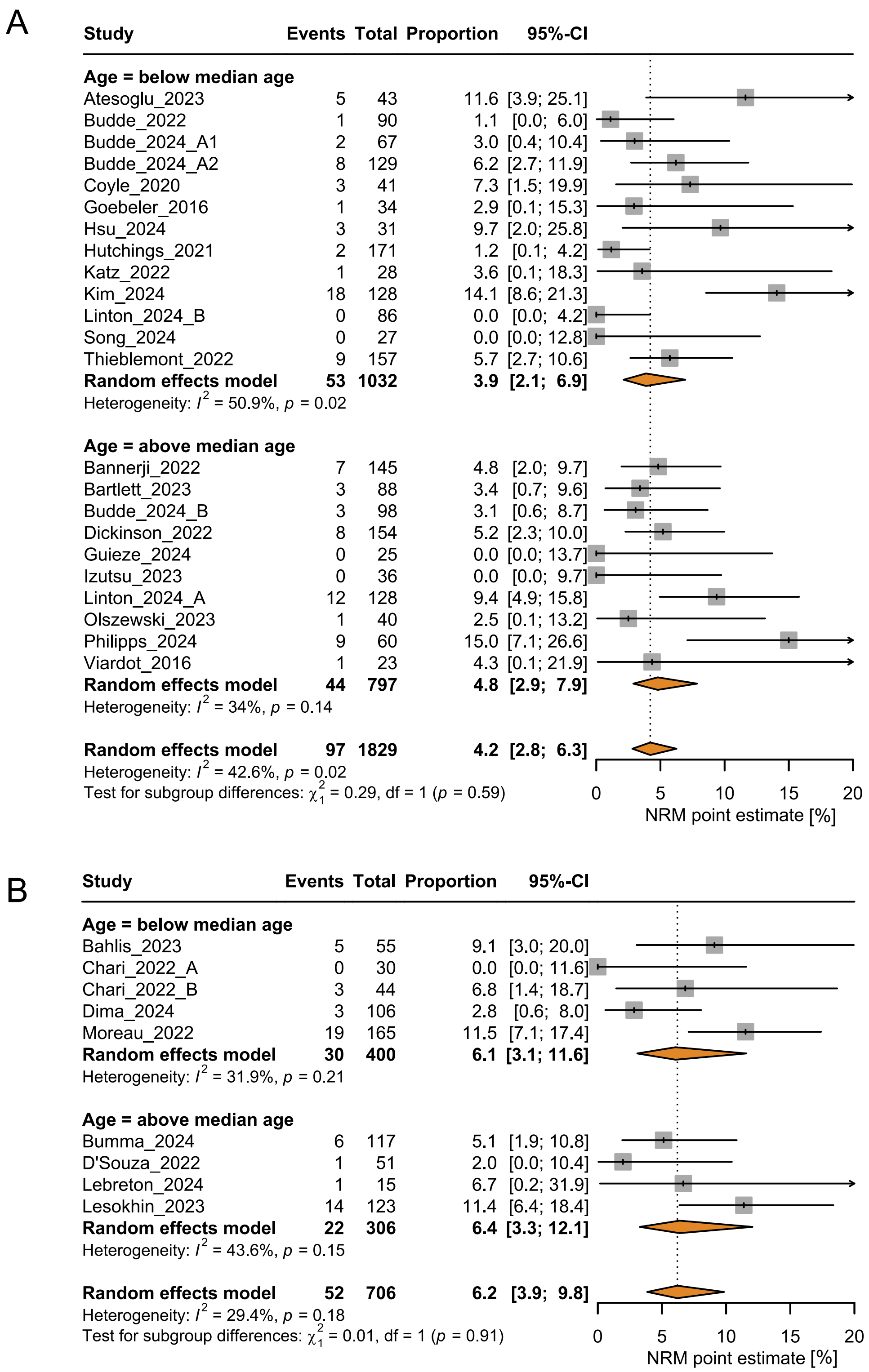

Figure S4. Subgroup analysis of NRM point estimates stratified by median age. Forest plot illustrating NRM point estimates and 95% confidence intervals (95% CI) stratified by the median age of each study cohort for B-Non-Hodgkin Lymphoma (A) and multiple myeloma (B). The random effects model results and heterogeneity measures are depicted for each disease entity. NRM = non-relapse mortality.

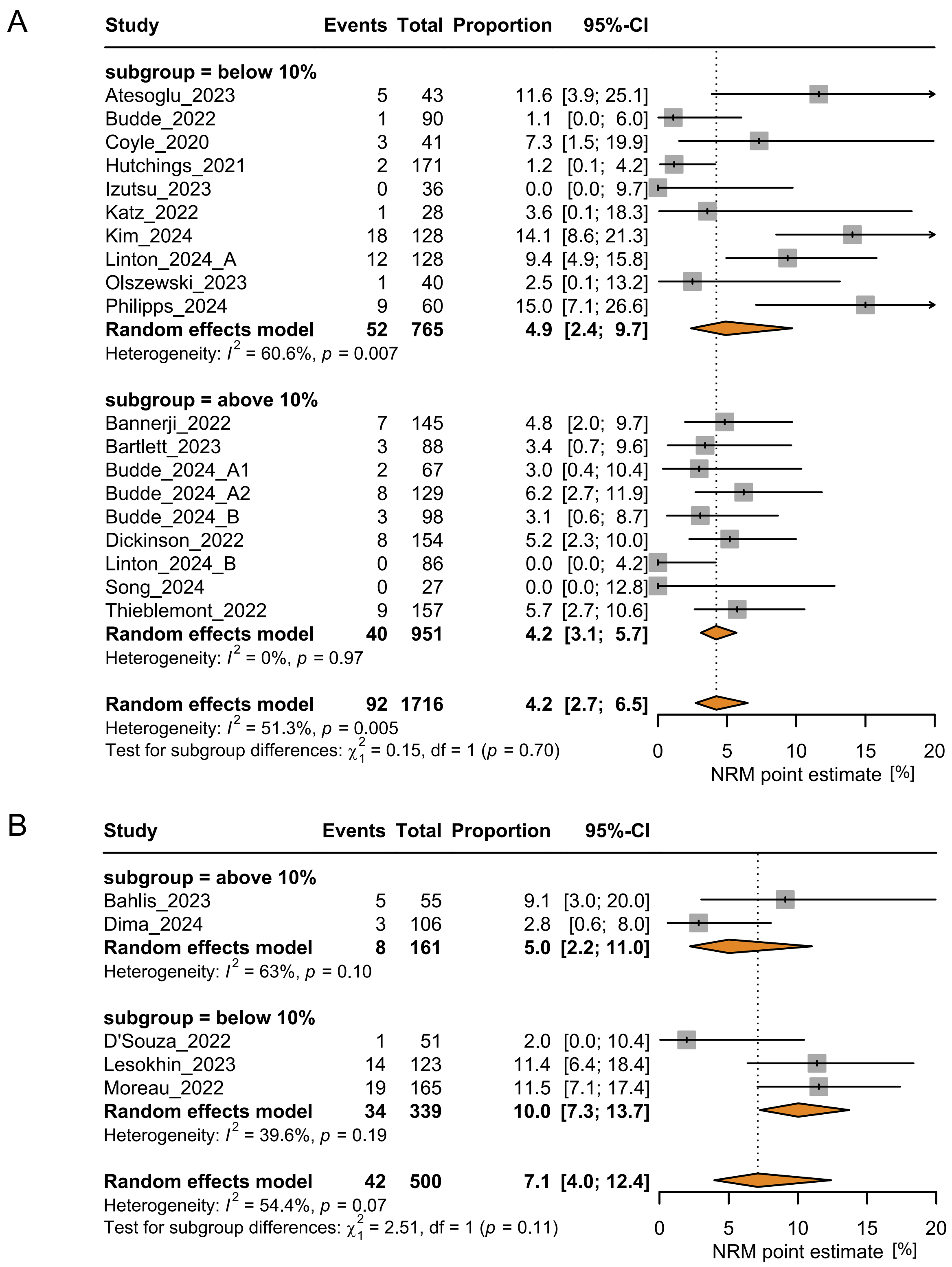

Figure S5. Subgroup analysis of NRM point estimates stratified by prior CAR-T exposure. Forest plot illustrating NRM point estimates and 95% confidence intervals (95% CI) stratified by prior CAR-T cell exposure of above or below 10% of the patients in each study cohort for B-Non-Hodgkin Lymphoma (A) and multiple myeloma (B). Only studies reporting prior CAR-T exposure were included in this analysis. The random effects model results and heterogeneity measures are depicted for cohorts with prior CAR-T exposure below and above 10%. Studies not reporting prior CAR-T exposure were excluded from analysis (Bumma et al, 2024, J Clin Oncol; Chari et al, 2022, N Engl J Med; Goebeler et al, 2016, J Clin Oncol; Guieze et al, 2024, Nat Commun; Viardot et al, 2016, Blood; Dima et al, 2024, Transplant Cell Ther; Hsu et al, 2024, Cancer). NRM = non-relapse mortality.

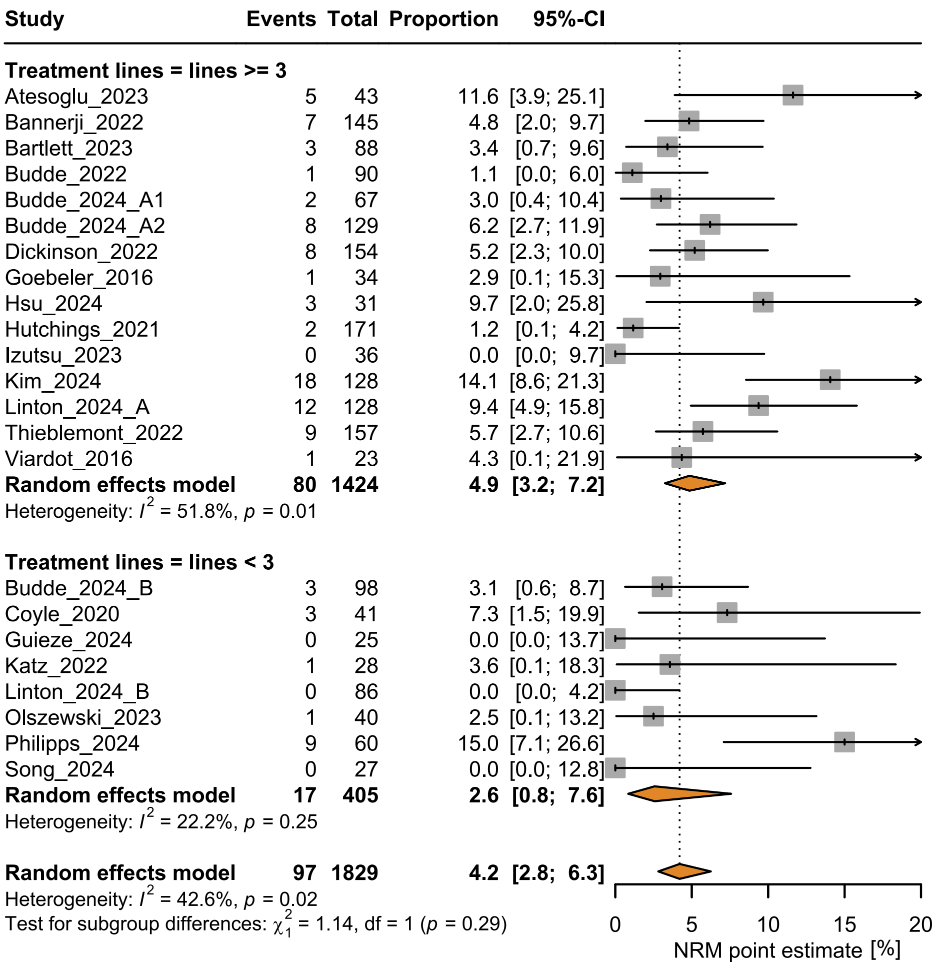

Figure S6. Subgroup analysis of NRM point estimates stratified by prior treatment lines within lymphoma cohorts. Forest plot illustrating NRM point estimates and 95% confidence intervals (95% CI) stratified by the median number of prior treatment lines among the lymphoma cohorts. The random effects model results and heterogeneity measures are depicted across cohorts with below and above three prior treatment lines. NRM = non-relapse mortality.

A

Study	Events	Total	Proportion	95%-CI
**Response = ORR < 56**				
Atesoglu_2023	5	43	11.6	[3.9; 25.1]
Bannerji_2022	7	145	4.8	[2.0; 9.7]
Bartlett_2023	3	88	3.4	[0.7; 9.6]
Budde_2024_A2	8	129	6.2	[2.7; 11.9]
Coyle_2020	3	41	7.3	[1.5; 19.9]
Dickinson_2022	8	154	5.2	[2.3; 10.0]
Guieze_2024	0	25	0.0	[0.0; 13.7]
Hutchings_2021	2	171	1.2	[0.1; 4.2]
Izutsu_2023	0	36	0.0	[0.0; 9.7]
Viardot_2016	1	23	4.3	[0.1; 21.9]
**Random effects model**	**37**	**855**	**4.2**	**[2.7; 6.3]**
Heterogeneity: I^2 = 2.7%, p = 0.41				
**Response = ORR >= 56**				
Budde_2022	1	90	1.1	[0.0; 6.0]
Budde_2024_A1	2	67	3.0	[0.4; 10.4]
Budde_2024_B	3	98	3.1	[0.6; 8.7]
Hsu_2024	3	31	9.7	[2.0; 25.8]
Katz_2022	1	28	3.6	[0.1; 18.3]
Linton_2024_A	12	128	9.4	[4.9; 15.8]
Linton_2024_B	0	86	0.0	[0.0; 4.2]
Olszewski_2023	1	40	2.5	[0.1; 13.2]
Philipps_2024	9	60	15.0	[7.1; 26.6]
Song_2024	0	27	0.0	[0.0; 12.8]
Thieblemont_2022	9	157	5.7	[2.7; 10.6]
**Random effects model**	**41**	**812**	**3.7**	**[1.9; 7.2]**
Heterogeneity: I^2 = 38.6%, p = 0.09				
**Random effects model**	**78**	**1667**	**4.0**	**[2.7; 5.9]**
Heterogeneity: I^2 = 26.7%, p = 0.13				
Test for subgroup differences: χ^2_1 = 0.08, df = 1 (p = 0.78)				

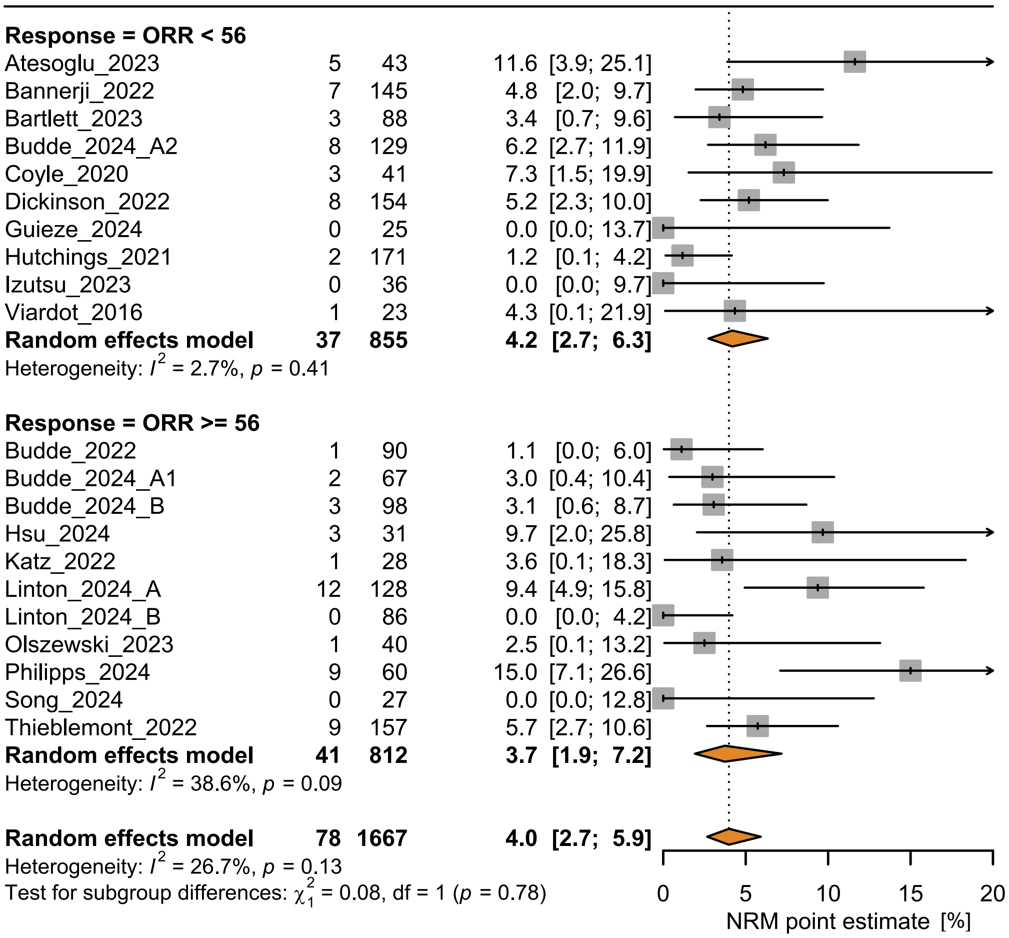

B

Study	Events	Total	Proportion	95%-CI
**Response = ORR < 64**				
Bahlis_2023	5	55	9.1	[3.0; 20.0]
D'Souza_2022	1	51	2.0	[0.0; 10.4]
Lesokhin_2023	14	123	11.4	[6.4; 18.4]
Moreau_2022	19	165	11.5	[7.1; 17.4]
**Random effects model**	**39**	**394**	**9.9**	**[7.3; 13.3]**
Heterogeneity: I^2 = 13.4%, p = 0.33				
**Response = ORR >= 64**				
Bumma_2024	6	117	5.1	[1.9; 10.8]
Chari_2022_A	0	30	0.0	[0.0; 11.6]
Chari_2022_B	3	44	6.8	[1.4; 18.7]
Dima_2024	3	106	2.8	[0.6; 8.0]
**Random effects model**	**12**	**297**	**4.0**	**[2.3; 7.0]**
Heterogeneity: I^2 = 0%, p = 0.73				
**Random effects model**	**51**	**691**	**6.1**	**[3.7; 10.0]**
Heterogeneity: I^2 = 37.8%, p = 0.13				
Test for subgroup differences: χ^2_1 = 7.98, df = 1 (p = 0.005)				

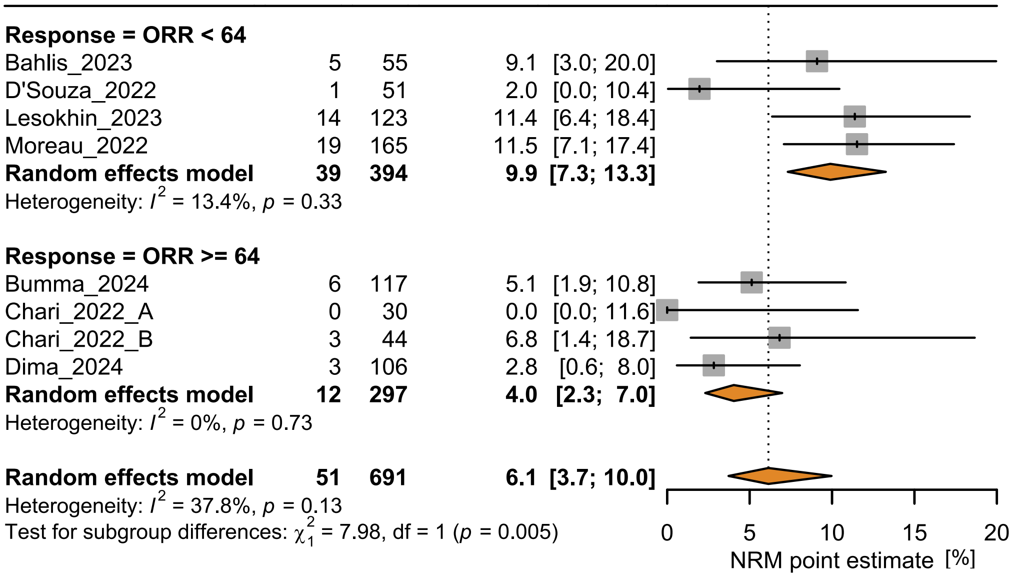

Figure S7. Subgroup analysis of NRM point estimates stratified by overall response rate. Forest plot illustrating non-relapse mortality (NRM) point estimates and 95% confidence intervals (95% CI) stratified by the median overall response rate (ORR) for B-Non-Hodgkin Lymphoma (A) and multiple myeloma (B). The random effects model results and heterogeneity measures are depicted for cohorts with an ORR below and above the median. Studies not reporting ORR were excluded from analysis (Goebeler et al, 2016, J Clin Oncol; Kim et al, 2024, Ann Oncol; Lebreton, 2024, Br J Haematol).

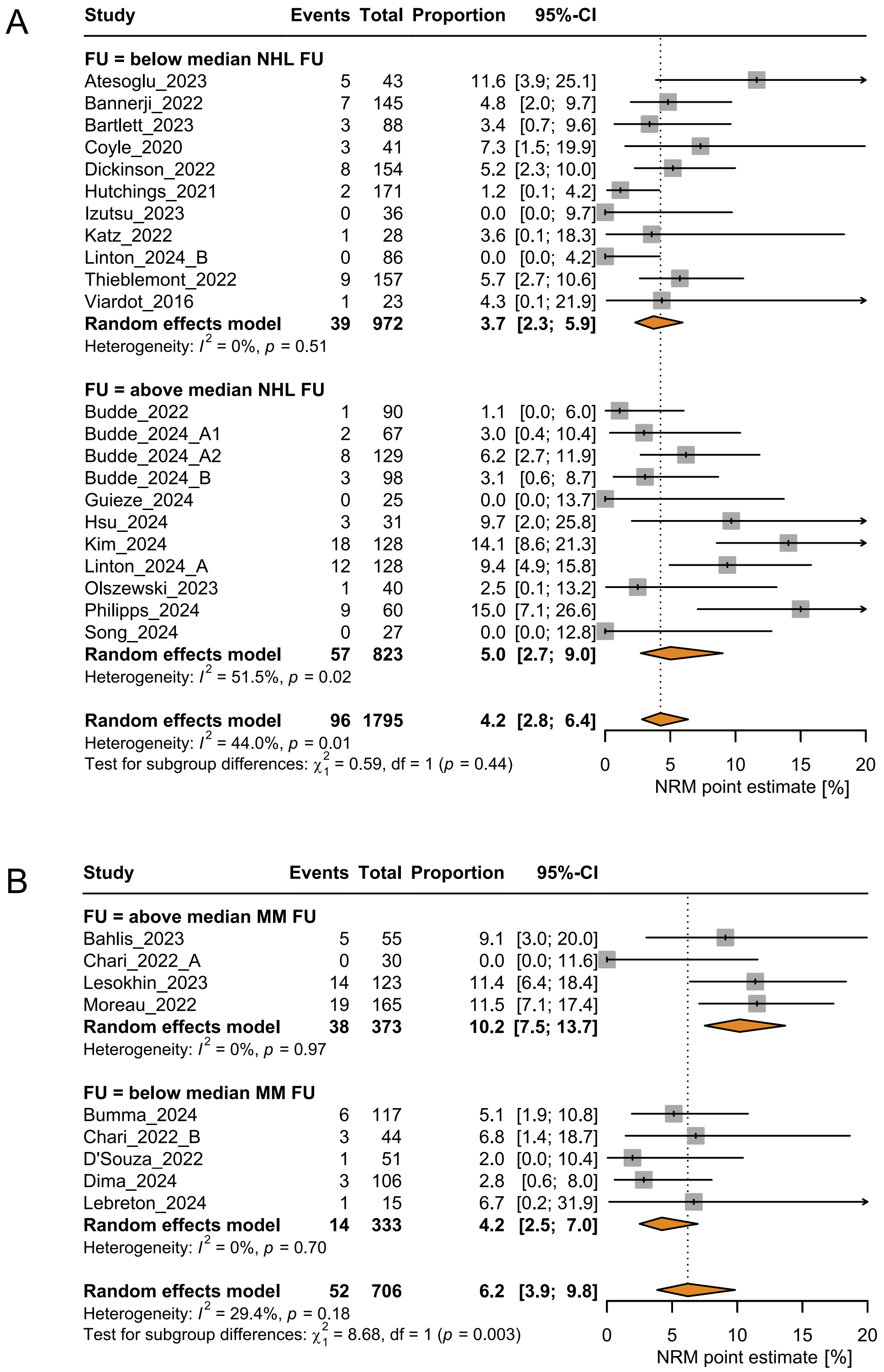

Figure S8. Subgroup analysis of NRM point estimates stratified by median follow-up time. Forest plot illustrating non-relapse mortality (NRM) point estimates and 95% confidence intervals (95% CI) stratified by the median follow-up (FU) time of each study cohort for B-Non-Hodgkin Lymphoma (A) and multiple myeloma (B). The random effects model results and heterogeneity measures are depicted for the cohorts below and above the median FU of 12 months. One study was excluded from analysis since no FU was reported (Goebeler et al, 2016, J Clin Oncol).

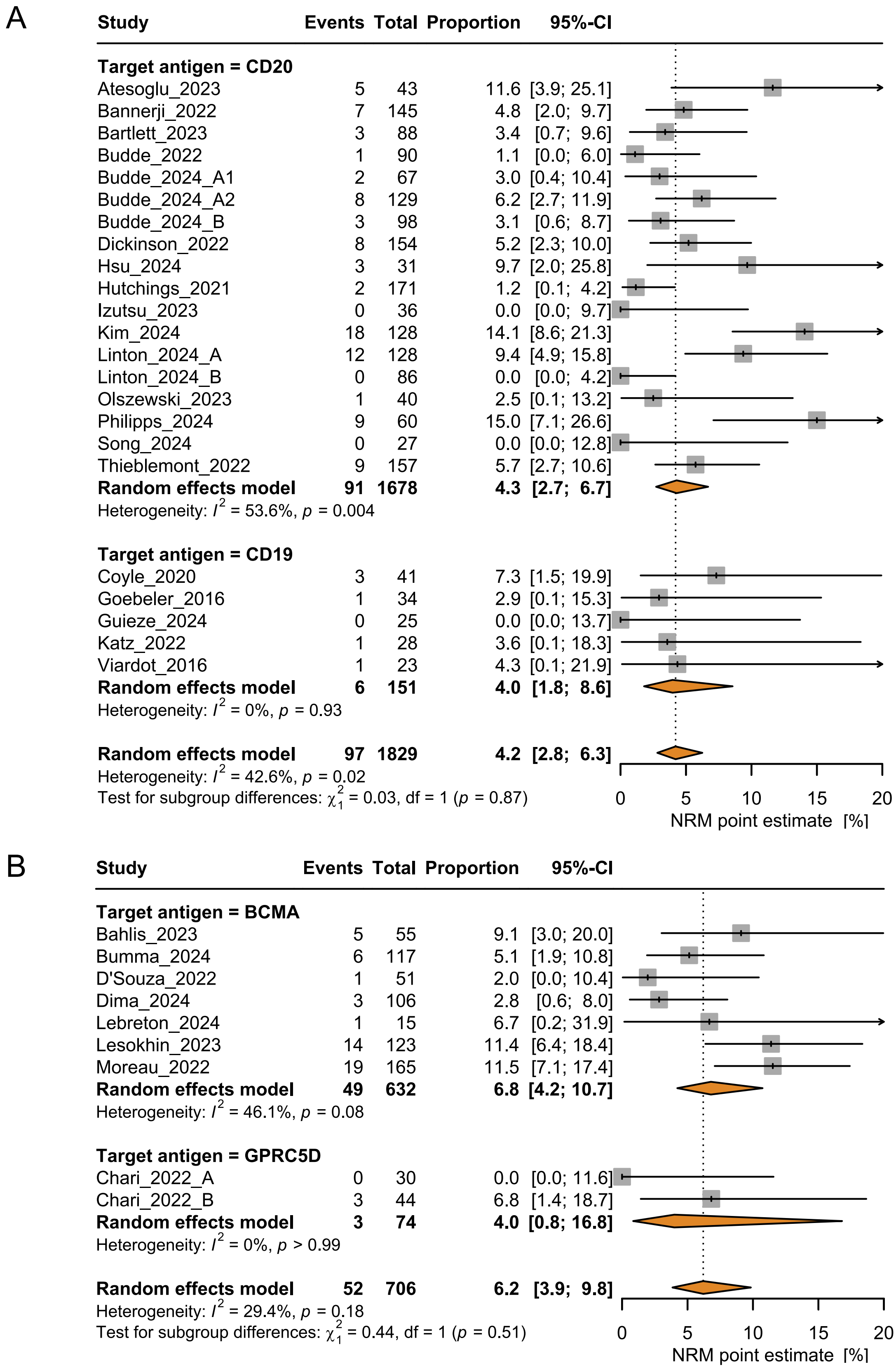

Figure S9. Subgroup analysis of NRM point estimates stratified by target antigen. Forest plot illustrating non-relapse mortality (NRM) point estimates and 95% confidence intervals (95% CI) stratified by target antigen for B-Non-Hodgkin Lymphoma (A) and multiple myeloma (B). The random effects model results and heterogeneity measures are depicted for the subcohorts.

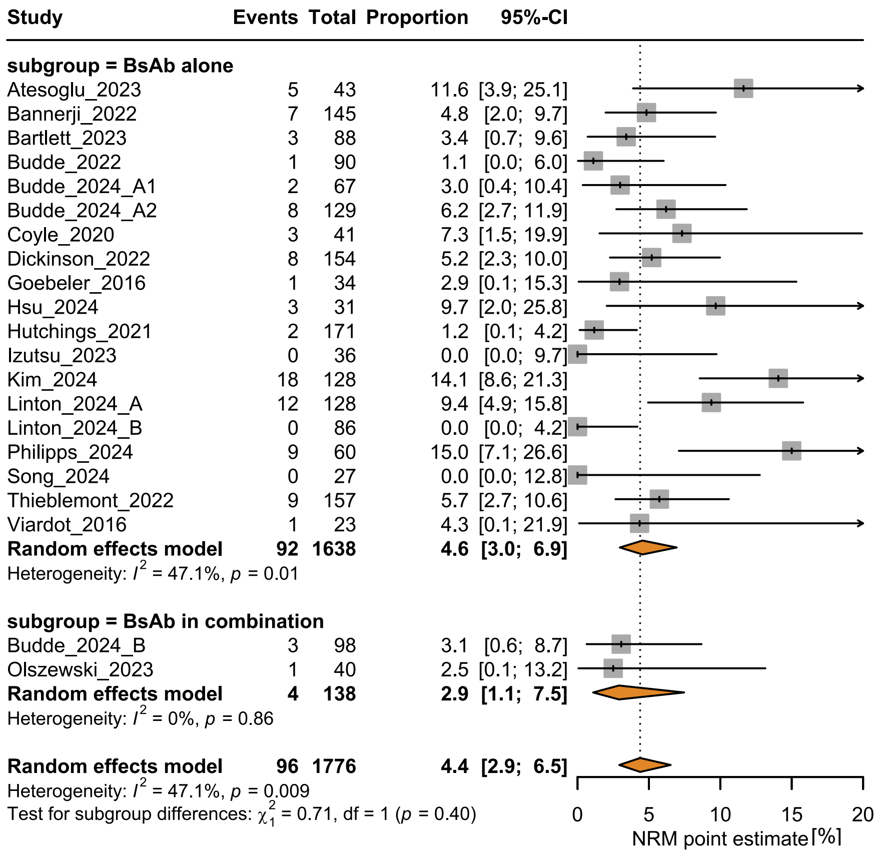

Figure S10. Subgroup analysis of NRM point estimates stratified by treatment mode in NHL cohorts. Forest plot illustrating non-relapse mortality (NRM) point estimates and 95% confidence intervals (95% CI) stratified by treatment mode of each study cohort for B-Non-Hodgkin Lymphoma (NHL). The random effects model results and heterogeneity measures are depicted for the cohorts treated with BsAb monotherapy vs. combination therapy. Two studies investigating BsAb therapy as consolidation therapy was excluded from analysis (Katz et al, 2022, Leuk Lymphoma and Guieze et al, 2024, Nat Commun).

Study	Events	Total	Proportion	95%-CI
**subgroup = Approved drug**				
Atesoglu_2023	5	43	11.6	[3.9; 25.1]
Bahlis_2023	5	55	9.1	[3.0; 20.0]
Bannerji_2022	7	145	4.8	[2.0; 9.7]
Bartlett_2023	3	88	3.4	[0.7; 9.6]
Budde_2022	1	90	1.1	[0.0; 6.0]
Budde_2024_A1	2	67	3.0	[0.4; 10.4]
Budde_2024_A2	8	129	6.2	[2.7; 11.9]
Budde_2024_B	3	98	3.1	[0.6; 8.7]
Chari_2022_A	0	30	0.0	[0.0; 11.6]
Chari_2022_B	3	44	6.8	[1.4; 18.7]
Dickinson_2022	8	154	5.2	[2.3; 10.0]
Dima_2024	3	106	2.8	[0.6; 8.0]
Hsu_2024	3	31	9.7	[2.0; 25.8]
Hutchings_2021	2	171	1.2	[0.1; 4.2]
Izutsu_2023	0	36	0.0	[0.0; 9.7]
Kim_2024	18	128	14.1	[8.6; 21.3]
Lebreton_2024	1	15	6.7	[0.2; 31.9]
Lesokhin_2023	14	123	11.4	[6.4; 18.4]
Linton_2024_A	12	128	9.4	[4.9; 15.8]
Linton_2024_B	0	86	0.0	[0.0; 4.2]
Moreau_2022	19	165	11.5	[7.1; 17.4]
Olszewski_2023	1	40	2.5	[0.1; 13.2]
Philipps_2024	9	60	15.0	[7.1; 26.6]
Song_2024	0	27	0.0	[0.0; 12.8]
Thieblemont_2022	9	157	5.7	[2.7; 10.6]
**Random effects model**	**136**	**2216**	**4.9**	**[3.4; 7.0]**
Heterogeneity: I^2 = 47.4%, p = 0.005				
**subgroup = Non-approved drug**				
Bumma_2024	6	117	5.1	[1.9; 10.8]
Coyle_2020	3	41	7.3	[1.5; 19.9]
D'Souza_2022	1	51	2.0	[0.0; 10.4]
Goebeler_2016	1	34	2.9	[0.1; 15.3]
Guieze_2024	0	25	0.0	[0.0; 13.7]
Katz_2022	1	28	3.6	[0.1; 18.3]
Viardot_2016	1	23	4.3	[0.1; 21.9]
**Random effects model**	**13**	**319**	**4.1**	**[2.4; 6.9]**
Heterogeneity: I^2 = 0%, p = 0.94				
**Random effects model**	**149**	**2535**	**4.7**	**[3.4; 6.4]**
Heterogeneity: I^2 = 39.2%, p = 0.01				
Test for subgroup differences: χ^2_1 = 0.30, df = 1 (p = 0.58)				

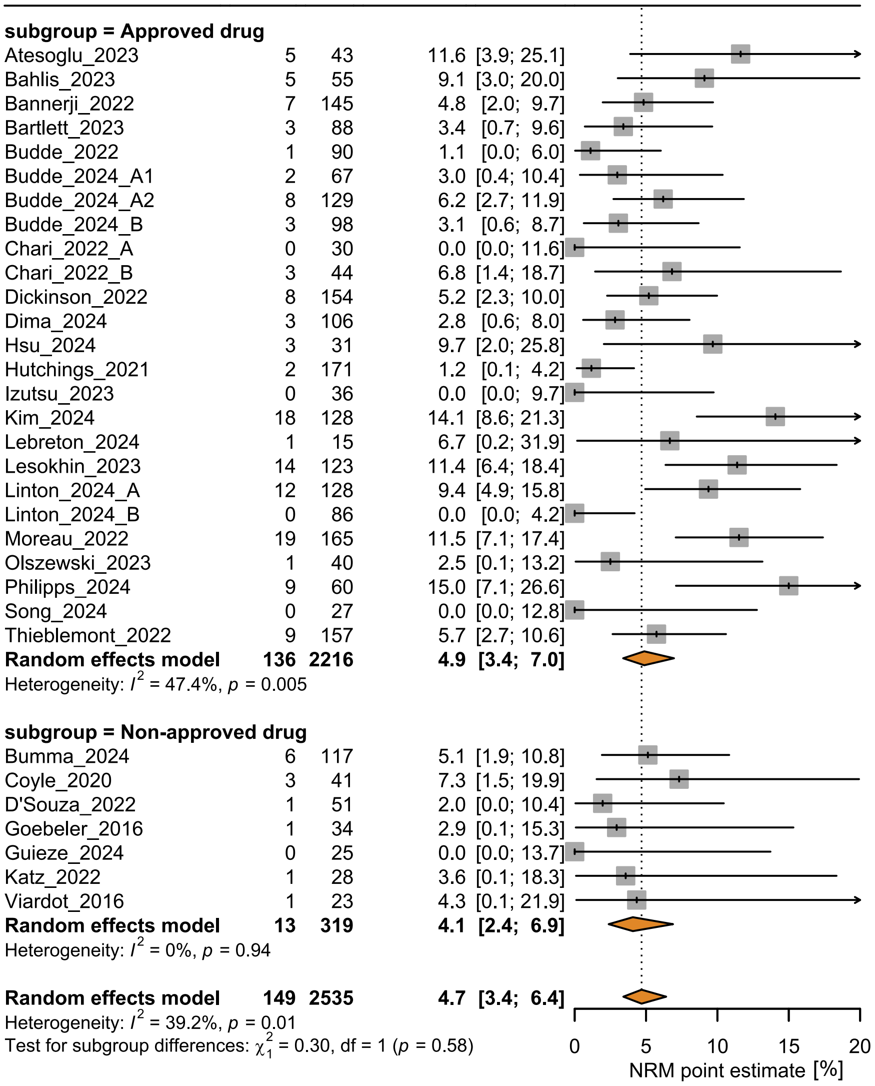

Figure S11. Subgroup analysis of NRM point estimates stratified by FDA and/or EMA approval. Forest plot illustrating non-relapse mortality (NRM) point estimates and 95% confidence intervals (95% CI) stratified by FDA/EMA approval for the investigated entity. The random effects model results and heterogeneity measures are depicted for the cohorts treated with products that are already approved by FDA or EMA and those not yet approved for the investigated entity by neither FDA nor EMA.

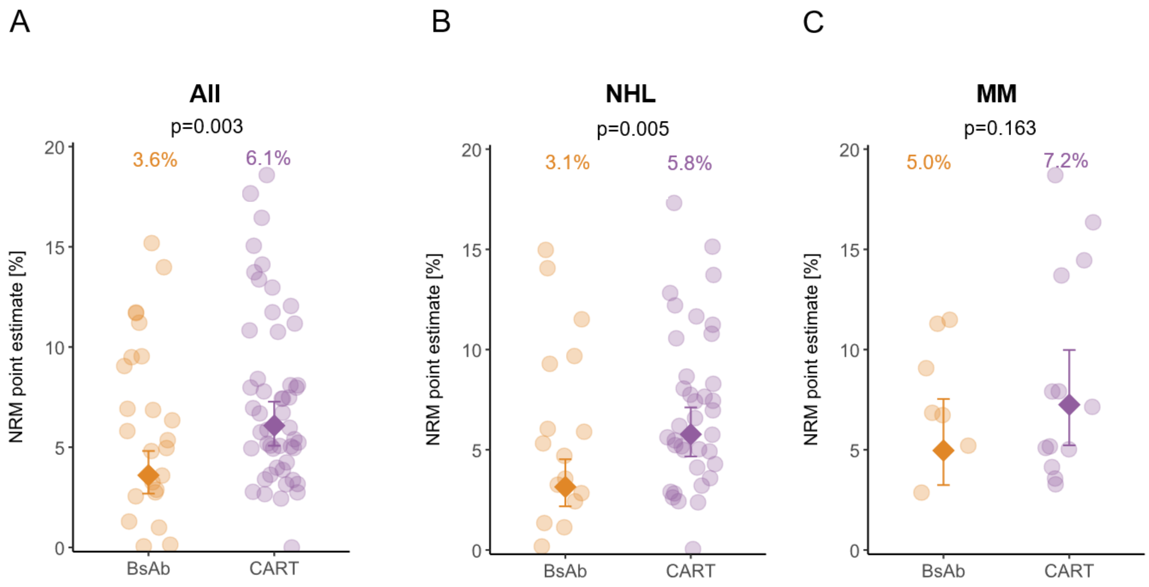

Figure S12. Comparative Analysis of NRM point estimates between BsAb and CAR T-cell therapy without COVID-19-related deaths. A-C Aggregated NRM point estimates and 95% confidence intervals are compared between BsAb- and CAR-T-treated patients across all disease entities (A) and specifically for NHL (B) and MM (C). NRM point estimates are calculated after exclusion of COVID-19-related deaths. P-value resembles test for subgroup heterogeneity. Abbreviations: NRM = non-relapse mortality; BsAb = bispecific antibody.

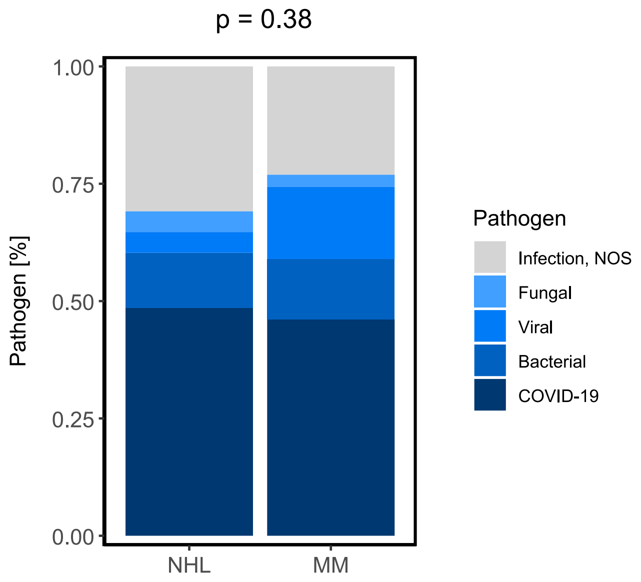

Figure S13. Distribution of pathogens responsible for fatal infections between patients with B-Non-Hodgkin-Lymphoma (NHL) and multiple myeloma (MM). Statistical significance was evaluated using the χ^2 distribution test. Abbreviations: NOS = not otherwise specified.

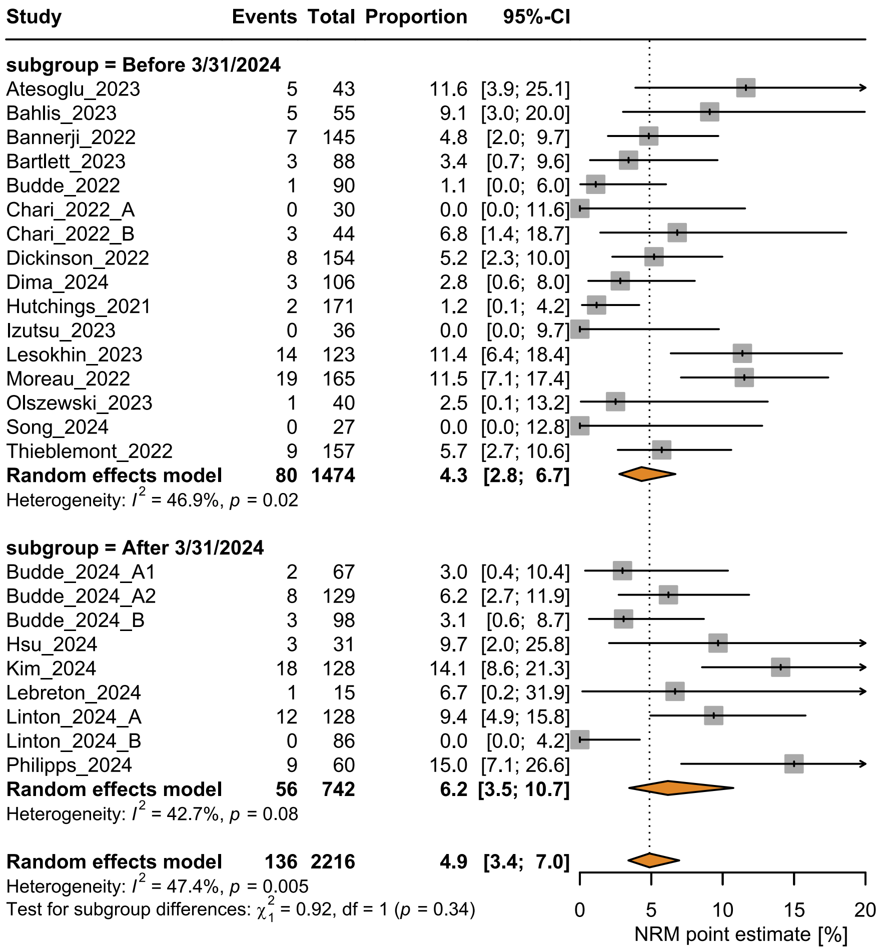

Figure S14. Subgroup analysis of NRM point estimates stratified by publication date. Forest plot illustrating non-relapse mortality (NRM) point estimates and 95% confidence intervals (95% CI) stratified by publication date. The random effects model results and heterogeneity measures are depicted for the records published before vs. after March 31st, 2024.

Table S1. Joanna-Brigg's Institute assessment of study bias.

First Author	Year	Criterion 1: Inclusion criteria	Criterion 2: measurement of mortality/ causes of death	Criterion 3: identification of mortality/ causes of death	Criterion 4: Consecutive Inclusion	Criterion 5: Complete Inclusion	Criterion 6: demographics (especially age, sex and ethnicity)	Criterion 7: Clinical information	Criterion 8: Follow-Up results
Linton	2024	yes	yes	yes	yes	yes	no	yes	yes
Budde	2024A	yes	yes	yes	yes	yes	no	yes	yes
Budde	2022	yes	yes	yes	yes	yes	yes	yes	yes
Kim	2024	yes	yes	yes	yes	yes	yes	yes	yes
Guieze	2024	yes	yes	yes	yes	yes	no	yes	yes
Coyle	2020	yes	yes	yes	yes	yes	yes	yes	yes
Viardot	2016	yes	yes	yes	yes	yes	no	yes	yes
Katz	2022	yes	yes	yes	yes	yes	no	yes	yes
Izutsu	2023	yes	yes	yes	yes	yes	yes	yes	yes
Thieblemont	2022	yes	yes	yes	yes	yes	yes	yes	yes
Hsu	2024	yes	yes	yes	yes	yes	no	yes	yes
Song	2024	yes	yes	yes	yes	yes	yes	yes	yes
Atsoglu	2023	yes	yes	yes	yes	yes	yes	yes	yes
Dickinson	2022	yes	yes	yes	yes	yes	yes	yes	yes
Budde	2024B	yes	yes	yes	yes	yes	no	yes	yes
Olszewski	2023	yes	yes	yes	yes	yes	no	yes	yes

Bartlett	2023	yes	yes	yes	yes	yes	no	yes	yes
Philipps	2024	yes	yes	yes	yes	yes	no	yes	yes
Goebeler	2016	yes	yes	yes	yes	yes	no	yes	no
Hutchings	2021	yes	yes	yes	yes	yes	no	yes	yes
Bannerji	2022	yes	yes	yes	yes	yes	yes	yes	yes
D’Souza	2022	yes	yes	yes	yes	yes	yes	yes	yes
Bahlis	2023	yes	yes	yes	yes	yes	yes	yes	yes
Lesokhin	2023	yes	yes	yes	yes	yes	yes	yes	no
Bumma	2024	yes	yes	yes	yes	yes	yes	yes	yes
Chari	2022	yes	yes	yes	yes	yes	yes	yes	yes
Lebreton	2024	no	yes	yes	no	no	no	yes	yes
Dima	2024	unclear	yes	yes	unclear	unclear	yes	yes	yes
Moreau	2022	yes	yes	yes	yes	yes	yes	yes	yes

Joanna-Brigg’s Institute assessment of study bias (Munn et al. JBI Evid Synth. 2020 Oct;18(10):2127-2133). Criterion 9 (presenting sites’/clinics’ demographics) was assessed within criterion 5 (demographics of the participants in the study). Criterion 10 (statistical analysis) was not applicable since the extracted data processed in this meta-analysis are not based on statistical tools in the original studies.

Table S2. Multivariable meta-regression analysis excluding COVID-related deaths.

	Studies	Patients	Estimate (95%CI)	p value
**Mechanism**				
- CAR-T [ref.]	45	6692		
- BsAb	23	1900	-0.35(-0.77;0.07)	0.10
**Entity**				
- IL [ref.]	8	764		
- LBCL	34	5697	-0.01 (-0.58;0.60)	0.98
- MCL	6	400	0.43 (-0.32;1.18)	0.25
- MM	20	1731	0.43 (-0.20;1.06)	0.18
**Prior treatment**				
- Treatment lines [median]	68	8592	-0.02(-0.18;0.14)	0.82
**Treatment setting**				
- CT [ref.]	36	3704		
- RW	32	4888	0.28 (-0.22;0.78)	0.27
**Follow-up**				
- Follow-up [years]	68	8592	0.24 (0.05;0.44)	**0.02**

Abbreviations: BsAb = Bispecific antibodies, IL = Indolent lymphoma, LBCL = Large B-cell lymphoma, MCL = Mantle cell lymphoma, MM = multiple myeloma, CT = clinical trial, RW = real-world study

Table S3. Causes of death classified as "others".

Case	Author	Year	Cause of Death
1	Bahlis	2023	Sudden death
2	Lesokhin	2023	Failure to thrive
3	Dickinson	2022	Delirium
4	Bannerji	2022	Gastric perforation
5	Bannerji	2022	Tumor lysis syndrome
6	Thieblemont	2022	Loss of consciousness
7	Thieblemont	2022	General health deterioration
8	Coyle	2020	Pancytopenia

Table S4. Extended characteristics of included BsAb records.

Entity	First Author	Year	Cohort	Product	Setting	Time Frame of Inclusion	Patients receiving immunoglobulins [%]
IL	Linton	2024	A	Epcoritamab	II	06/20-04/23	NR
	Linton	2024	B	Epcoritamab	II	10/22-01/24	NR
	Budde	2024	A1	Mosunetuzumab	I/II	09/15-05/19	NR
	Budde	2022		Mosunetuzumab	II	05/19-09/20	NR
	Kim	2024		Odronextamab	II	12/19-07/22	NR
LBCL	Guieze	2024		Blinatumomab	II	07/19-07/21	NR
	Coyle	2020		Blinatumomab	II	01/17-01/18	NR
	Viardot	2016		Blinatumomab	II	08/12-07/14	NR
	Katz	2022		Blinatumomab	II	03/17-05/18	NR
	Izutsu	2023		Epcoritamab	II	01/21-01/22	NR
	Thieblemont	2022		Epcoritamab	I/II	06/20-01/22	NR
	Hsu	2024		Glofitamab	RW	01/21-10/22	NR
	Song	2024		Glofitamab	I	NR/NR-12/22	NR
	Atesoglu	2023		Glofitamab	RW	03/21-09/22	NR
	Dickinson	2022		Glofitamab	II	01/20-09/21	NR
	Budde	2024	A2	Mosunetuzumab	I/II	09/15-05/19	NR
	Budde	2024	B	Mosunetuzumab	II	09/18-02/22	NR
	Olszewski	2023		Mosunetuzumab	II	11/19-07/20	NR
	Bartlett	2023		Mosunetuzumab	II	04/19-02/20	NR
MCL	Philipps	2024		Glofitamab	I/II	NR/NR-09/23	NR
NHL	Goebeler	2016		Blinatumomab	I	06/04-07/11	NR
	Hutchings	2021		Glofitamab	I	NR/NR-08/20	NR
	Bannerji	2022		Odronextamab	I	02/15-09/21	NR

	D'Souza	2022		ABBV-383 (Etentamig)	I	NR/NR-01/22	NR
	Bahlis	2023		Elranatamab	I	11/17-04/21	32.7
	Lesokhin	2023		Elranatamab	II	02/21-01/22	43.1
	Bumma	2024		Linvoseltamab	I/II	01/19-10/22	NR
MM	Chari	2022	A	Talquetamab	I	01/18-11/21	NR
	Chari	2022	B	Talquetamab	I	01/18-11/21	NR
	Lebreton	2024		Teclistamab	RW	11/22-10/23	53.3
	Dima	2024		Teclistamab	RW	08/22-08/23	41.5
	Moreau	2022		Teclistamab	I/II	03/20-08/21	39.4

Abbreviations: IL = Indolent lymphoma, LBCL = Large B-cell lymphoma, MCL = Mantle cell lymphoma, MM = multiple myeloma, CT = clinical trial, RW = real-world study, NR = not reported.

Table S5. Sensitivity analyses.

	**Reported results:**<br><br>Random effects model including all studies	**Sensitivity analysis 1:**<br><br>Fixed effects model including all studies	**Sensitivity analysis 2:**<br><br>Random effects model excluding COVID-related deaths	**Sensitivity analysis 3:**<br><br>Random effects model excluding studies with dose escalation cohorts
**Overall NRM BsAb**	4.7 (3.4-6.4)	5.9 (5.0-6.9)	3.5 (2.6-4.6)	5.0 (3.5-7.0)
**Entity**				
- NHL	4.2 (2.8-6.3)	5.3 (4.4-6.4)	3.2 (2.3-4.5)	4.4 (2.7-7.0)
- MM	6.2 (3.9-9.8)	7.4 (5.7-9.5)	4.4 (2.7-7.1)	NA
**Overall NRM CAR-T**	6.8 (5.8-8.0)	7.5 (7.0-8.1)	NA	NA

Abbreviations: NRM = Non-relapse mortality [%], BsAb = Bispecific antibodies.

Table S6. Causes of death classified as "infections".

Case	First Author	Year	Cohort	Entity	Cause of Death
1	Atesoglu	2023		NHL	COVID-19
2	Atesoglu	2023		NHL	COVID-19
3	Atesoglu	2023		NHL	Sepsis from gram-negative bacteria
4	Bahlis	2023		MM	COVID-19
5	Bahlis	2023		MM	COVID-19
6	Bahlis	2023		MM	Septic shock
7	Bahlis	2023		MM	Adenoviral infection
8	Bannerji	2022		NHL	Pneumonia
9	Bannerji	2022		NHL	Pneumocystis jirovecii pneumonia
10	Bannerji	2022		NHL	COVID-19
11	Bartlett	2023		NHL	Pneumonia
12	Bartlett	2023		NHL	Sepsis
13	Bartlett	2023		NHL	Cholangitis in neutropenic fever
14	Budde	2024	A1	NHL	Pneumonia
15	Budde	2024	A2	NHL	Sepsis
16	Budde	2024	A2	NHL	Bacteremia due to bowel perforation
17	Budde	2024	A2	NHL	Sepsis
18	Budde	2024	B	NHL	COVID-19
19	Budde	2024	B	NHL	COVID-19
20	Budde	2024	B	NHL	Pneumonia
21	Bumma	2024		MM	Pneumocystis jirovecii pneumonia
22	Bumma	2024		MM	Progressive multifocal leukoencephalopathy
23	Bumma	2024		MM	Sepsis from P. aeruginosa
24	Bumma	2024		MM	Infection
25	Bumma	2024		MM	Infection
26	Chari	2022	B	MM	Sepsis
27	Coyle	2020		NHL	Sepsis
28	Dickinson	2022		NHL	COVID-19
29	Dickinson	2022		NHL	COVID-19
30	Dickinson	2022		NHL	COVID-19
31	Dickinson	2022		NHL	COVID-19
32	Dickinson	2022		NHL	COVID-19
33	Dickinson	2022		NHL	Sepsis
34	Dickinson	2022		NHL	Sepsis
35	Dima	2024		MM	COVID-19
36	Dima	2024		MM	Pneumonia from rhino/adenovirus
37	Dima	2024		MM	Sepsis
38	D'Souza	2022		MM	COVID-19
39	Goebeler	2016		NHL	Pneumocystis jirovecii pneumonia
40	Hsu	2024		NHL	COVID-19
41	Hsu	2024		NHL	Sepsis from bacteria
42	Hutchings	2021		NHL	Septic shock
43	Katz	2022		NHL	infection
44	Kim	2024		NHL	Pneumonia
45	Kim	2024		NHL	Pneumonia

46	Kim	2024		NHL	Pneumonia
47	Kim	2024		NHL	Sepsis
48	Kim	2024		NHL	Systemic mycosis
49	Kim	2024		NHL	Progressive multifocal leukoencephalopathy
50	Kim	2024		NHL	Pneumonia from P. aeruginosa
51	Kim	2024		NHL	Sepsis from E. coli
52	Kim	2024		NHL	Progressive multifocal leukoencephalopathy
53	Kim	2024		NHL	COVID-19
54	Kim	2024		NHL	COVID-19
55	Kim	2024		NHL	COVID-19
56	Kim	2024		NHL	COVID-19
57	Kim	2024		NHL	COVID-19
58	Kim	2024		NHL	COVID-19
59	Kim	2024		NHL	COVID-19
60	Kim	2024		NHL	COVID-19
61	Lebreton	2024		MM	Bacterial infection
62	Lesokhin	2023		MM	Adenoviral hepatitis
63	Lesokhin	2023		MM	Pneumonia from adenovirus
64	Lesokhin	2023		MM	Pneumonia from P. aeruginosa
65	Lesokhin	2023		MM	COVID-19
66	Lesokhin	2023		MM	COVID-19
67	Lesokhin	2023		MM	Infection
68	Lesokhin	2023		MM	Infection
69	Lesokhin	2023		MM	Infection
70	Linton	2024	A	NHL	COVID-19
71	Linton	2024	A	NHL	COVID-19
72	Linton	2024	A	NHL	COVID-19
73	Linton	2024	A	NHL	COVID-19
74	Linton	2024	A	NHL	COVID-19
75	Linton	2024	A	NHL	COVID-19
76	Linton	2024	A	NHL	Sepsis from P. aeruginosa
77	Linton	2024	A	NHL	Organizing pneumonia
78	Linton	2024	A	NHL	Pneumonia
79	Moreau	2022		MM	COVID-19
80	Moreau	2022		MM	COVID-19
81	Moreau	2022		MM	COVID-19
82	Moreau	2022		MM	COVID-19
83	Moreau	2022		MM	COVID-19
84	Moreau	2022		MM	COVID-19
85	Moreau	2022		MM	COVID-19
86	Moreau	2022		MM	COVID-19
87	Moreau	2022		MM	COVID-19
88	Moreau	2022		MM	COVID-19
89	Moreau	2022		MM	Pneumonia
90	Moreau	2022		MM	Pneumonia from P. aeruginosa
91	Moreau	2022		MM	COVID-19
92	Moreau	2022		MM	COVID-19

93	Moreau	2022	MM	Pneumonia from streptococci
94	Moreau	2022	MM	Progressive multifocal leukoencephalopathy
95	Olszewski	2023	NHL	Pneumonia
96	Philipps	2024	NHL	COVID-19
97	Philipps	2024	NHL	COVID-19
98	Philipps	2024	NHL	COVID-19
99	Philipps	2024	NHL	COVID-19
100	Philipps	2024	NHL	COVID-19
101	Philipps	2024	NHL	COVID-19
102	Philipps	2024	NHL	Pneumonia
103	Philipps	2024	NHL	Septic shock
104	Thieblemont	2022	NHL	COVID-19
105	Thieblemont	2022	NHL	COVID-19
106	Thieblemont	2022	NHL	Progressive multifocal leukoencephalopathy
107	Viardot	2016	NHL	Pneumonia

Further Supplemental Material:

Study Protocol
